# HLA DNA Sequence Variation among Human Populations: Molecular Signatures of Demographic and Selective Events

**DOI:** 10.1371/journal.pone.0014643

**Published:** 2011-02-01

**Authors:** Stéphane Buhler, Alicia Sanchez-Mazas

**Affiliations:** Laboratory of Anthropology, Genetics and Peopling History, Department of Anthropology, University of Geneva, Geneva, Switzerland; St. Petersburg Pasteur Institute, Russian Federation

## Abstract

Molecular differences between HLA alleles vary up to 57 nucleotides within the peptide binding coding region of human Major Histocompatibility Complex (MHC) genes, but it is still unclear whether this variation results from a stochastic process or from selective constraints related to functional differences among HLA molecules. Although HLA alleles are generally treated as equidistant molecular units in population genetic studies, DNA sequence diversity among populations is also crucial to interpret the observed HLA polymorphism. In this study, we used a large dataset of 2,062 DNA sequences defined for the different HLA alleles to analyze nucleotide diversity of seven HLA genes in 23,500 individuals of about 200 populations spread worldwide. We first analyzed the HLA molecular structure and diversity of these populations in relation to geographic variation and we further investigated possible departures from selective neutrality through Tajima's tests and mismatch distributions. All results were compared to those obtained by classical approaches applied to HLA allele frequencies.

Our study shows that the global patterns of HLA nucleotide diversity among populations are significantly correlated to geography, although in some specific cases the molecular information reveals unexpected genetic relationships. At all loci except HLA-DPB1, populations have accumulated a high proportion of very divergent alleles, suggesting an advantage of heterozygotes expressing molecularly distant HLA molecules (asymmetric overdominant selection model). However, both different intensities of selection and unequal levels of gene conversion may explain the heterogeneous mismatch distributions observed among the loci. Also, distinctive patterns of sequence divergence observed at the HLA-DPB1 locus suggest current neutrality but old selective pressures on this gene. We conclude that HLA DNA sequences advantageously complement HLA allele frequencies as a source of data used to explore the genetic history of human populations, and that their analysis allows a more thorough investigation of human MHC molecular evolution.

## Introduction

The Human Leukocyte Antigen (HLA) loci are among the most polymorphic genes currently described in the human genome, with more than 4,000 observed alleles according to release 2.27.1 of IMGT/HLA database. These loci are located on the short arm of chromosome 6 within the Major Histocompatibility Complex (MHC) and are extensively studied due to their critical role in organ or hematopoietic stem cell transplantation and clinical medicine. Allelic variation of HLA genes is characterized at the DNA sequence level since the mid of 1990s [1], [2]. This approach started with the development of molecular typing methods for class II loci (e.g. PCR-SSP, direct or reverse PCR-SSO, PCR-SSCP, and SBT), and more recently for class I loci, HLA-Cw being the last gene studied with such typing strategies [3], [4], [5], [6], [7]. The characterization and classification of alleles follow strict rules recommended by the WHO HLA Nomenclature Committee For Factor of the HLA System [8], [9], and make use of a hierarchic numerical code for allele naming introduced shortly after the 10^th^ Histocompatibility Workshop and Conference. The DNA sequence of each HLA allele is now accessible to the whole scientific community through an online database which also provides genomic and coding sequence alignments for every locus, nomenclature guidelines, complete listings of alleles and new allele reports, among other information [8], [10].

Despite the availability of such an important amount of data, thus far the information on HLA nucleotide sequences has been mainly used to investigate the evolutionary history of the human MHC [11], [12], [13], [14], [15], [16], [17], [18] or to interpret individual HLA genotypic profiles for clinical (i.e. transplantation) purposes, but it has seldom been used to study the genetic variation among populations. Many studies have shown that the HLA polymorphism is very informative to reconstruct past human migration events [19], [20], [21], [22], [23], [24], [25]. However, these works are based on the interpretation of allelic frequency distributions, with all alleles being considered as equidistant molecular units in each population; the main reason being that there is still a lot of uncertainty associated to HLA molecular typings due to the extreme level of polymorphism at these loci. Indeed, HLA typings usually generate numerous ambiguities which correspond to situations where either several allelic pairs (i.e. potential genotypes) can explain the reactivity profile obtained for an individual, or the resolution of the typing protocol is too low to allow discrimination between some alleles. As a consequence, establishing a direct relationship between “allele names” (generally, groups including several ambiguous alleles) and their corresponding nucleotide sequences has been very hazardous, preventing the use of the latter in population studies. Moreover, when a study includes many population samples that were not characterized by the same molecular typing methods, the existence of different ambiguity patterns in different population samples further increases the complexity of the data.

However, new perspectives have emerged to use the HLA nucleotide sequence information for anthropological purposes: firstly, through large scale homogeneous treatments of ambiguous genotypes in population samples by the *International Histocompatibility Working Group – Anthropology/Human Genetic Diversity* (IHWG-AHGD) component of the 13^th^ Histocompatibility Workshop and Conference [26]; and secondly, through the recent development of computer programs (i.e. Gene[rate] tools at http://geneva.unige.ch/generate/) that implement powerful methods of allelic frequency estimation on data that contain genotyping ambiguities [27], [28], [29], [30] within the scope of the *Analysis of HLA Population Data* (AHPD) component of the 15^th^ Histocompatibility Workshop and Conference [31] and of the HLA-NET European COST project (http://w3.cost.esf.org/index.php?id=212&action_number=BM0803).

In this study we analyzed the nucleotide diversity of seven HLA genes (HLA-A, -B, -Cw, -DPB1, -DQA1, -DQB1, and -DRB1) in more than 23'500 individuals from about two hundred populations of all continents (Table 1 and File S1). We first applied an extensive treatment of the data at the genotypic and nucleotide levels to ensure compatibility with the latest updates of the official HLA allele nomenclature. We then analyzed the DNA molecular variation of HLA at several geographic scales (i.e. worldwide, continental, and regional) to investigate its congruence with the observed genetic diversity profiles based on allelic frequencies and explore the additional information brought by DNA sequences. Despite the complex evolution of the HLA system and the difficulty to disentangle the effects of molecular mechanisms such as balancing selection, gene conversion and recombination, our results suggest a strong influence of demographic factors and past human migrations on its DNA polymorphism. Nevertheless, natural selection acts by maintaining highly divergent alleles within populations, probably as a consequence of asymmetric overdominance of heterozygote individuals. Furthermore, ancient traces of selective pressures were detected in DNA sequences of HLA-DPB1, a locus whose evolution is usually assumed to be close to neutral expectations.

**Table 1:** Summary of the population and DNA sequence data used in this study.

Locus	SAF^1^	NAF^1^	EUR^1^	SWA^1^	NEA^1^	SEA^1^	PAC^1^	AUS^1^	NAM^1^	SAM^1^	OTH^1^	All^1^
**HLA-A**	12	2	8	20	3	24	8	4	5	4	7	97
**HLA-B**	9	2	8	21	2	23	5	4	5	4	7	90
**HLA-Cw**	8	0	4	15	2	20	4	4	3	3	5	68
**HLA-DRB1**	9	9	18	10	8	22	7	3	12	7	1	106
**HLA-DQA1**	7	3	17	2	3	3	4	2	12	5	0	58
**HLA-DQB1**	10	13	22	5	5	6	7	2	12	7	0	89
**HLA-DPB1**	7	0	14	3	4	6	7	3	8	4	0	56

Locus	Number of individuals^2^	Mean sample size (SD)	Number of sequences^3^	Number of nucleotide sites^4^
**HLA-A**	12,224	126 (134.2)	414 (466)	546
**HLA-B**	11,440	127.1 (138.4)	755 (845)	546
**HLA-Cw**	8,263	121.5 (132.9)	231 (269)	546
**HLA-DRB1**	11,848	111.8 (115.8)	438 (497)	270
**HLA-DQA1**	5,398	93.1 (45.7)	33 (46)	249
**HLA-DQB1**	9,255	104 (54.5)	70 (87)	270
**HLA-DPB1**	4,911	87.7 (46.1)	121 (221)	264

1 Number of populations.

2 The total number of individuals included in the analyses is around 23,500, as many individuals have been simultaneously typed at several loci.

3 Data loaded from the IMGT official database (release 2.13). In fact, the number of sequences used for the analyses is slightly higher (indicated within brackets) due to the automated conversions to lower levels of resolution (e.g. from 6 to 4 digits) applied to the data, as explained in Materials and methods.

4 Exons 2 and 3 for class I genes, exon 2 for class II genes.

## Results

### Nucleotide diversity and heterozygosity

**Figure 1:**
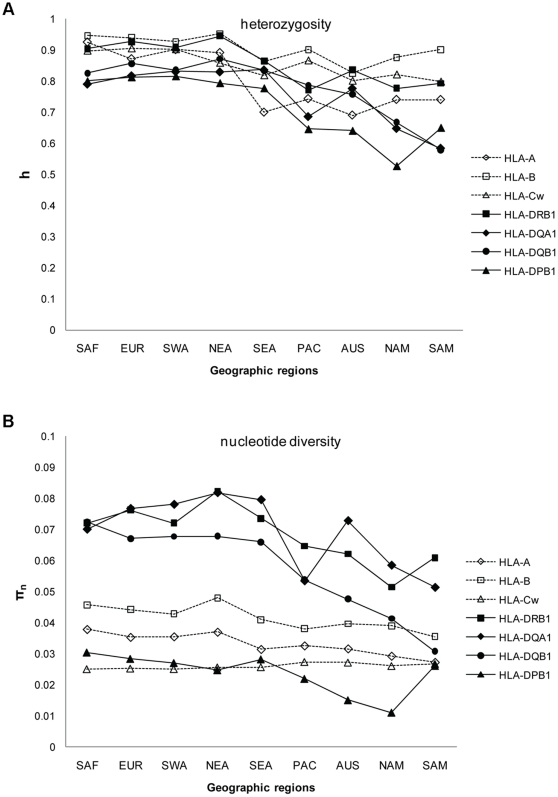
HLA genetic diversity within populations. A) heterozygosity (h) and B) nucleotide diversity (π_n_) within populations grouped according to their geographic location, at each locus under study. Only the regions represented by populations at every locus are illustrated on the graphs. See File S2 for detailed values (means and standard deviations). Sub-Saharan Africa (SAF), Europe (EUR), Southwest Asia (SWA), Northeast Asia (NEA), Southeast Asia (SEA), , Pacific (PAC), Australia (AUS), North America (NAM), and South America (SAM). See Supporting Information S1 for the list of populations included in each region.

Expected heterozygosity (h) and nucleotide diversity (π_n_) within populations averaged on each geographic region are given in File S2 and plotted in Figure 1A and 1B, respectively. Values ranged from 0.525 (HLA-DPB1 in NAM) to 0.961 (HLA-B in NAF) for h, and from 0.011 (HLA-DPB1 in NAM) to 0.082 (HLA-DQA1 and -DRB1 in NEA) for π_n_. For h, HLA-B was the most diversified and HLA-DPB1 the less diversified locus in almost all regions, but standard deviations overlapped between loci in many population groups (results not shown). For π_n_, on the contrary, a clear-cut difference appeared between loci, with HLA-DRB1, -DQA1 and -DQB1 (class II loci) exhibiting higher values than HLA-B, -A, -Cw (class I loci) and -DPB1 (class II locus) in all but one (SAM) geographic regions. Reduced diversity values of both π_n_ and h were observed in aboriginal populations from SEA (i.e. TW), as well as in PAC, AUS, NAM and SAM compared to EUR, SAF, NAF, SWA, NEA and CSEA, except for π_n_ at HLA-Cw. This reflects peculiar demographic histories for Taiwanese aborigines, Oceanian and Amerindian populations, which most certainly underwent rapid genetic drift due to small population sizes and geographic and/or cultural barriers.

### Distributions of mean pairwise differences between allele sequences within populations

**Figure 2:**
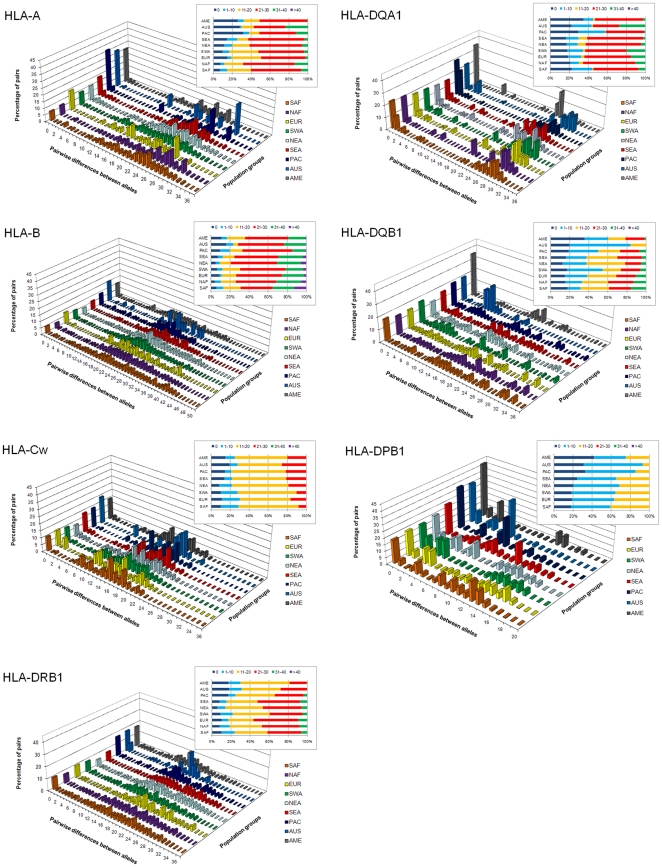
Distributions of pairwise differences between HLA sequences (mismatch distributions). Average mismatch distributions within populations grouped according to their geographical location, at each HLA locus under study. Histograms at the top-right indicate the proportion of allele pairs with 0, 1–10, 11–20, 21–30, 31–40 and more than 40 nucleotides of divergence within each group of populations.

Figure 2 shows the distributions of pairwise differences (i.e. mismatch distributions) between allelic DNA sequences of each HLA locus averaged on all populations in each geographic region. The proportion of sequence pairs diverging by 0, 1 to 10, 11 to 20, 21 to 30, 31 to 40, and more than 40 nucleotides is also plotted in a box at the top-right of each graphic.

HLA-B exhibited the highest number of nucleotide differences within PBR coding exons, with up to 51 nucleotides diverging between alleles, followed by HLA-A, -DRB1 and -DQB1 alleles (up to 38 diverging nucleotides), and HLA-DQA1 (34), -Cw (27) and -DPB1 (20).

Several peculiarities distinguished one locus from another, notably for HLA-DPB1 and -DQB1, both described hereafter, or for HLA-Cw compared to the other two class I loci -A and -B (i.e. less divergent alleles were observed for HLA-Cw). However, some similarities also appeared. First, there were higher proportions (up to twice) of identical allele pairs (i.e. class 0) in AME, PAC and AUS (and TW, results not shown) than in populations from EUR, SAF, NAF, SWA, NEA (and CSEA, results not shown). This result reflects the fact that Amerindian, Oceanian and Taiwanese aboriginal populations usually exhibited one or two alleles at high frequencies and only a small number of less frequent ones, probably as a consequence of rapid genetic drift. As each DNA sequence present in a population sample is compared to the others depending on their respective absolute frequency, the presence of one or two predominant alleles is weighting strongly on the percentage of pair comparisons included in class 0. By contrast, European, continental Asian and African populations generally showed many alleles with more even frequencies. Second, closely related alleles (<10 diverging nucleotides) were uncommon, while divergent ones (>20 diverging nucleotides) were abundant within all population groups, at most loci. This result was very clear for HLA-A, -B and -DQA1, with averages on all geographic regions of 57%, 71.6% and 58% of allele pairs differing by more than 20 nucleotides, respectively. HLA-Cw and -DRB1 also showed a pattern of low relatedness between alleles, but it was less pronounced (allele pairs differing by more than 20 nucleotides took values ranging from 8% at HLA-Cw in SAF to 57% at HLA-DRB1 in EUR, respectively). HLA-DQB1 exhibited a slightly to much higher proportion of related alleles compared to HLA-Cw and -DRB1, depending on the region of interest (values ranged from 0% in AUS to 40% in NAF for allele pairs differing by more than 20 nucleotides, respectively). HLA-DPB1 contrasted with the other loci as the proportion of sequence pairs that display 10 or less than 10 divergent nucleotides within population groups varied between 59.3% (SAF) and 92.9% (AUS). This can be related to the fact that HLA-DPB1 exhibited the lowest π_n_ and h in most geographic regions (see File S2 and Figure 1A and B), and may indicate a particularity of this locus with respect to balancing selection, as shown by other kinds of evidence (see next section and discussion).

### Tests of selective neutrality

**Figure 3:**
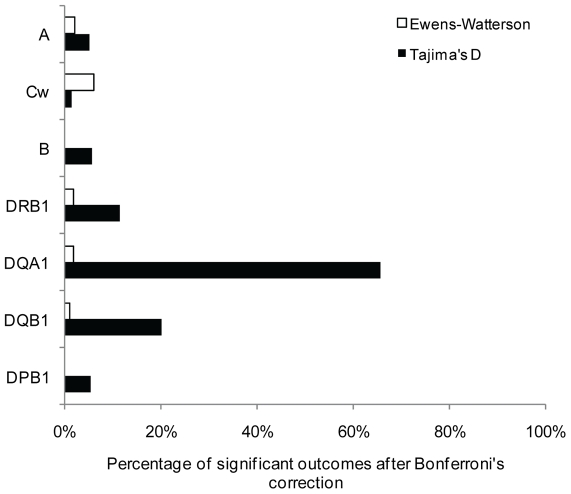
Tests of selective neutrality. Percentages of significant outcomes for Tajima's D statistic and Ewens-Watterson test after Bonferroni's correction, at each locus under study (ordered according to their location on chromosome 6, from telomere to centromere).

The results of the two tests of selective neutrality (Tajima's D and EW) are summarized in Table 2 and described in more details in File S1. Almost all significant outcomes indicated an excess of heterozygotes (i.e. D>0 for Tajima and F_obs_<F_exp_ for EW), suggesting balancing selection as the main cause of the deviation. However, variable proportions of significant tests were found among the different genes (HLA-B>-DQA1>-DRB1>-A>-Cw>-DQB1>-DPB1 for Tajima's D, and HLA-DQA1>-Cw>-DRB1>-B>-DQB1>-A≫-DPB1 for EW). Interestingly, we observed higher proportions of significant outcomes with Tajima's D (64.3 to 96.7%) than with EW (1.8 to 48.3%) for all genes. The discrepancy was even higher after Bonferroni's correction for multiple tests (Figure 3), with 0 to 5.9% of significant outcomes for EW and 1.47 to 65.5% for Tajima's D. Furthermore, for Tajima's D, class II loci, in particular -DQA1 and to a lesser extent -DQB1 and -DRB1, exhibited higher proportions of significant outcomes than HLA-A, -B, -Cw and -DPB1. This pattern can be related to the one described above for nucleotide diversity (π_n_).

**Table 2:** Tajima's D and Ewens-Watterson (EW) tests of selective neutrality at each of the seven HLA loci analyzed.

Tajima					
Locus	n	D<0^a^	%^b^	D>0^a^	%^b^
**A**	97	0 (0)	0 (0)	86 (5)	88.7 (5.2)
**B**	90	0 (0)	0 (0)	87 (5)	96.7 (5.6)
**Cw**	68	0 (0)	0 (0)	57 (1)	83.8 (1.47)
**DRB1**	106	0 (0)	0 (0)	98 (12)	92.5 (11.3)
**DQA1**	58	0 (0)	0 (0)	54 (38)	93.1 (65.5)
**DQB1**	89	1 (0)	1.1 (0)	72 (18)	80.9 (20.2)
**DPB1**	56	2 (1)	3.6 (1.8)	36 (3)	64.3 (5.4)

Ewens-Watterson					
Locus	n	Fobs>Fexp^a^	%^b^	Fobs<Fexp^a^	%^b^
**A**	97	3 (0)	3.1 (0)	15 (2)	15.5 (2.1)
**B**	90	1 (0)	1.1 (0)	21 (0)	23.3 (0)
**Cw**	68	0 (0)	0 (0)	28 (4)	41.2 (5.9)
**DRB1**	106	1 (0)	0.9 (0)	27 (2)	25.5 (1.9)
**DQA1**	58	0 (0)	0 (0)	28 (1)	48.3 (1.7)
**DQB1**	89	0 (0)	0 (0)	19 (1)	21.3 (1.1)
**DPB1**	56	6 (0)	10.7 (0)	1 (0)	1.8 (0)

n: number of populations.

a : number of significant outcomes at the 5% level, and after Bonferroni's correction for multiple tests within brackets.

b : proportion of significant outcomes at the 5% level, and after Bonferroni's correction for multiple tests within brackets.

We also detected a few significant cases showing D<0 with Tajima's test (2 populations at HLA-DPB1, including one which remained significant after Bonferroni's correction, and 1 population at HLA-DQA1), and significant F_obs_>F_exp_ with EW (6 populations at HLA-DPB1, 3 at HLA-A, 1 at HLA-B, and 1 at HLA-DRB1).

### Molecular genetic diversity within and among geographic regions

Φ_ST_ values for populations grouped according to their geographic location are given in Table 3 and File S3. At the worldwide scale, HLA-DPB1 was the locus for which populations were the most differentiated (Φ_ST_ = 14.5%), followed by HLA-DQA1 (13.1%), -DQB1 (8.8%), -A (8.2%), -DRB1 (7.4%), -Cw (6.6%), and -B (6%). When TW, OCE and AME were excluded, HLA-DPB1 was again the gene for which populations were the most differentiated (Φ_ST_ = 8.6%), but this time it was followed by -Cw (4.5%), -B (4.1%), and -A, -DRB1, -DQB1 and -DQA1 (≤3.8%).

**Table 3:** Molecular genetic diversity indexes among populations (Φ_ST_), within geographic groups (Φ_SC_) and among geographic groups (Φ_CT_) and correlation coefficients between genetic and geographic distances (r) in all populations taken together and when excluding TW, OCE and AME, at each HLA locus under study.

	All populations					
Locus	n	Φ_ST_ (%)	r	N	Φ_CT_ (%)	Φ_SC_ (%)
**A**	97 (92)	8.16 ***	0.184 ***	11	3.5 ***	5.07 ***
**B**	90 (85)	5.95 ***	0.167 ***	11	2.6 ***	3.67 ***
**Cw**	68 (63)	6.61 ***	0.226 ***	10	3.89 ***	3.11 ***
**DRB1**	106 (105)	7.43 ***	0.305 ***	9	9.29 ***	6.2 ***
**DQA1**	58	13.13 ***	0.396 ***	10	10.58 ***	4.16 ***
**DQB1**	89	8.84 ***	0.468 ***	10	5.38 ***	4.15 ***
**DPB1**	56	14.49 ***	0.329 ***	11	3.62 ***	4.22 ***

	All populations minus TW, OCE and AME					
Locus	n	Φ_ST_ (%)	r	N	Φ_CT_ (%)	Φ_SC_ (%)
**A**	54	3.84 ***	0.24 ***	6	2.29 ***	2.03 ***
**B**	50	4.08 ***	0.253 ***	6	2.62 ***	1.94 ***
**Cw**	34	4.54 ***	0.326 ***	5	3.19 ***	1.87 ***
**DRB1**	60	3.76 ***	0.431 ***	5	5.63 ***	4.17 ***
**DQA1**	35	3.45 ***	0.277 ***	6	2.3 ***	1.85 ***
**DQB1**	61	3.7 ***	0.32 ***	6	1.3 ***	2.69 ***
**DPB1**	34	8.59 ***	0.464 ***	6	1.84 ***	2.24 ***

n: number of populations; N: number of geographic groups; ***: P<0.001.

In a few cases, because a precise geographic location was not available for some populations, the number of populations studied (n) was different for r calculations, as indicated within brackets. Φ_ST_ and r values for each different geographic group are given in File S3. OCE (Oceania) includes PAC and AUS; AME (the Americas) includes NAM and SAM.

A general tendency emerged for all loci at the regional scale, with NAM, SAM, PAC and TW standing as the most diversified regions in terms of population differentiations (File S3). On the contrary, EUR, NAF and SWA were genetically very homogeneous, with similarly low Φ_ST_ values for all loci (yet significant, except for NAF at HLA-A and -B and for SWA at HLA-DQA1, but these two regions included only 2 populations each). The remaining regions, i.e. SAF, NEA, CSEA and AUS, exhibited intermediate Φ_ST_ values between these two extremes. These Φ_ST_ values were always significant except for NEA at HLA-DQA1 (but this group included only 3 populations).

Overall, Φ_ST_ values were very close to F_ST_s both at the worldwide scale and in the different geographic regions (File S3), yet with generally more marked variations in PAC, AUS, NAM and SAM than in other population groups. Except in two cases where differences can be explained by low sample sizes (non-significant F_ST_ for SWA at HLA-DPB1 and non significant Φ_ST_ for SWA at HLA-DQA1, these groups including 3 and 2 populations, respectively), the significance of these two indexes was also similar.

The results of the hierarchical analyses of molecular genetics variance (AMOVA) are given in Table 3. We found a significant geographic structure (significant Φ_CT_'s) for all loci, but the structure was stronger (Φ_CT_'s>Φ_SC_'s) for HLA-Cw, -DPB1, -DQA1 and -DQB1 at the worldwide scale, and for HLA-A, -B, -Cw, -DPB1 and -DQA1 when TW, OCE and AME were excluded. No significant differences were observed with analyses of genetic variance (ANOVA) based only on allelic frequencies (i.e. similar F_CT_ and F_SC_ values with no changes in significance, results not shown).

### Correlation between genetics and geography

Correlation coefficients between geographic and genetic distances based on the molecular approach were computed both for all populations, and for each geographic region taken separately (Table 3 and File S3). We also estimated these coefficients for all regions minus TW, OCE and AME, because we expected a low correlation with geography in the latter regions due to high population divergence resulting from genetic drift, which may reduce the correlation existing at the global scale. Moreover, correlation coefficients within regions were only computed for regions represented by a minimum of 5 populations in the dataset. This criterion was chosen so as to avoid, on the one hand, a too drastic reduction of the number of regions analyzed, and the computation of meaningless correlation coefficients on the other hand.

Both at the worldwide scale and when excluding TW, OCE and AME, correlation with geography was significant for all genes and reached intermediate to high values (e.g. r = 0.167 for HLA-B and r = 0.468 for HLA-DQB1 worldwide, respectively). However, geography seemed to be a better predictor of the genetic structure when TW, OCE and AME were excluded, as in this case correlation with geography was higher at all loci except HLA-DQA1 and -DQB1. Correlation between genetics and geography was usually lower within regions. This may be the consequence of reducing the sets of populations at this geographic scale, lessening the power of the statistical test, but may also reflect a variable impact of geography among regions in shaping HLA genetic profiles. Yet, except for SEA and NAF (AUS was not tested because of insufficient data), correlation with geography was significant for at least one HLA gene in every geographic region. In SEA, a significant correlation was observed when populations were subdivided into CSEA (at HLA-Cw) and TW (at HLA-B and -Cw), respectively.

The correlation between genetics and geography was also significant at the worldwide scale (both when considering all populations and when excluding TW, OCE and AME) when genetic distances were computed according to the allelic approach (results not shown).

### Differences in genetic distances between the molecular and the allelic approaches

**Figure 4:**
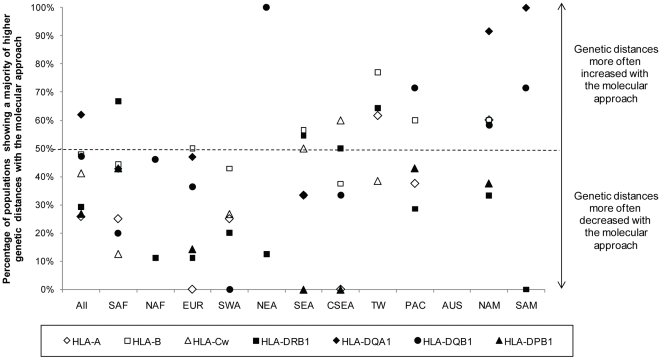
Summary of the comparisons of Reynolds genetic distance matrices between the molecular and allelic approaches. Percentage of populations exhibiting a majority of higher Reynolds genetic distances computed with the molecular approach compared to Reynolds genetic distances computed with the allelic approach (see “Statistical analyses” in Materials and Methods for the definition of the two approaches) within geographic regions, at each locus under study. The detailed comparisons of Reynolds matrices are given in File S4. Percentages were only computed for regions including at least 5 populations (n≥5).

**Figure 5:**
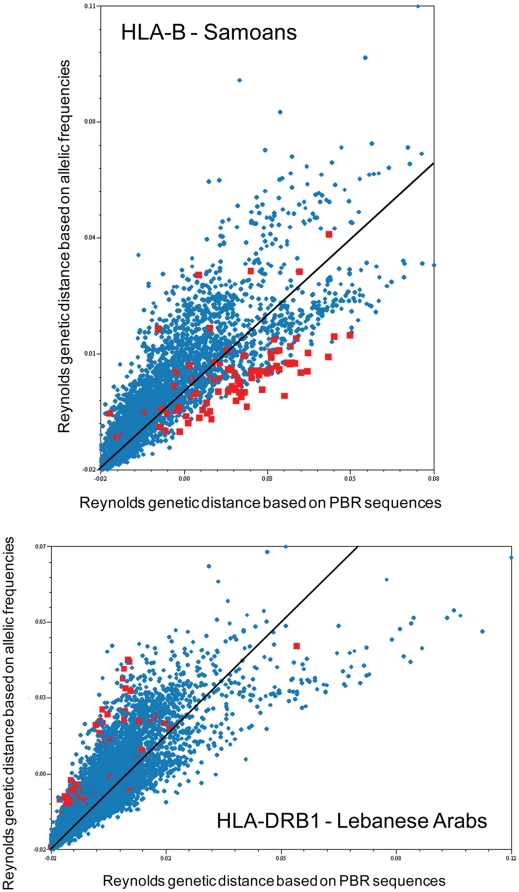
Plot comparisons of Reynolds genetic distance matrices in two populations. Plot comparisons of Reynolds genetic distance matrices computed with either the molecular (X-axis) or the allelic (Y-axis) approach (see “Statistical analyses” in Materials and Methods for the definition of the two approaches) in Samoans for HLA-B (A) and in Lebanese Arabs for HLA-DRB1 (B). The red squares represent the genetic distances between the single out population and the other populations available in the databank at this locus. The blue circles represent the genetic distances between each pair of populations at this locus, excluding the single out population. The black line on the plot indicates the equality of distance between both approaches. The detailed comparisons of Reynolds matrices are given in File S4.

At the regional scale, however, a few differences were observed between the molecular and the allelic approaches. Indeed, correlation coefficients were significant in PAC at HLA-B, SAM at HLA-DQA1, and SWA and EUR at HLA-DRB1 with the molecular but not with the allelic approach, and the reverse situation was observed in SEA at HLA-A, SWA at HLA-B, EUR and NAM at HLA-DQA1, and NAF and SAM at HLA-DQB1. Actually, some populations showed a majority of greater genetic distances to the other populations when using the molecular approach, while others showed a majority of lesser genetic distances (Figure 4 and File S4). Two such examples are given on Figure 5, where genetic distances based on molecular data are plotted against genetic distances based on allele frequencies for two different loci (HLA-B and -DRB1) and where the points corresponding to the distances of two different populations (Samoans and Lebanese Arabs, respectively) are highlighted. For Samoans at HLA-B (Figure 5A), genetic distances were clearly skewed toward higher values with the molecular approach than with the allelic approach, whether the opposite situation was observed for Lebanese Arabs at HLA-DRB1 (Figure 5B). Detailed comparisons of distance matrices obtained with the molecular and allelic approaches (Figure 4 and File S4) showed that HLA-DQA1 was the only locus for which genetic distances were generally underestimated with the allelic approach when all populations were considered together (i.e. column “All” in Figure 4 where more than 60% of the populations exhibit a majority of higher molecular than allelic distances for HLA-DQA1). On the contrary, genetic distances were usually overestimated for HLA-A, -DRB1, and -DPB1 with the allelic approach (i.e. <30% of the populations exhibit a majority of higher molecular than allelic distances). This was also true but to a lesser extent for HLA-Cw (>40%), while HLA-B and -DQB1 were close to equality between the two approaches (about 50%). At the regional scale, a general pattern emerged (Figure 4). In TW, PAC, NAM and SAM, genetics distances between populations tended to be higher at most loci when molecular distances between alleles were considered, while the opposite situation was observed for SAF, NAF, EUR, SWA and CSEA, where genetic distances were commonly overestimated on the sole basis of allelic frequencies.

These distance differences may explain why, in some regions and for some loci (i.e. the ten cases listed above), the molecular and allelic approaches differed in the significance of the correlation with geography.

## Discussion

 In this study, nucleotide diversity in PBR coding exons of HLA genes was extensively analyzed for a dataset of 2,062 sequences in more than 23,500 individuals from about 200 human populations spread worldwide. To our knowledge, this is the first time that nucleotide variability within the MHC is used to analyze human genetic diversity on such a large dataset. Indeed, the information on HLA nucleotide sequences has mostly been studied to discover the evolution of this multigenic family [11], [12], [13], [14], [15], [16], [17], [18], [32], [33], [34], and to understand its functionality [35], [36], [37]. As DNA sequences represent the highest possible level of resolution available for studying the polymorphism of these genes (with amino-acid sequences to whom they are directly related), it should bring more insights and information than the common approach which consists in comparing HLA alleles on the basis of their frequencies, without acknowledging for their molecular divergence. Currently, the availability of databanks containing population samples already screened to reduce typing ambiguities [26], as well as the development of programs allowing for an optimal treatment of ambiguous cases to estimate allele frequencies [27], [28], [29], [30], have allowed to undertake this study, and to include this information with more confidence. Of course, we still had to statistically resolve remaining typing ambiguities in the data, update the allele names to the successive nomenclature changes, and work with missing data in the nucleotide sequences to obtain comparable DNA sequences for all alleles detected in the population data (i.e. formatting the DNA sequences of the alleles not existing anymore in the current sequence alignments following their split into many synonymous variants, with names extending from a 4-digits nomenclature to 6 or more digits). Furthermore, we limited our analyses to the PBR coding exons of HLA genes, and we had to assume the absence of recessive data (commonly known as “blank”) by considering all apparent homozygotes as true homozygotes. However, thanks to the development of molecular typing methods, the presence of blank alleles within HLA data has been greatly reduced (frequencies are generally observed well below 5%) compared to serological typings, where blank could reach frequencies higher than 30% for the HLA-Cw locus [38], [39]. Thus, although it is probable that the above assumption might cause some biases in the results of the present study, we expect them to be minimal.

The first objective of this study was to determine whether the molecular profiles of HLA genetic diversity among human populations (the molecular approach) were congruent or not with those based only on frequency data for the same polymorphism (the allelic approach) [19], [20], [22], [24], [25], [40], [41], [42], [43], [44], [45], [46], [47], [48], [49], [50]. Our results suggest that this is the case at the global scale. Indeed, we find a high and significant correlation between genetics and geography for all HLA genes, both when all populations are considered and when particularly divergent regions are excluded (i.e. TW, OCE and AME). Correlation with geography is lower in these latter regions where populations are more differentiated from each other, in close agreement with the works cited above and our own analyses based on allelic frequencies. Furthermore, a significant geographic structure is observed at all loci, confirming the major role played by geography in shaping HLA molecular variability among populations.

More detailed analyses performed at the regional scale, however, gives fairly different results. When considering different geographic regions and different HLA loci, we observe a total of 10 cases out of 40 (see Results) where taking into account nucleotide information leads to a change of significance (i.e. gain or loss) of the correlation coefficient between genetics and geography. This indicates that the estimation of the genetic distance between some populations closely depends on whether or not the molecular variability of their alleles is considered. For instance, Amerindian, Oceanian and Taiwanese populations are generally more distantly related to other populations if one weights the genetic distance by the molecular distance between their alleles, while the opposite situation is observed for Europeans, continental Asians and Africans (Figure 4). This is indirect evidence that despite the fact that lower numbers of alleles are usually detected in the former group of populations compared to the ones in Europe, Africa and continental Asia, these alleles are highly divergent in the PBR exons, which is probably a result of some functional constraints (see below). On the other hand, due to the complexity of the HLA polymorphism, the changes towards either greater or lesser genetic distances seem hardly predictable for a given population as they vary among loci and also within geographic regions (File S4). As a consequence, to analyze systematically the DNA sequence variation of HLA alleles in population studies may become essential for detailed genetic comparisons among populations.

Our second objective was to investigate the molecular evolution of the HLA polymorphism by comparing the molecular profiles of populations to those depicted on the basis of allelic frequencies. Here, we were mainly interested by the potential effects of evolutionary mechanisms such as balancing selection and gene conversion on the patterns of HLA molecular variability, compared to the influence of demographic factors such as gene flow and genetic drift. While we observe a reduced genetic diversity in TW, OCE and AME (explained by demography) both at the molecular and allelic levels, HLA-Cw exhibits a singular pattern. Indeed, for this locus, mean values of nucleotide diversity are roughly similar in TW, OCE and AME and in EUR, NAF, SAF, SWA, NEA and CSEA. As HLA-Cw is the last HLA gene which was characterized at the DNA level, its nucleotide diversity may currently not be as well described as for the other genes. However, a more likely explanation is that HLA-Cw evolves under specific mechanisms compared to other HLA genes. Actually, HLA-Cw molecules are exposed to fewer pathogenic pressures, in relation to their lower cellular expression than other class I molecules [51]; however, they are also the main KIR ligands and a co-evolution of both polymorphisms is a likely hypothesis [52]. For instance, disease studies and population genetics analyses have reported a significant correlation (either positive or negative) between the frequencies of HLA-Cw alleles and some KIR genes (see [53], [54] and references therein). In addition, HLA-Cw has a more recent origin than other class Ia genes (i.e. classical class I genes), as it is thought that the Cw and B loci shared a common ancestor before the speciation of great apes, but after divergence of apes and Old World monkeys [11], [55]. As a consequence, HLA-Cw had less time to accumulate polymorphism within its DNA sequences than HLA-A and -B, which might be related to our results on nucleotide diversity (Figure 1B) and distributions of pairwise differences between sequences (Figure 2).

Although explainable by demography, the drop of genetic diversity in many aboriginal populations is intriguing in the light of the crucial functions played by HLA genes in the immune surveillance of our organism. Indeed, reduced allele subsets, as those observed in several populations, could affect in some way the overall range of peptide binding specificity. In this respect, relevant results emerge from the mismatch distributions (Figure 2). A majority of alleles are distantly related among each other in all geographic regions and at all loci except HLA-DPB1 and (to a much lesser extent) -DQB1. This pattern may be the consequence of balancing selection in the form of asymmetric heterozygous advantage [56], where individuals carrying distantly related alleles would benefit from a better immunological protection than homozygous ones [57], [58], [59]. Natural selection favoring functionally effective HLA phenotypes would thus explain why populations with reduced HLA genetic diversity may survive, which is in agreement with two previous studies focusing on Amerindians [40], [60]. Of course, our approach is based on the assumption that alleles greatly differing at the DNA level in PBR coding exons cover a larger antigenic repertoire than closely related ones, which has to be confirmed. The fact that HLA-DPB1 exhibits a distinct profile than other classical HLA genes is another support to this hypothesis, as this gene evolves (almost) neutrally [49], [50], [61], [62]. A recent theory of MHC evolution called Associative Balancing Complex (i.e. ABC), where polymorphism in immune genes is maintained both by epistasis among loci and purifying selection against recessive deleterious mutations located nearby MHC loci [63], [64], has been shown (with simulated data) to result in large differentiations between extant alleles. Thus, ABC evolution might also explain the shape of the mismatch distributions observed for classical HLA genes. Furthermore, ABC can act alongside balancing selection, as both evolutionary forces are not mutually exclusive.

Yet, another molecular mechanism may also be involved in the shape of the mismatch distributions observed for HLA genes. Indeed, in addition to DNA substitutions, gene conversion has been proposed as a putative mechanism in the generation of new HLA alleles [57], [65], [66], [67], [68], [69], [70], [71], [72], [73], even though its precise role and molecular process in the evolution of the MHC remain controversial [74]. However, several studies have shown that recombination and gene conversion can affect the shape of a sequence phylogeny and its related mismatch distribution in a similar way as a demographic expansion [75], [76], [77], [78], [79], i.e. resulting in star-like phylogenies and bell-shaped mismatch distributions [80], [81], [82]. Thus, to better understand the possible effect of gene conversion on the molecular profiles observed for HLA genes, we tested for the presence of significant conversion fragments within HLA sequence alignments using the GENECONV program [83], [84] (Table 4). In agreement with other studies [74], [85], [86], HLA-B and HLA-DPB1 stand out as being the most affected by gene conversion as they exhibit the highest number of significant conversion fragments (611 and 99, respectively, after correction for multiple tests by the BLAST method). Other HLA genes exhibit fewer significant conversion fragments after correction (HLA-DRB1>-A>-Cw>-DQA1) or none (HLA-DQB1). Therefore, as HLA-B and -DPB1 are the most prone to gene conversion but display very different mismatch distributions, gene conversion alone does not explain the different patterns observed for those genes. As suggested above, asymmetric overdominant selection and/or ABC evolution must have played a prominent role, even though the effects of recombination, gene conversion and balancing selection are very hard to disentangle from each other and may have acted simultaneously [87].

**Table 4:** Testing for the presence of significant conversion fragments within HLA genes (ordered according to their location on chromosome 6, from telomere to centromere) with the GENECONV program package.

Locus	Number of sequences	Number of nucleotide sites^1^	Number of polymorphic sites^2^	Number of significant conversion fragments (no correction)	Number of significant conversion fragments (BLAST correction)^3^	Number of significant conversion fragments (Bonferroni correction)^4^
**HLA-A**	414	546	157	23444	5	0
**HLA-Cw**	231	546	112	2145	2	0
**HLA-B**	755	546	160	102890	611	0
**HLA-DRB1**	438	270	90	19917	15	0
**HLA-DQA1**	33	249	45	179	1	0
**HLA-DQB1**	70	270	37	164	0	0
**HLA-DPB1**	121	264	43	2252	99	0

Computations are done with 0% of missing data allowed at each nucleotide site, and using all sequences described in the IMGT official database (release 2.13).

1 Exons 2 and 3 for class I genes, exon 2 for class II genes.

2 This represents the number of nucleotide sites used by the program for detecting putative conversion fragments.

3,4 Number of fragments remaining significant after corrections for multiple tests. The BLAST correction is described as more powerful than Bonferroni's correction by the author of GENECONV.

We have shown (Figure 2) and mentioned above that for HLA-DQB1, alleles are more related to each other than for HLA-A, -B, -Cw, -DQA1 and -DRB1, but still much less than for HLA-DPB1. For HLA-DQB1, the result might be related to selective pressures acting on the generation of HLA-DQ heterodimers. Indeed, class II HLA molecules are usually formed by any association of *cis* and *trans* allelic products, except for HLA-DQA1 and -DQB1 where particular allele combinations have been shown to lead to unstable dimers [88], [89]. HLA-DQB1 would have been submitted, then, to some kind of purifying selection due to structural constraints on DQ proteins and slowing down the rate of nucleotide divergence. Further studies are needed to test this hypothesis, as the assumption of co-evolution of HLA-DQA1 and HLA-DQB1 was initially based on serological assignations of their alleles, and has not yet been investigated in relation to DNA sequences.

To complement the results obtained for the mismatch distributions, we tested putative departures from selective neutrality by the Tajima's D statistic and the Ewens-Watterson (EW) test. Evidence for balancing selection is detected at all loci except HLA-DPB1 for EW, but the level of selection appears to be heterogeneous across the HLA region. HLA-B exhibits the highest percentage of significant departures for Tajima's D among the seven loci (which can be related to its mismatch distributions showing very high proportions of unrelated alleles), although we do not detect any significant outcomes in EW after correction for multiple tests. As some studies suggest that this locus is the most selected one [90], [91], we suspect that the apparent neutrality indicated with EW test after correction is due to a lack of power of this test, which is too conservative when the number of alleles is high (HLA-B is by far the gene showing the greatest number of alleles within populations, see File S1) and the sample size not substantially increased [61]. As a matter of fact, the proportions of Tajima's significant outcomes observed at HLA-A, -B and -Cw are also in agreement with a study based on pathogen-driven balancing selection [51]. Among class II genes, HLA-DRB1 exhibits the highest percentage of rejections for Tajima's D, closely followed by HLA-DQA1, whereas HLA-DQB1 exhibits smaller percentages of rejections for both tests, in contradiction with some studies [49], [62], [92], [93]. When taking into account correction for multiple tests for Tajima's D (Figure 3), class II genes, in particular HLA-DQA1, exhibit at least tenfold higher proportions of significant outcomes than class I loci and HLA-DPB1. This might be related to the fact that HLA-DQA1 is the locus with the smallest number of alleles within populations (mean value of 7.4 compared to values of 12.1 to 31.6 at other loci), and that selection acts strongly on maintaining highly divergent sequences at intermediate frequencies as some sort of compensation. Results on EW (see hereafter), nucleotide diversity within populations (Figure 1B) and mismatch distributions (Figure 2) are concordant with this assumption.

Concerning EW, and in agreement with published results [47], [62] of which a recent survey on 497 populations spread worldwide [50], HLA-Cw and -DQA1 show the highest proportions of significant outcomes.

Most of the results are thus congruent with previously published works, but, yet, some contradictions appear between Tajima's D and EW results and at HLA-DQB1 for EW. For the latter discrepancies, the use of unequal datasets is a probable explanation (i.e. there is an agreement on the significant rejects of neutrality observed for HLA-DQB1, it is only the proportion which do vary between our study and previous works and this is clearly dependent on the number of populations analysed), while we should keep in mind that Tajima's D and EW tests do not apply to the same source of variability. As a matter of fact, the former statistic takes into account the nucleotide differences between alleles, while the latter is only based on allelic frequency distributions. Moreover, the outcomes of Tajima's test may be interpreted in different manners, i.e. either by demographic effects or selection. In particular, a significant positive D may result from balancing selection but also from a demographic contraction, while a negative D value may be caused by purifying selection against deleterious alleles or by a demographic expansion. In the seven datasets presently studied, all significant rejects of D are towards positive values, except for 3 cases (2 at HLA-DPB1 and 1 at -DQB1, see Table 2). In populations of small size like Amerindians and Oceanians, a positive D value may be interpreted either by a demographic contraction, and/or by balancing selection, and it is not possible to disentangle the effects caused by both alternatives. At the opposite, most populations included in our analyses (or populations from close geographic areas) have been described as evolving under demographic expansion on the basis of mitochondrial DNA data [76], [79], and thus negative D values were expected under neutral conditions. The fact that D remains significantly positive in most populations from Europe, Africa and continental Asia is a proof that balancing selection is acting significantly on HLA genes and, in the same process, conceals some of the effects of demographic factors.

Our results merely suggest the existence of fluctuating selection across loci, but also over time at the human MHC. According to some authors [94], Tajima's test is more powerful to detect the magnitude of selection acting on HLA genes, as it is better suited to detect ancient traces of selection (history of species timescale) than EW (history of populations timescale). The fact that we observe higher proportions of significant outcomes at every locus according to Tajima's D sustains this assumption. In this context, the singular profile exhibited by HLA-DPB1 can be discussed. Indeed, as already stated, this gene is described as evolving under neutrality, which is concordant with our results of the EW tests. However, we detect a high percentage of significant outcomes according to Tajima's D, including 3 populations remaining significant after correction for multiple tests. Our hypothesis is that HLA-DPB1 presently evolves under neutrality, but retains ancient traces of balancing selection within its nucleotide sequences. The fact that strong evidences for balancing selection at some amino acid sites within the β-1 domain of HLA-DPB1 molecules have been previously identified brings support to this conclusion [92].

In addition to balancing selection, we find evidence for directional selection in some populations. However, most of the populations displaying negative D values and/or excess homozygosity (i.e. F_obs_>F_exp_) are, or have been, prone to genetic drift (see File S1 for details on outcomes for individual populations), suggesting that demography and stochastic forces, rather than directional selection in response to specific pathogens, are the main factors behind these peculiar statistical results. A similar situation was discussed in a previous study [95].

To conclude, this study presents a large scale survey of HLA molecular polymorphism among and within human populations. While our analyses support previous results on the global genetic structure of human populations based on HLA allelic frequencies, we show that taking into account the molecular divergence of HLA DNA sequences leads to different patterns of genetic relationships between populations at regional scales. Notably, populations that are usually described as very distant genetically from each other on the basis of their HLA frequency distributions (e.g. Taiwanese aborigines, Pacific islanders and Amerindians) are even more discriminated when one weights their genetic distances with the nucleotide differences among alleles, while the opposite pattern is observed for the other populations. Furthermore, our investigation reveals new information on the putative mechanisms involved in the evolution of HLA genes. For instance, we confirm that balancing selection left a strong signature on intra-population diversity profiles at most loci and is the main force acting on the maintenance of HLA polymorphism, even though gene conversion and/or recombination may also have some influence. Our analysis of pairwise differences between HLA alleles indicates that balancing selection acting on this system is asymmetric, i.e. stronger for heterozygotes having molecularly distant alleles, even in populations exhibiting a low level of internal diversity. In addition, other kinds of selection (notably at HLA-Cw and -DQB1, which exhibit peculiar profiles compared to other loci) are not excluded. We also detect ancient traces of selection in DNA sequences of the HLA-DPB1 locus, which was assumed to evolve close to neutral conditions on the basis of allelic frequencies distributions. Nonetheless, despite accumulating evidence of natural selection acting on HLA and while some challenging questions remain unanswered (e.g. the intensity and time frame of balancing selection acting on HLA [94]), the history and demography of populations still appear to be the strongest factors lying behind their genetic differentiations. In this context, working with nucleotide sequences from PBR neighboring exons and introns, that may evolve more neutrally, could provide new perspectives.

## Materials and Methods

### Population samples

Population data used in this study are mainly taken from the database of the 12^th^ and 13^th^ International Histocompatibility Workshops (IHWs) [48], [96], completed with additional data from published reports and from our own laboratory (Table 1). A total of 97, 90, 68, 106, 58, 89 and 56 populations were gathered for HLA-A, -B, -Cw, -DRB1, -DQA1, -DQB1 and -DPB1, respectively. We allocated each of these populations to one of ten geographic groups defined *a priori* by the IHWG-AHGD component: North Africa (NAF), sub-Saharan Africa (SAF), Europe (EUR), Southwest Asia (SWA), Northeast Asia (NEA), Southeast Asia (SEA), Pacific (PAC), Australia (AUS), North America (NAM), and South America (SAM); for SEA, we sometimes distinguished the aboriginal populations of Taiwan (TW, not including the Hakka and Minnan populations which are descendent of early Chinese settlers in Taiwan and speak Sino-Tibetan languages, while Taiwanese indigenous peoples speak Austronesian languages) from the continental Southeast Asian populations (CSEA); we also considered an additional category called Other (OTH) for known admixed populations. This last category was not taken into account in most analyses notably because we lack precise geographic information on most populations. Furthermore, we referred to Oceania (OCE) for PAC and AUS taken together, and to America (AME) for NAM and SAM taken together (see File S1 for population names, geographic regions, references, sample sizes and more). Population samples were selected according to two criteria: (1) being characterized at the allelic level (i.e. having all alleles coded on at least 4 digits), and (2) lacking typing uncertainties corresponding to ambiguous allele groups. Indeed, currently used coding techniques for HLA data often do not allow any distinction between groups of alleles that are undistinguishable for a given typing method and alleles that are usually discriminated from each other, except in specific combinations. As a consequence, taking into account ambiguous allele groups often leads to incongruous estimated frequencies (i.e. subdividing the group results in sharing the frequencies equally among all the alleles composing it). On the other hand, samples with individuals showing ambiguities that correspond to multiple possible genotypes were allowed because such ambiguities can easily be treated by the Gene[rate] computer programs. A maximum of 3 to 5 individuals genotyped at the generic level (i.e. 2-digits typing) were allowed in samples of very large size to avoid a drastic reduction in the number of populations included in the analyses. Due to the use of different typing strategies and to the heterogeneity of the data sources, the data included in this study were submitted to automated scripts written in GNU/Linux shell bash to check for the latest updates and changes in the official WHO HLA Nomenclature (e.g. allele renaming, deletion and/or extension from 5 digits to 6 digits adopted in 2002).

While these quality standards were chosen to retain only the best characterized population samples, assuring a solid basis for subsequent statistical analyses, this may have lead to a relative heterogeneity in the datasets used for this study. Drawbacks of this kind are hardly avoidable when data are gathered from various sources and have been characterized in a time span of several years. However, two considerations lessen this problem; first, despite variable numbers of populations from one locus to another, a closer look at the geographic maps provided in File S1 shows that the overall coverage of geographic areas is quite similar for the seven datasets. This is particularly true among class I, and among class II genes, respectively. Indeed, data were principally taken from the 12^th^ and 13^th^ IHWs database, and, thus, as class I and class II genes were mainly genotyped within the scope of the 13^th^ IHW, and 12^th^ IWH, respectively, with some additional typings provided by the 13^th^ IHW, population datasets overlap for HLA-A, -B and -Cw on the one hand, and HLA-DRB1, -DQA1, -DQB1 and -DPB1 on the other hand.

### DNA sequences

A total of 2,062 DNA sequences for alleles of class I (exons 2 and 3) and class II (exon 2) loci were downloaded from the IMGT official database (release 2.13) [10], [97]. This version of the database was chosen so as to be more recent than the genotyping data in hand, thus providing reference DNA sequences for all the alleles detected in the population samples used for this study. Yet, additional sequence formatting was necessary for some of the alleles, as described below. We restricted our analyses to the PBR (Peptide Binding Region) coding exon(s) which are the most polymorphic amongst HLA classical genes and which are screened through most typing protocols (Table 1). As a matter of fact, the PBR exons contain less undetermined positions within sequence alignments than neighboring exons and introns, as alleles have generally been fully sequenced for PBR before being processed to the databank, whereas other DNA regions are often incompletely described and are thus less reliable for sequence comparisons.

Because the population data used in this study were genotyped during a time span of several years and with different typing protocols, we used automated scripts written in GNU/Linux shell bash to process HLA allele sequences at each possible level of resolution (e.g. HLA-A*01, A*0101, A*010101 and A*01010101). Indeed, whereas official sequence alignments include up-to-date allelic characterization, the molecular resolution of alleles in population data may vary (e.g. a population sample may contains typings referring to A*0101 while this allele name is not anymore listed in the sequence alignments following its splits into several 6 and 8 digits alleles, see hereafter). Basically, the scripts replace nucleotides within sequence alignments by a “?” (i.e. an undetermined nucleotide) for any polymorphic position between two (or more) alleles that are to be grouped when converted back to a lower level of resolution (e.g. a C to ? replacement at position 144 of exon 2 for HLA-A*01010101, A*01010102N, A*010102, A*010103, A*010104, A*010106, A*010107 and A*010108, and a G to ? replacement at the same position for HLA-A*010105, respectively, when all these alleles are reduced to the sequence corresponding to HLA-A*0101). This process increases the number of indeterminations within the sequences, thus explaining why only populations characterized at the allelic level (i.e. 4 or more digits) were considered in this work. A maximum level of 5% missing data at each nucleotide site within the sequence alignments was allowed in the estimation of all molecular statistics used in this study, meaning that sites above that threshold were discarded from the analyses (see next section). For the sites below that threshold and containing undetermined nucleotides, a “?” for a given allele was considered as identical to any determined nucleotide (A,G,T,C) at the same site for another allele. Note that the number of nucleotide sites with less than 5% of missing data vary both among different loci as well as for a given locus depending on the number of populations considered in each analysis (e.g. population comparisons at the worldwide scale or on subsets of populations defined on a geographic criterion; analyses at the intra-population level).

### Statistical analyses

All analyses done for this study were performed twice, once using the nucleotide sequences of PBR exons (hereafter the “molecular approach”), and once using only the allelic frequencies (hereafter the “allelic approach”). For the molecular approach, a distance matrix of pairwise differences among DNA sequences was computed and was used to weight all molecular statistics, while all alleles were considered as molecularly equidistant for the allelic approach. We estimated allelic frequencies in each population with an EM algorithm by using the Gene[rate] program allowing to take multiple genotypes (i.e. typing ambiguities) into account [27], [28], [29]. Significant departures from selective neutrality were tested by Tajima's D statistic at the nucleotide level [98] and by Ewens-Watterson's (EW) test on allelic frequency distributions [99], [100]. The distribution of pairwise nucleotide differences between HLA alleles (i.e. mismatch distribution) was obtained for each population, and average mismatch distributions were generated on groups of populations defined on a geographic criterion. The distributions were plotted to depict mean nucleotide divergences between alleles within each population subset (i.e. geographic region). This allowed exploring whether molecular distances between alleles were skewed towards greater values, an expected pattern of asymmetric balancing selection (i.e. heterozygous with highly divergent alleles would be functionally advantaged because they would be able to cover wider spectrums of pathogen's peptides), or not. We estimated HLA molecular genetic diversity at three levels: (1) within populations by computing nucleotide diversity (π_n_) indices [101]; (2) among populations by computing Φ_ST_ fixation indices; (3) using the analysis of molecular variance (AMOVA) hierarchical framework to estimate both the diversity among populations within geographic groups (Φ_SC_ index) and the diversity between geographic groups (Φ_CT_ index) [102]. HLA genetic diversity based on allelic frequencies was estimated at these same levels with the expected heterozygozity (h) [103], F_ST_, F_SC_, and F_CT_ indices [104], respectively. Statistical significance of the fixation indices was tested by the non-parametric permutation procedure implemented in the Arlequin computer package [105], which permutes individual genotypes among populations for Φ_SC_/F_SC_ and Φ_ST_/F_ST_, or populations among groups for Φ_CT_/F_CT_. Coancestry coefficients (i.e. Reynolds genetic distances), which are directly derived from Φ_ST_s or F_ST_s [106], and geographic distances based on the arc length of a sphere and transformed to natural logarithms [107] were computed between all pairs of populations. Correlation coefficients between genetic and geographic distances were computed and tested by a Mantel test [108] using the NTSYS software [109]. Reynolds distance matrixes obtained for the molecular and the allelic approaches were compared using a script written with the R statistical software (http://www.r-project.org/).

## Supporting Information

File S1 Population data and neutrality tests.
(12.21 MB DOC)

File S2 Nucleotide diversity and heterozygosity within populations.
(0.09 MB DOC)

File S3 Genetic diversity among populations and correlation coefficient between genetic and geographic distances in different geographic groups.
(0.23 MB DOC)

File S4 Comparisons of Reynolds genetic distance matrixes.
(0.86 MB DOC)
