# Supplementary material for: HLA DNA Sequence Variation among Human Populations: Molecular Signatures of Demographic and Selective Events
Source: PLoS One. 2011 Feb 1;6(2):e14643. doi: 10.1371/journal.pone.0014643 (PMC3051395; doi:10.1371/journal.pone.0014643)
Supplement: File S1 — Population data and neutrality tests. (12.21 MB DOC) [file pone.0014643.s001.doc]

# Supporting Information S1 - Population data and neutrality tests

List of abbreviations used in below Tables

NAF: North Africa

SAF: sub-Saharan Africa

NAM: North America

SAM: South America

NEA: Northeast Asia

SEA: Southeast Asia

SWA: Southwest Asia

AUS: Australia

EUR: Europe

PAC: Pacific

OTH: Other

N: sample size (individuals)

k: number of sequences detected

F_obs_: observed homozygosity

F_exp_: expected homozygosity

p EW: Ewens-Watterson’s p-value

theta S: θ estimator based on polymorphic sites (S)

theta Π: θ estimator based on the number of pairwise differences

Tajima's D: Tajima’s statistic

p Tajima: Tajima’s p-value

12WS: 12^th^ International Histocompatibility Workshop

θ = 4Nμ in diploid populations, N: sample size and μ: mutation rate.

Significant outcomes at the 5% level are indicated by italic and underlined values.

Significant outcomes after Bonferroni’s correction are indicated in bold.

**HLA-A**

| **Region** | **Population** | **Map #** | **Reference(s)** | **N** | **k** | **F obs** | **F exp** | **p EW** | **theta S** | **theta Π** | **Tajima's D** | **p Tajima** |
| --- | --- | --- | --- | --- | --- | --- | --- | --- | --- | --- | --- | --- |
| NAF | Metalsa | 1 | [1] | 72 | 24 | 0.080 | 0.113 | 0.0934 | 12.086 | 22.053 | 2.575 | *0.99449* |
|  | Chaouya | 2 | [1] | 67 | 29 | 0.077 | 0.087 | 0.4056 | 13.525 | 22.780 | 2.162 | *0.9854* |
| SAF | Zulu | 3 | [1] | 186 | 29 | 0.064 | 0.121 | *0.0034* | 10.778 | 20.843 | 2.709 | *0.99475* |
|  | Cabo Verdeans (Northeast) | 4 | [2] | 62 | 24 | 0.075 | 0.107 | 0.0733 | 12.793 | 21.252 | 2.097 | *0.98445* |
|  | Cabo Verdeans (Southwest) | 5 | [2] | 62 | 27 | 0.070 | 0.092 | 0.1353 | 13.350 | 21.901 | 2.036 | *0.98168* |
|  | Guineans | 6 | [2] | 65 | 20 | 0.087 | 0.136 | *0.0337* | 13.417 | 20.975 | 1.784 | *0.9689* |
|  | Kenyans | 7 | [1] | 143 | 40 | 0.059 | 0.076 | 0.1726 | 11.554 | 21.047 | 2.433 | *0.99067* |
|  | Kenyans (Luo) | 8 | [1,3] | 265 | 31 | 0.060 | 0.123 | *0.0013* | 9.782 | 20.258 | 3.024 | *0.99741* |
|  | Kenyans (Nandi) | 9 | [1,3] | 241 | 29 | 0.073 | 0.129 | *0.0172* | 9.920 | 21.125 | 3.209 | *0.99843* |
|  | Dogons | 10 | [1,3] | 138 | 22 | 0.107 | 0.152 | 0.1431 | 11.137 | 19.799 | 2.304 | *0.98828* |
|  | Ugandans | 11 | [1,3] | 163 | 37 | 0.067 | 0.087 | 0.1896 | 10.687 | 19.400 | 2.383 | *0.98901* |
|  | Mandenka | 12 | [1] | 93 | 25 | 0.083 | 0.118 | 0.1109 | 11.724 | 20.469 | 2.279 | *0.98849* |
|  | Zambians | 13 | [1,3] | 43 | 20 | 0.112 | 0.118 | 0.5308 | 12.734 | 18.037 | 1.365 | 0.93476 |
|  | Shona | 14 | [1] | 225 | 32 | 0.082 | 0.114 | 0.1541 | 10.171 | 19.777 | 2.698 | *0.99379* |
| NAM | Yupik | 15 | [1] | 252 | 19 | 0.379 | 0.205 | *0.9614* | 9.266 | 14.296 | 1.531 | 0.94435 |
|  | Seri | 16 | [1] | 33 | 9 | 0.353 | 0.272 | 0.8415 | 9.455 | 11.966 | 0.879 | 0.85537 |
|  | Mexicans | 17 | [1] | 60 | 26 | 0.126 | 0.096 | 0.8852 | 11.753 | 18.518 | 1.822 | *0.97101* |
|  | Pima | 18 | [1] | 86 | 10 | 0.332 | 0.305 | 0.6794 | 9.787 | 14.712 | 1.529 | 0.94718 |
|  | Amerindians | 19 | [1] | 257 | 29 | 0.130 | 0.131 | 0.5962 | 8.946 | 18.191 | 2.903 | *0.99652* |
| SAM | Brasilians | 20 | [1] | 97 | 27 | 0.097 | 0.108 | 0.4286 | 11.125 | 18.732 | 2.076 | *0.98201* |
|  | Guarani-Kaiowa | 21 | [1] | 143 | 8 | 0.298 | 0.399 | 0.2880 | 6.580 | 12.442 | 2.526 | *0.99202* |
|  | Guarani-Nandewa | 22 | [1] | 52 | 12 | 0.240 | 0.227 | 0.6698 | 9.393 | 14.503 | 1.719 | *0.96442* |
|  | Bari | 23 | [1] | 92 | 5 | 0.420 | 0.529 | 0.3208 | 7.255 | 12.730 | 2.228 | *0.98631* |
| NEA | Koreans | 24 | [1] | 191 | 16 | 0.131 | 0.227 | 0.0532 | 9.507 | 19.100 | 2.897 | *0.99658* |
|  | Buriat | 25 | [1] | 140 | 45 | 0.093 | 0.065 | 0.9442 | 11.755 | 21.058 | 2.350 | *0.98885* |
|  | Tuva | 26 | [1] | 188 | 31 | 0.106 | 0.112 | 0.5378 | 9.223 | 18.928 | 3.018 | *0.99733* |
| SEA | Chinese (Han) #1 | 27 | [1] | 572 | 21 | 0.147 | 0.213 | 0.1898 | 8.400 | 18.807 | 3.329 | *0.99817* |
|  | Chinese | 28 | [1] | 282 | 20 | 0.146 | 0.200 | 0.2461 | 9.115 | 19.177 | 3.090 | *0.99767* |
|  | Chinese (Han) #2 | 29 | [4] | 617 | 82 | 0.096 | 0.049 | *0.9934* | 9.357 | 18.612 | 2.671 | *0.99253* |
|  | Okinawans | 30 | [1] | 105 | 11 | 0.185 | 0.289 | 0.1136 | 8.781 | 19.466 | 3.613 | ***0.99979*** |
|  | Ryukuans | 31 | [1] | 142 | 26 | 0.175 | 0.128 | 0.8884 | 9.479 | 19.846 | 3.199 | *0.99858* |
|  | Malay | 32 | [1] | 124 | 25 | 0.111 | 0.128 | 0.3947 | 10.347 | 19.805 | 2.714 | *0.99557* |
|  | Chinese (Han) #3 | 33 | [1] | 149 | 22 | 0.140 | 0.156 | 0.4841 | 10.203 | 18.358 | 2.342 | *0.98854* |
|  | Singapore (Chinese descent) | 34 | [1] | 86 | 16 | 0.136 | 0.191 | 0.1759 | 10.486 | 19.214 | 2.540 | *0.99383* |
|  | Hakka | 35 | [1] | 55 | 13 | 0.207 | 0.213 | 0.5751 | 10.620 | 17.594 | 2.082 | *0.98313* |
|  | Bunun | 36 | [1] | 101 | 6 | 0.396 | 0.469 | 0.3876 | 7.479 | 16.703 | 3.625 | ***0.99977*** |
|  | Toroko | 37 | [1] | 55 | 6 | 0.291 | 0.434 | 0.1539 | 8.155 | 18.434 | 3.921 | ***0.99997*** |
|  | Ami | 38 | [1] | 98 | 6 | 0.451 | 0.472 | 0.5315 | 7.518 | 15.564 | 3.154 | *0.99859* |
| **Region** | **Population** | **Map #** | **Reference(s)** | **N** | **k** | **F obs** | **F exp** | **p EW** | **theta S** | **theta Π** | **Tajima's D** | **p Tajima** |
| SEA | Paiwan | 39 | [1] | 51 | 6 | 0.749 | 0.428 | *0.9587* | 8.274 | 6.885 | -0.526 | 0.34457 |
|  | Yami (Tao) | 40 | [1] | 50 | 5 | 0.425 | 0.489 | 0.4216 | 7.340 | 14.034 | 2.838 | *0.99781* |
|  | Puyuma | 41 | [1] | 50 | 8 | 0.436 | 0.334 | 0.8286 | 8.499 | 14.656 | 2.281 | *0.99022* |
|  | Pazeh | 42 | [1] | 55 | 10 | 0.217 | 0.277 | 0.3036 | 9.292 | 18.115 | 2.983 | *0.99851* |
|  | Minnan | 43 | [1] | 102 | 15 | 0.166 | 0.213 | 0.2925 | 10.182 | 18.434 | 2.437 | *0.99116* |
|  | Siraya | 44 | [1] | 51 | 11 | 0.271 | 0.247 | 0.7006 | 10.775 | 18.116 | 2.177 | *0.98663* |
|  | Tsou | 45 | [1] | 51 | 5 | 0.631 | 0.490 | 0.7947 | 8.274 | 10.263 | 0.754 | 0.82564 |
|  | Saisiyat | 46 | [1] | 51 | 7 | 0.368 | 0.379 | 0.5633 | 8.274 | 16.047 | 2.946 | *0.99872* |
|  | Atayal | 47 | [1] | 106 | 8 | 0.411 | 0.384 | 0.6604 | 7.250 | 15.068 | 3.150 | *0.99881* |
|  | Rukai | 48 | [1] | 50 | 5 | 0.601 | 0.489 | 0.7615 | 8.305 | 11.749 | 1.303 | 0.92425 |
|  | Thao | 49 | [1] | 30 | 7 | 0.416 | 0.340 | 0.7875 | 9.864 | 15.299 | 1.847 | *0.97532* |
|  | Thaïs | 50 | [1] | 98 | 21 | 0.132 | 0.147 | 0.4604 | 11.277 | 20.115 | 2.380 | *0.99019* |
| SWA | Tamil | 51 | [1] | 50 | 16 | 0.123 | 0.164 | 0.1996 | 12.555 | 20.286 | 1.988 | *0.98023* |
|  | Kurdish | 52 | [1] | 30 | 16 | 0.079 | 0.136 | *0.0009* | 13.939 | 21.137 | 1.765 | *0.97183* |
|  | Svans | 53 | [5] | 80 | 23 | 0.112 | 0.124 | 0.4490 | 10.267 | 17.000 | 2.010 | *0.97983* |
|  | Georgians | 54 | [1] | 105 | 24 | 0.158 | 0.128 | 0.8273 | 10.470 | 17.742 | 2.088 | *0.98178* |
|  | Indians (Golla) | 55 | [1] | 88 | 22 | 0.093 | 0.135 | 0.0994 | 10.792 | 19.248 | 2.391 | *0.99085* |
|  | Nadars | 56 | [6,7] | 61 | 8 | 0.145 | 0.347 | ***0.0000*** | 10.074 | 18.070 | 2.488 | *0.99404* |
|  | Pawra | 57 | [8,9] | 50 | 13 | 0.126 | 0.207 | *0.0402* | 11.975 | 20.293 | 2.237 | *0.98933* |
|  | Marathas | 58 | [10,11] | 91 | 16 | 0.100 | 0.195 | *0.0055* | 9.864 | 19.554 | 2.973 | *0.99815* |
|  | Indians (New Delhi) | 59 | [1] | 66 | 18 | 0.105 | 0.156 | 0.0947 | 10.630 | 17.797 | 2.104 | *0.98375* |
|  | Parsi | 60 | [12,13] | 91 | 25 | 0.087 | 0.118 | 0.1502 | 11.186 | 20.034 | 2.408 | *0.99108* |
|  | Druzes | 61 | [1] | 100 | 26 | 0.069 | 0.115 | *0.0171* | 10.557 | 19.675 | 2.608 | *0.99441* |
|  | Israeli jews | 62 | [1] | 117 | 44 | 0.071 | 0.062 | 0.7969 | 11.276 | 21.017 | 2.589 | *0.99411* |
|  | Omani | 63 | [1] | 119 | 27 | 0.095 | 0.116 | 0.3186 | 10.418 | 18.856 | 2.413 | *0.99068* |
|  | Baloch | 64 | [14] | 63 | 21 | 0.106 | 0.127 | 0.3272 | 11.276 | 17.672 | 1.783 | *0.96828* |
|  | Pathans | 65 | [15] | 36 | 12 | 0.115 | 0.205 | *0.0066* | 11.760 | 16.834 | 1.433 | 0.943 |
|  | Hazara | 66 | [14] | 26 | 16 | 0.133 | 0.128 | 0.6703 | 13.499 | 20.293 | 1.746 | *0.97089* |
|  | Parsi | 67 | [14] | 90 | 22 | 0.084 | 0.135 | *0.0310* | 11.964 | 19.968 | 2.051 | *0.98086* |
|  | Sindhi | 68 | [15] | 39 | 17 | 0.093 | 0.139 | *0.0453* | 12.380 | 17.710 | 1.423 | 0.94035 |
|  | Kalash | 69 | [14] | 60 | 16 | 0.111 | 0.173 | 0.0605 | 10.260 | 16.223 | 1.824 | *0.9709* |
|  | Burusho | 70 | [14] | 92 | 16 | 0.108 | 0.194 | *0.0176* | 10.191 | 17.243 | 2.097 | *0.98167* |
| AUS | Australian aborigines (Kimberley) | 71 | [1] | 36 | 6 | 0.492 | 0.403 | 0.7782 | 8.872 | 11.688 | 1.036 | 0.88501 |
|  | Australian aborigines  (Cape York) | 72 | [1] | 103 | 10 | 0.203 | 0.314 | 0.1289 | 9.487 | 18.740 | 2.917 | *0.99775* |
|  | Australian aborigines  (Groote Eylandt) | 73 | [1] | 75 | 7 | 0.258 | 0.404 | 0.1197 | 9.849 | 18.397 | 2.667 | *0.99608* |
|  | Australian aborigines (Yuendumu) | 74 | [1] | 191 | 10 | 0.302 | 0.349 | 0.4480 | 8.587 | 17.896 | 3.089 | *0.99777* |
|  |  |  |  |  |  |  |  |  |  |  |  |  |
| **Region** | **Population** | **Map #** | **Reference(s)** | **N** | **k** | **F obs** | **F exp** | **p EW** | **theta S** | **theta Π** | **Tajima's D** | **p Tajima** |
| OTH | Brasilians (european and african descent) | 75 | [1] | 97 | 34 | 0.072 | 0.081 | 0.4041 | 11.297 | 19.014 | 2.076 | *0.98165* |
|  | Cubans  (european or african descent) | 76 | [1] | 39 | 23 | 0.082 | 0.094 | 0.3535 | 12.380 | 19.031 | 1.775 | *0.97014* |
|  | Cubans (european descent) | 77 | [1] | 69 | 26 | 0.083 | 0.101 | 0.2647 | 11.453 | 19.378 | 2.161 | *0.98621* |
|  | North Americans  (european descent) | 78 | [1] | 297 | 28 | 0.125 | 0.140 | 0.4591 | 9.047 | 18.453 | 2.901 | *0.9961* |
|  | North Americans  (african descent) | 79 | [1] | 255 | 28 | 0.062 | 0.137 | ***0.0005*** | 9.691 | 19.397 | 2.832 | *0.99616* |
|  | North Americans  (asiatic descent) | 80 | [1] | 411 | 34 | 0.126 | 0.123 | 0.6437 | 9.056 | 19.219 | 3.081 | *0.99732* |
|  | North Americans  (hispanic descent) | 81 | [1] | 247 | 36 | 0.094 | 0.102 | 0.4828 | 9.294 | 18.319 | 2.743 | *0.99461* |
| EUR | Azoreans | 82 | [16] | 231 | 33 | 0.108 | 0.110 | 0.5825 | 9.390 | 18.704 | 2.815 | *0.99568* |
|  | Croatians | 83 | [1] | 150 | 27 | 0.129 | 0.123 | 0.6636 | 9.874 | 18.838 | 2.654 | *0.99455* |
|  | Finnish | 84 | [1] | 90 | 16 | 0.205 | 0.194 | 0.6754 | 10.230 | 15.962 | 1.701 | *0.96069* |
|  | Greeks | 85 | [17] | 85 | 22 | 0.118 | 0.133 | 0.4312 | 10.858 | 18.286 | 2.094 | *0.98267* |
|  | Irish | 86 | [1] | 999 | 26 | 0.152 | 0.191 | 0.355 | 7.582 | 17.942 | 3.557 | *0.99884* |
|  | Portuguese | 87 | [18] | 145 | 27 | 0.111 | 0.123 | 0.468 | 11.368 | 21.088 | 2.527 | *0.99209* |
|  | Czechs | 88 | [1] | 105 | 24 | 0.143 | 0.128 | 0.735 | 10.807 | 18.752 | 2.215 | *0.98665* |
|  | Greek Cypriots | 89 | [17] | 101 | 23 | 0.079 | 0.132 | *0.019* | 10.539 | 19.527 | 2.573 | *0.99428* |
| PAC | Indonesians | 90 | [1] | 50 | 18 | 0.100 | 0.141 | 0.104 | 11.589 | 20.743 | 2.539 | *0.9949* |
|  | Moluccans | 91 | [1] | 25 | 7 | 0.210 | 0.323 | 0.085 | 10.716 | 18.578 | 2.526 | *0.9967* |
|  | East Timorese | 92 | [1] | 57 | 13 | 0.233 | 0.214 | 0.701 | 9.983 | 17.373 | 2.330 | *0.9912* |
|  | Papua New Guinea Lowlanders | 93 | [1] | 79 | 4 | 0.359 | 0.595 | 0.075 | 7.629 | 16.488 | 3.484 | ***0.99964*** |
|  | Papua New Guinea Highlanders | 94 | [1] | 92 | 5 | 0.634 | 0.529 | 0.722 | 7.600 | 10.579 | 1.162 | 0.90207 |
|  | Samoans | 95 | [1] | 50 | 13 | 0.183 | 0.206 | 0.453 | 9.657 | 18.649 | 2.959 | *0.99869* |
|  | Ivatan | 96 | [1] | 50 | 7 | 0.226 | 0.375 | 0.066 | 8.305 | 17.415 | 3.447 | ***0.99986*** |
|  | Filipinos | 97 | [1] | 94 | 16 | 0.162 | 0.195 | 0.368 | 9.121 | 18.725 | 3.161 | *0.99895* |

**HLA-B**

| **Region** | **Population** | **Map #** | **Reference(s)** | **N** | **k** | **F obs** | **F exp** | **p EW** | **theta S** | **theta Π** | **Tajima's D** | **p Tajima** |
| --- | --- | --- | --- | --- | --- | --- | --- | --- | --- | --- | --- | --- |
| NAF | Metalsa | 1 | [1] | 68 | 38 | 0.053 | 0.061 | 0.2987 | 13.671 | 24.034 | 2.393 | *0.99189* |
|  | Chaouya | 2 | [1] | 68 | 43 | 0.040 | 0.051 | 0.0755 | 13.853 | 25.962 | 2.762 | *0.99695* |
| SAF | Zulu | 3 | [1] | 201 | 35 | 0.067 | 0.100 | 0.0672 | 12.324 | 24.601 | 2.900 | *0.99683* |
|  | Kenyans | 4 | [1] | 143 | 47 | 0.049 | 0.062 | 0.1733 | 13.640 | 26.458 | 2.809 | *0.99648* |
|  | Kenyans (Luo) | 5 | [1,3] | 265 | 50 | 0.057 | 0.070 | 0.2470 | 12.410 | 26.028 | 3.146 | *0.99846* |
|  | Kenyans (Nandi) | 6 | [1,3] | 240 | 40 | 0.054 | 0.089 | *0.0158* | 12.593 | 26.445 | 3.173 | *0.99834* |
|  | Dogons | 7 | [1,3] | 138 | 34 | 0.085 | 0.092 | 0.4765 | 11.782 | 21.548 | 2.464 | *0.99149* |
|  | Ugandans | 8 | [1,3] | 161 | 52 | 0.035 | 0.057 | *0.0044* | 12.126 | 24.033 | 2.895 | *0.99682* |
|  | Mandenka | 9 | [1] | 94 | 36 | 0.053 | 0.075 | 0.0551 | 12.734 | 23.884 | 2.685 | *0.99601* |
|  | Zambians | 10 | [1,3] | 44 | 30 | 0.059 | 0.069 | 0.2631 | 14.261 | 24.192 | 2.291 | *0.99135* |
|  | Shona | 11 | [1] | 226 | 40 | 0.058 | 0.087 | *0.0469* | 11.959 | 25.309 | 3.222 | *0.99866* |
| NAM | Yupik | 12 | [1] | 252 | 22 | 0.160 | 0.177 | 0.4899 | 9.561 | 21.057 | 3.399 | *0.99925* |
|  | Seri | 13 | [1] | 33 | 10 | 0.286 | 0.244 | 0.7697 | 12.817 | 16.450 | 0.955 | 0.87256 |
|  | Mexicans | 14 | [1] | 40 | 37 | 0.038 | 0.048 | *0.0447* | 13.729 | 20.645 | 1.669 | *0.96404* |
|  | Pima | 15 | [1] | 89 | 22 | 0.109 | 0.135 | 0.2952 | 11.987 | 22.076 | 2.583 | *0.99451* |
|  | Amerindians | 16 | [1] | 234 | 55 | 0.056 | 0.060 | 0.4783 | 11.153 | 23.720 | 3.232 | *0.99854* |
| SAM | Brasilians | 17 | [1] | 89 | 43 | 0.039 | 0.057 | *0.0101* | 12.508 | 23.702 | 2.753 | *0.99639* |
|  | Guarani-Kaiowa | 18 | [1] | 144 | 34 | 0.082 | 0.094 | 0.3921 | 7.855 | 17.361 | 3.485 | *0.9995* |
|  | Guarani-Nandewa | 19 | [1] | 53 | 32 | 0.077 | 0.069 | 0.7823 | 11.459 | 17.259 | 1.617 | *0.95703* |
|  | Bari | 20 | [1] | 82 | 14 | 0.219 | 0.218 | 0.6188 | 9.165 | 18.440 | 3.072 | *0.99851* |
| NEA | Koreans | 21 | [1] | 200 | 37 | 0.056 | 0.093 | *0.0156* | 12.334 | 25.910 | 3.205 | *0.99865* |
|  | Tuva | 22 | [1] | 180 | 53 | 0.044 | 0.058 | 0.1087 | 13.154 | 25.798 | 2.827 | *0.99605* |
| SEA | Chinese (Han) #1 | 23 | [1] | 572 | 55 | 0.081 | 0.077 | 0.6701 | 10.894 | 24.966 | 3.537 | *0.99895* |
|  | Chinese | 24 | [1] | 282 | 51 | 0.069 | 0.070 | 0.5802 | 11.871 | 25.614 | 3.300 | *0.99871* |
|  | Chinese (Han) #2 | 25 | [4] | 618 | 143 | 0.041 | 0.024 | *0.9940* | 10.912 | 24.661 | 3.439 | *0.99884* |
|  | Okinawans | 26 | [1] | 104 | 20 | 0.090 | 0.157 | *0.0159* | 11.670 | 21.573 | 2.570 | *0.99391* |
|  | Malay | 27 | [1] | 101 | 38 | 0.051 | 0.071 | 0.0552 | 13.938 | 22.276 | 1.833 | *0.97099* |
|  | Chinese (Han) #3 | 28 | [1] | 149 | 35 | 0.085 | 0.091 | 0.4863 | 12.594 | 25.118 | 2.952 | *0.99763* |
|  | Singapore (Chinese descent) | 29 | [1] | 86 | 42 | 0.070 | 0.058 | 0.8443 | 14.331 | 25.263 | 2.370 | *0.99047* |
|  | Hakka | 30 | [1] | 55 | 31 | 0.093 | 0.073 | 0.8901 | 15.171 | 25.847 | 2.274 | *0.98986* |
|  | Bunun | 31 | [1] | 101 | 10 | 0.172 | 0.313 | *0.0346* | 10.199 | 22.171 | 3.533 | ***0.99974*** |
|  | Toroko | 32 | [1] | 55 | 7 | 0.236 | 0.383 | 0.0851 | 10.809 | 17.016 | 1.822 | *0.97133* |
|  | Ami | 33 | [1] | 98 | 8 | 0.230 | 0.379 | 0.0889 | 8.885 | 17.811 | 3.001 | *0.99817* |
|  | Paiwan | 34 | [1] | 51 | 11 | 0.211 | 0.249 | 0.3959 | 12.314 | 19.184 | 1.796 | *0.97005* |
|  | Yami (Tao) | 35 | [1] | 50 | 8 | 0.244 | 0.336 | 0.2297 | 10.623 | 17.185 | 1.975 | *0.97986* |
|  | Puyuma | 36 | [1] | 50 | 11 | 0.127 | 0.246 | *0.0046* | 11.009 | 22.150 | 3.243 | ***0.99952*** |
|  | Pazeh | 37 | [1] | 55 | 22 | 0.103 | 0.115 | 0.4313 | 14.413 | 24.319 | 2.216 | *0.98843* |
|  | Minnan | 38 | [1] | 102 | 32 | 0.093 | 0.090 | 0.6548 | 13.915 | 26.118 | 2.686 | *0.99553* |
|  | Siraya | 39 | [1] | 51 | 25 | 0.092 | 0.094 | 0.5536 | 14.623 | 23.509 | 1.974 | *0.98032* |
| **Region** | **Population** | **Map #** | **Reference(s)** | **N** | **k** | **F obs** | **F exp** | **p EW** | **theta S** | **theta Π** | **Tajima's D** | **p Tajima** |
| SEA | Tsou | 40 | [1] | 51 | 10 | 0.156 | 0.272 | *0.0282* | 10.967 | 21.494 | 3.070 | *0.99923* |
|  | Saisiyat | 41 | [1] | 51 | 9 | 0.403 | 0.302 | 0.8497 | 11.352 | 15.389 | 1.140 | 0.90335 |
|  | Atayal | 42 | [1] | 106 | 10 | 0.212 | 0.318 | 0.1544 | 9.778 | 18.689 | 2.725 | *0.99613* |
|  | Rukai | 43 | [1] | 50 | 9 | 0.165 | 0.301 | *0.0226* | 11.009 | 20.618 | 2.797 | *0.99753* |
|  | Thao | 44 | [1] | 30 | 10 | 0.161 | 0.237 | 0.0960 | 15.226 | 23.353 | 1.832 | *0.97534* |
|  | Thaïs | 45 | [1] | 99 | 33 | 0.067 | 0.085 | 0.2055 | 13.304 | 25.380 | 2.780 | *0.99677* |
| SWA | Tamil | 46 | [1] | 49 | 23 | 0.071 | 0.104 | *0.0417* | 13.768 | 24.044 | 2.426 | *0.99284* |
|  | Svans | 47 | [5] | 80 | 27 | 0.075 | 0.101 | 0.1377 | 11.887 | 20.435 | 2.226 | *0.98742* |
|  | Kurdish | 48 | [1] | 29 | 24 | 0.062 | 0.077 | 0.1227 | 14.906 | 23.351 | 1.950 | *0.98195* |
|  | Georgians | 49 | [1] | 107 | 41 | 0.065 | 0.066 | 0.5583 | 13.803 | 22.819 | 1.993 | *0.97854* |
|  | Indians (Golla) | 50 | [1] | 104 | 33 | 0.075 | 0.087 | 0.3565 | 13.024 | 23.594 | 2.474 | *0.99233* |
|  | Nadars | 51 | [6,7] | 61 | 13 | 0.111 | 0.217 | *0.0033* | 12.832 | 21.604 | 2.171 | *0.98663* |
|  | Pawra | 52 | [8,9] | 50 | 14 | 0.114 | 0.191 | *0.0328* | 12.168 | 23.045 | 2.881 | *0.99819* |
|  | Marathas | 53 | [10,11] | 91 | 23 | 0.079 | 0.128 | *0.0278* | 13.190 | 25.128 | 2.791 | *0.99674* |
|  | Indians (New Delhi) | 54 | [1] | 66 | 31 | 0.067 | 0.079 | 0.3059 | 13.379 | 23.726 | 2.445 | *0.99318* |
|  | Parsi | 55 | [12,13] | 91 | 26 | 0.087 | 0.111 | 0.2190 | 12.967 | 23.782 | 2.566 | *0.99419* |
|  | Bhils | 56 | [9,19] | 50 | 19 | 0.089 | 0.133 | 0.0556 | 13.907 | 22.944 | 2.109 | *0.98537* |
|  | Baloch | 57 | [20] | 100 | 45 | 0.051 | 0.057 | 0.4084 | 13.645 | 23.369 | 2.185 | *0.9855* |
|  | Ashkenazi jews | 58 | [21,22,23,24] | 40 | 20 | 0.102 | 0.115 | 0.3947 | 13.729 | 22.341 | 2.079 | *0.98612* |
|  | Morrocan jews | 59 | [21,22,23,24] | 40 | 21 | 0.084 | 0.108 | 0.1764 | 12.922 | 23.362 | 2.669 | *0.99747* |
|  | Libyan jews | 60 | [21,22,23,24] | 40 | 20 | 0.085 | 0.115 | 0.1127 | 14.133 | 22.439 | 1.950 | *0.97965* |
|  | Druzes | 61 | [1] | 100 | 39 | 0.045 | 0.069 | *0.0099* | 13.622 | 24.372 | 2.418 | *0.99135* |
|  | Israeli jews | 62 | [1] | 109 | 53 | 0.035 | 0.047 | *0.0488* | 13.760 | 25.047 | 2.499 | *0.99303* |
|  | Omani | 63 | [1] | 120 | 38 | 0.075 | 0.076 | 0.5716 | 12.550 | 22.528 | 2.394 | *0.99083* |
|  | Hunza-Burushaski | 64 | [1] | 46 | 21 | 0.119 | 0.113 | 0.6757 | 13.547 | 21.082 | 1.817 | *0.97228* |
|  | Sindhi | 65 | [1] | 39 | 21 | 0.085 | 0.107 | 0.1983 | 13.800 | 23.136 | 2.248 | *0.99046* |
|  | Pathans | 66 | [1] | 38 | 18 | 0.116 | 0.128 | 0.4590 | 13.502 | 21.627 | 2.005 | *0.98282* |
| AUS | Australian aborigines (Kimberley) | 67 | [1] | 38 | 7 | 0.255 | 0.357 | 0.1978 | 11.221 | 20.314 | 2.670 | *0.9969* |
|  | Australian aborigines (Cape York) | 68 | [1] | 100 | 21 | 0.138 | 0.147 | 0.5268 | 12.600 | 23.163 | 2.558 | *0.99423* |
|  | Australian aborigines (Groote Eylandt) | 69 | [1] | 75 | 15 | 0.161 | 0.198 | 0.3428 | 12.177 | 21.511 | 2.386 | *0.99124* |
|  | Australian aborigines (Yuendumu) | 70 | [1] | 193 | 11 | 0.165 | 0.323 | *0.0219* | 9.492 | 20.996 | 3.477 | ***0.99945*** |
| OTH | Brasilians (european or african descent) | 71 | [1] | 69 | 56 | 0.030 | 0.035 | 0.2097 | 14.725 | 24.503 | 2.102 | *0.98397* |
|  | Cubans (european or african descent) | 72 | [1] | 42 | 33 | 0.050 | 0.059 | 0.2009 | 14.194 | 23.627 | 2.195 | *0.98853* |
|  | Cubans  (european descent) | 73 | [1] | 70 | 37 | 0.042 | 0.064 | *0.0053* | 13.780 | 24.485 | 2.448 | *0.99283* |
| **Region** | **Population** | **Map #** | **Reference(s)** | **N** | **k** | **F obs** | **F exp** | **p EW** | **theta S** | **theta Π** | **Tajima's D** | **p Tajima** |
| OTH | North Americans (european descent) | 74 | [1] | 287 | 48 | 0.060 | 0.075 | 0.2470 | 10.247 | 24.188 | 3.837 | ***0.99973*** |
|  | North Americans (african descent) | 75 | [1] | 251 | 49 | 0.048 | 0.071 | 0.0566 | 11.332 | 24.810 | 3.401 | *0.99911* |
|  | North Americans  (asiatic descent) | 76 | [1] | 396 | 66 | 0.044 | 0.056 | 0.1873 | 11.584 | 24.945 | 3.225 | *0.99835* |
|  | North Americans (hispanic descent) | 77 | [1] | 240 | 62 | 0.035 | 0.052 | *0.0201* | 11.556 | 22.588 | 2.740 | *0.99488* |
| EUR | Azoreans | 78 | [16] | 231 | 53 | 0.046 | 0.062 | 0.0931 | 11.916 | 23.742 | 2.860 | *0.99636* |
|  | Greek Cypriots | 79 | [17] | 95 | 35 | 0.062 | 0.078 | 0.1971 | 13.239 | 22.153 | 2.069 | *0.9816* |
|  | Croatians | 80 | [1] | 150 | 44 | 0.058 | 0.068 | 0.2940 | 11.625 | 23.749 | 3.080 | *0.99792* |
|  | Finnish | 81 | [1] | 90 | 25 | 0.084 | 0.116 | 0.1244 | 12.831 | 23.631 | 2.591 | *0.99427* |
|  | Greeks | 82 | [17] | 83 | 32 | 0.062 | 0.083 | 0.1173 | 13.014 | 22.041 | 2.150 | *0.98549* |
|  | Irish | 83 | [1] | 1000 | 49 | 0.091 | 0.099 | 0.5043 | 8.804 | 24.042 | 4.562 | ***0.99994*** |
|  | Portuguese | 84 | [18] | 145 | 43 | 0.061 | 0.070 | 0.3626 | 12.809 | 24.477 | 2.711 | *0.9954* |
|  | Czechs | 85 | [1] | 106 | 36 | 0.046 | 0.078 | *0.0024* | 12.644 | 25.327 | 3.048 | *0.99846* |
| PAC | Indonesians | 86 | [1] | 49 | 30 | 0.063 | 0.072 | 0.2989 | 15.707 | 22.495 | 1.413 | 0.93918 |
|  | Papua New Guinea Highlanders | 87 | [1] | 75 | 14 | 0.154 | 0.214 | 0.1995 | 10.386 | 19.382 | 2.670 | *0.99584* |
|  | Samoans | 88 | [1] | 50 | 25 | 0.130 | 0.094 | 0.9231 | 11.396 | 18.863 | 2.104 | *0.98513* |
|  | Ivatan | 89 | [1] | 50 | 16 | 0.122 | 0.163 | 0.1893 | 12.748 | 19.779 | 1.782 | *0.97022* |
|  | Filipinos | 90 | [1] | 94 | 36 | 0.060 | 0.075 | 0.2025 | 13.939 | 22.615 | 1.918 | *0.97551* |

|  |  |  |  |  |  |  |  |  |  |  |  |  |
| --- | --- | --- | --- | --- | --- | --- | --- | --- | --- | --- | --- | --- |

**HLA-Cw**

| **Region** | **Population** | **Map #** | **Reference(s)** | **N** | **k** | **F obs** | **F exp** | **p EW** | **theta S** | **theta Π** | **Tajima's D** | **p Tajima** |
| --- | --- | --- | --- | --- | --- | --- | --- | --- | --- | --- | --- | --- |
| SAF | Zulu | 1 | [1] | 98 | 23 | 0.096 | 0.132 | 0.1624 | 8.543 | 13.874 | 1.858 | *0.97031* |
|  | Kenyans | 2 | [1] | 112 | 28 | 0.106 | 0.109 | 0.5684 | 8.853 | 13.505 | 1.554 | 0.9481 |
|  | Kenyans (Luo) | 3 | [1,3] | 265 | 24 | 0.097 | 0.163 | *0.0482* | 7.008 | 13.311 | 2.471 | *0.98941* |
|  | Kenyans (Nandi) | 4 | [1,3] | 240 | 24 | 0.112 | 0.158 | 0.1769 | 6.963 | 13.216 | 2.480 | *0.99008* |
|  | Dogons | 5 | [1,3] | 129 | 20 | 0.160 | 0.167 | 0.5555 | 7.343 | 12.391 | 1.983 | *0.9756* |
|  | Ugandans | 6 | [1,3] | 163 | 25 | 0.086 | 0.138 | 0.0502 | 7.858 | 13.342 | 1.993 | *0.97527* |
|  | Zambians | 7 | [1,3] | 45 | 12 | 0.106 | 0.217 | ***0.0007*** | 8.676 | 14.171 | 2.017 | *0.9813* |
|  | Shona | 8 | [1] | 226 | 24 | 0.084 | 0.157 | *0.0120* | 7.026 | 13.866 | 2.700 | *0.9937* |
| NAM | Yupik | 9 | [1] | 149 | 19 | 0.223 | 0.182 | 0.7920 | 7.652 | 13.349 | 2.134 | *0.9817* |
|  | Tarahumara | 10 | [25] | 44 | 10 | 0.241 | 0.264 | 0.4922 | 9.111 | 14.452 | 1.878 | *0.97494* |
|  | Amerindians | 11 | [1] | 248 | 22 | 0.087 | 0.175 | *0.0038* | 7.372 | 14.306 | 2.606 | *0.99304* |
| SAM | Guarani-Kaiowa | 12 | [1] | 143 | 6 | 0.233 | 0.491 | *0.0107* | 5.777 | 14.827 | 4.383 | ***0.99998*** |
|  | Guarani-Nandewa | 13 | [1] | 52 | 8 | 0.192 | 0.339 | *0.0400* | 7.859 | 14.794 | 2.751 | *0.99704* |
|  | Bari | 14 | [1] | 86 | 11 | 0.198 | 0.280 | 0.1994 | 7.515 | 13.843 | 2.506 | *0.99301* |
| NEA | Koreans | 15 | [1] | 200 | 21 | 0.092 | 0.175 | *0.0098* | 7.157 | 13.861 | 2.621 | *0.99337* |
|  | Tuva | 16 | [1] | 174 | 27 | 0.076 | 0.129 | *0.0278* | 8.090 | 15.460 | 2.599 | *0.99315* |
| SEA | Chinese | 17 | [1] | 281 | 19 | 0.120 | 0.208 | *0.0522* | 6.949 | 14.378 | 2.926 | *0.99654* |
|  | Okinawans | 18 | [1] | 105 | 12 | 0.142 | 0.267 | *0.0203* | 7.599 | 13.874 | 2.424 | *0.99106* |
|  | Malay | 19 | [1] | 107 | 21 | 0.096 | 0.151 | 0.0620 | 8.248 | 14.848 | 2.361 | *0.99002* |
|  | Hakka | 20 | [1] | 55 | 15 | 0.145 | 0.182 | 0.2900 | 8.913 | 14.342 | 1.908 | *0.9756* |
|  | Bunun | 21 | [1] | 101 | 8 | 0.189 | 0.380 | *0.0124* | 7.479 | 15.183 | 3.028 | *0.99818* |
|  | Toroko | 22 | [1] | 55 | 7 | 0.218 | 0.383 | *0.0428* | 7.965 | 13.994 | 2.350 | *0.99099* |
|  | Ami | 23 | [1] | 98 | 9 | 0.189 | 0.340 | *0.0402* | 7.860 | 15.258 | 2.785 | *0.99651* |
|  | Paiwan | 24 | [1] | 51 | 9 | 0.312 | 0.303 | 0.6344 | 8.466 | 11.942 | 1.290 | 0.9246 |
|  | Yami (Tao) | 25 | [1] | 50 | 7 | 0.249 | 0.378 | 0.1340 | 7.726 | 13.069 | 2.161 | *0.98625* |
|  | Puyuma | 26 | [1] | 50 | 9 | 0.170 | 0.300 | *0.0309* | 8.305 | 13.816 | 2.085 | *0.98302* |
|  | Pazeh | 27 | [1] | 55 | 11 | 0.127 | 0.253 | *0.0039* | 8.723 | 14.563 | 2.093 | *0.98342* |
|  | Minnan | 28 | [1] | 102 | 16 | 0.132 | 0.199 | 0.1138 | 8.145 | 14.677 | 2.372 | *0.99003* |
|  | Siraya | 29 | [1] | 51 | 14 | 0.120 | 0.191 | 0.0501 | 9.043 | 14.473 | 1.896 | *0.97517* |
|  | Tsou | 30 | [1] | 51 | 9 | 0.205 | 0.300 | 0.1527 | 8.851 | 14.087 | 1.865 | *0.97327* |
|  | Saisiyat | 31 | [1] | 51 | 9 | 0.481 | 0.301 | 0.9229 | 8.658 | 9.959 | 0.473 | 0.74825 |
|  | Atayal | 32 | [1] | 106 | 8 | 0.179 | 0.381 | *0.0063* | 7.250 | 14.407 | 2.884 | *0.99723* |
|  | Rukai | 33 | [1] | 50 | 8 | 0.279 | 0.333 | 0.3883 | 8.499 | 12.100 | 1.334 | 0.92821 |
|  | Thao | 34 | [1] | 30 | 7 | 0.219 | 0.339 | 0.0888 | 8.578 | 14.733 | 2.383 | *0.9938* |
|  | Thaïs #1 | 35 | [1] | 92 | 19 | 0.106 | 0.161 | 0.0842 | 8.118 | 14.223 | 2.241 | *0.98675* |
|  | Thaïs #2 | 36 | [26] | 142 | 17 | 0.084 | 0.202 | ***0.0000*** | 7.390 | 14.264 | 2.667 | *0.99443* |
| SWA | Tamil | 37 | [1] | 48 | 21 | 0.089 | 0.116 | 0.1638 | 9.345 | 13.132 | 1.289 | 0.92313 |
|  | Kurdish | 38 | [1] | 29 | 18 | 0.109 | 0.115 | 0.5065 | 9.721 | 12.962 | 1.121 | 0.90008 |
|  | Georgians | 39 | [1] | 107 | 26 | 0.096 | 0.117 | 0.3030 | 8.416 | 13.016 | 1.615 | *0.95453* |
| **Region** | **Population** | **Map #** | **Reference(s)** | **N** | **k** | **F obs** | **F exp** | **p EW** | **theta S** | **theta Π** | **Tajima's D** | **p Tajima** |
| SWA | Nadars | 40 | [6,7] | 61 | 11 | 0.128 | 0.258 | *0.0038* | 8.555 | 15.013 | 2.336 | *0.99079* |
|  | Pawra | 41 | [8,9] | 50 | 13 | 0.095 | 0.208 | ***0.0000*** | 8.692 | 14.017 | 1.932 | *0.977* |
|  | Marathas | 42 | [10,11] | 91 | 17 | 0.116 | 0.182 | 0.0735 | 7.953 | 14.517 | 2.457 | *0.99219* |
|  | Indians (New Dehli) | 43 | [1] | 56 | 25 | 0.072 | 0.098 | 0.0920 | 8.694 | 12.764 | 1.461 | 0.94274 |
|  | Parsi | 44 | [12,13] | 50 | 23 | 0.099 | 0.105 | 0.5228 | 11.009 | 14.685 | 1.070 | 0.89088 |
|  | Bhils | 45 | [9,19] | 50 | 15 | 0.087 | 0.176 | ***0.0003*** | 9.078 | 14.054 | 1.734 | *0.96575* |
|  | Baloch | 46 | [20] | 100 | 25 | 0.136 | 0.119 | 0.7626 | 8.869 | 14.599 | 1.928 | *0.97416* |
|  | Druzes | 47 | [1] | 100 | 24 | 0.103 | 0.127 | 0.3114 | 8.003 | 12.285 | 1.583 | *0.95003* |
|  | Israeli jews | 48 | [1] | 94 | 42 | 0.049 | 0.061 | 0.1858 | 9.121 | 13.033 | 1.288 | 0.9225 |
|  | Pathans | 49 | [1] | 38 | 15 | 0.108 | 0.161 | 0.0590 | 9.181 | 13.272 | 1.449 | 0.9423 |
|  | Hunza-Burushaski | 50 | [1] | 46 | 16 | 0.160 | 0.160 | 0.6195 | 9.227 | 12.321 | 1.070 | 0.89121 |
|  | Sindhi | 51 | [1] | 39 | 14 | 0.138 | 0.176 | 0.2455 | 8.524 | 12.833 | 1.631 | *0.95853* |
| AUS | Australian aborigines (Kimberley) | 52 | [1] | 28 | 5 | 0.271 | 0.446 | 0.0551 | 7.837 | 14.719 | 2.920 | *0.99898* |
|  | Australian aborigines (Cape York) | 53 | [1] | 89 | 15 | 0.156 | 0.206 | 0.2480 | 8.165 | 13.846 | 2.080 | *0.98117* |
|  | Australian aborigines (Groote Eylandt) | 54 | [1] | 73 | 11 | 0.193 | 0.267 | 0.2123 | 8.277 | 15.322 | 2.587 | *0.99452* |
|  | Australian aborigines (Yuendumu) | 55 | [1] | 192 | 8 | 0.205 | 0.418 | 0.0213 | 6.588 | 14.557 | 3.366 | *0.99911* |
| OTH | Brasilians (european or african descent) | 56 | [1] | 106 | 34 | 0.071 | 0.084 | 0.3090 | 8.598 | 13.730 | 1.768 | *0.96443* |
|  | North Americans (european descent) | 57 | [1] | 292 | 22 | 0.088 | 0.182 | *0.0046* | 6.766 | 13.503 | 2.713 | *0.99367* |
|  | North Americans (african descent) | 58 | [1] | 252 | 23 | 0.091 | 0.167 | *0.0173* | 7.207 | 13.110 | 2.263 | *0.98478* |
|  | North Americans  (asiatic descent) | 59 | [1] | 401 | 24 | 0.099 | 0.176 | *0.0315* | 7.021 | 14.400 | 2.827 | *0.9947* |
|  | North Americans (hispanic descent) | 60 | [1] | 246 | 26 | 0.079 | 0.146 | *0.0115* | 7.380 | 13.468 | 2.287 | *0.98538* |
| EUR | Azoreans | 61 | [16] | 129 | 22 | 0.083 | 0.150 | *0.0114* | 7.511 | 12.848 | 2.054 | *0.97869* |
|  | Finnish | 62 | [1] | 90 | 18 | 0.104 | 0.171 | *0.0426* | 8.669 | 15.291 | 2.292 | *0.98838* |
|  | Irish | 63 | [1] | 1000 | 24 | 0.114 | 0.205 | *0.0416* | 6.114 | 12.856 | 2.812 | *0.99319* |
|  | Czechs | 64 | [1] | 105 | 25 | 0.090 | 0.122 | 0.1666 | 8.105 | 12.938 | 1.759 | *0.96408* |
| PAC | Indonesians | 65 | [1] | 50 | 15 | 0.111 | 0.177 | *0.0428* | 9.271 | 14.922 | 1.931 | *0.97665* |
|  | Samoans | 66 | [1] | 50 | 19 | 0.130 | 0.133 | 0.5759 | 8.885 | 15.080 | 2.203 | *0.98776* |
|  | Ivatan | 67 | [1] | 50 | 10 | 0.166 | 0.272 | 0.0626 | 9.271 | 14.728 | 1.865 | *0.97482* |
|  | Filipinos | 68 | [1] | 94 | 19 | 0.159 | 0.162 | 0.5881 | 8.604 | 14.603 | 2.084 | *0.98199* |
|  | Filipinos | 68 | [1] | 94 | 19 | 0.159 | 0.162 | 0.5881 | 8.604 | 14.603 | 2.084 | *0.98199* |

**HLA-DPB1**

| **Region** | **Population** | **Map #** | **Reference(s)** | **N** | **k** | **F obs** | **F exp** | **p EW** | **theta S** | **theta Π** | **Tajima's D** | **p Tajima** |
| --- | --- | --- | --- | --- | --- | --- | --- | --- | --- | --- | --- | --- |
| SAF | Zulu | 1 | [1] | 87 | 14 | 0.176 | 0.220 | 0.3243 | 3.837 | 8.015 | 3.000 | *0.9978* |
|  | Kenyans | 2 | [1] | 123 | 37 | 0.114 | 0.080 | 0.9310 | 4.276 | 8.647 | 2.786 | *0.99564* |
|  | Merina | 3 | [27,28] | 163 | 15 | 0.136 | 0.235 | *0.0464* | 3.458 | 8.509 | 3.787 | ***0.99969*** |
|  | Aka Pygmies | 4 | [29] | 81 | 13 | 0.498 | 0.233 | *0.9872* | 3.709 | 3.303 | -0.302 | 0.44545 |
|  | Congolese | 5 | [27,29,30] | 84 | 19 | 0.126 | 0.156 | 0.3035 | 4.216 | 8.235 | 2.669 | *0.9943* |
|  | Mandenka | 6 | [31,32] | 93 | 16 | 0.186 | 0.196 | 0.5517 | 3.621 | 8.282 | 3.498 | ***0.99959*** |
|  | Shona | 7 | [1] | 228 | 21 | 0.188 | 0.181 | 0.6497 | 3.732 | 8.030 | 2.959 | *0.99644* |
| NAM | Mixteca Alta | 8 | [33,34] | 103 | 10 | 0.579 | 0.316 | *0.9602* | 3.219 | 2.238 | -0.806 | 0.22805 |
|  | Zapotec | 9 | [33,34] | 91 | 12 | 0.523 | 0.260 | *0.9778* | 4.153 | 3.378 | -0.518 | 0.35089 |
|  | Mixe | 10 | [33,34] | 54 | 5 | 0.826 | 0.496 | *0.9588* | 1.903 | 0.398 | -2.000 | *0.00199* |
|  | Tarahumara | 11 | [25] | 44 | 5 | 0.463 | 0.479 | 0.5483 | 3.169 | 1.908 | -1.133 | 0.12278 |
|  | Pima | 12 | [1] | 95 | 9 | 0.355 | 0.341 | 0.6407 | 3.951 | 3.767 | -0.128 | 0.52125 |
|  | Canoncito | 13 | [1] | 40 | 6 | 0.344 | 0.412 | 0.3833 | 3.836 | 2.889 | -0.730 | 0.25881 |
|  | Sioux | 14 | [1] | 82 | 10 | 0.237 | 0.303 | 0.3152 | 4.054 | 6.126 | 1.426 | 0.93293 |
|  | Zuni | 15 | [1] | 50 | 4 | 0.489 | 0.567 | 0.4025 | 3.477 | 2.441 | -0.849 | 0.21398 |
| SAM | Guarani | 16 | [35,36,37] | 89 | 8 | 0.281 | 0.370 | 0.2866 | 3.304 | 7.622 | 3.515 | ***0.99959*** |
|  | Kaingang | 17 | [35,36,37] | 103 | 7 | 0.380 | 0.422 | 0.4765 | 3.219 | 6.832 | 2.970 | *0.99711* |
|  | Ticuna | 18 | [1] | 49 | 9 | 0.254 | 0.298 | 0.4039 | 3.878 | 7.248 | 2.522 | *0.99336* |
|  | Yanomamo | 19 | [1] | 55 | 7 | 0.504 | 0.382 | 0.8280 | 3.793 | 5.446 | 1.247 | 0.91227 |
| NEA | Japanese (Wajin) | 20 | [38,39] | 70 | 10 | 0.255 | 0.293 | 0.4412 | 3.626 | 6.478 | 2.188 | *0.98403* |
|  | Japanese | 21 | [39,40] | 110 | 13 | 0.247 | 0.250 | 0.5994 | 3.351 | 5.487 | 1.690 | *0.95485* |
|  | Kazakh | 22 | [41] | 38 | 10 | 0.166 | 0.253 | 0.0919 | 4.081 | 6.751 | 1.964 | *0.97707* |
|  | Tuvinians | 23 | [41,42,43] | 191 | 17 | 0.179 | 0.215 | 0.3857 | 3.527 | 6.827 | 2.409 | *0.988* |
| SEA | Uygur | 24 | [39,44] | 62 | 15 | 0.174 | 0.188 | 0.5160 | 4.264 | 7.873 | 2.434 | *0.99119* |
|  | Yao | 25 | [45] | 66 | 10 | 0.327 | 0.289 | 0.7225 | 3.849 | 6.623 | 2.033 | *0.9784* |
|  | Han | 26 | [39,46] | 57 | 12 | 0.192 | 0.232 | 0.3544 | 3.767 | 7.110 | 2.528 | *0.99333* |
|  | Miao | 27 | [45] | 84 | 10 | 0.302 | 0.306 | 0.5818 | 3.685 | 6.358 | 1.992 | *0.97505* |
|  | Hani | 28 | [47] | 47 | 12 | 0.251 | 0.222 | 0.7376 | 4.105 | 7.604 | 2.504 | *0.99344* |
|  | Malay | 29 | [1] | 52 | 20 | 0.129 | 0.127 | 0.6292 | 4.601 | 8.735 | 2.653 | *0.99587* |
| SWA | Svans | 30 | [5] | 80 | 16 | 0.160 | 0.188 | 0.3962 | 4.071 | 7.628 | 2.443 | *0.99098* |
|  | Punjabi | 31 | 12WS | 51 | 20 | 0.216 | 0.126 | *0.9707* | 3.848 | 7.026 | 2.385 | *0.991* |
|  | Lebanese | 32 | [23] | 171 | 22 | 0.192 | 0.161 | 0.7838 | 3.432 | 6.369 | 2.210 | *0.98269* |
| AUS | Australian aborigines (Kimberley) | 33 | [1] | 38 | 5 | 0.497 | 0.471 | 0.6404 | 3.468 | 3.123 | -0.292 | 0.44394 |
| **Region** | **Population** | **Map #** | **Reference(s)** | **N** | **k** | **F obs** | **F exp** | **p EW** | **theta S** | **theta Π** | **Tajima's D** | **p Tajima** |
| AUS | Australian aborigines (Cape York) | 34 | [1] | 96 | 10 | 0.281 | 0.311 | 0.4926 | 3.772 | 4.602 | 0.600 | 0.78024 |
|  | Australian aborigines (Yuendumu) | 35 | [48] | 117 | 9 | 0.313 | 0.354 | 0.4642 | 3.151 | 4.040 | 0.737 | 0.8127 |
| EUR | Belgians | 36 | [49] | 40 | 15 | 0.185 | 0.164 | 0.7500 | 4.644 | 8.168 | 2.302 | *0.9904* |
|  | Bulgarians | 37 | [17,50] | 42 | 9 | 0.232 | 0.288 | 0.3358 | 3.798 | 6.189 | 1.850 | *0.96965* |
|  | Spanish | 38 | [49,51] | 68 | 30 | 0.081 | 0.084 | 0.5460 | 4.739 | 9.199 | 2.722 | *0.99603* |
|  | Finnish | 39 | [1] | 30 | 9 | 0.228 | 0.266 | 0.4068 | 4.289 | 6.927 | 1.908 | *0.97541* |
|  | French | 40 | [49] | 234 | 22 | 0.182 | 0.173 | 0.6714 | 3.272 | 7.725 | 3.421 | *0.99879* |
|  | Greeks | 41 | [17,52] | 96 | 18 | 0.191 | 0.173 | 0.7151 | 4.287 | 6.684 | 1.553 | 0.94604 |
|  | Pomaki | 42 | [17] | 100 | 14 | 0.211 | 0.226 | 0.5305 | 3.916 | 6.078 | 1.509 | 0.94191 |
|  | Italians | 43 | [49] | 99 | 16 | 0.181 | 0.198 | 0.4991 | 3.752 | 7.513 | 2.725 | *0.9948* |
|  | Portuguese #1 | 44 | [49,53] | 108 | 19 | 0.241 | 0.169 | 0.8941 | 3.697 | 6.684 | 2.177 | *0.98299* |
|  | Portuguese #2 | 45 | [49] | 91 | 36 | 0.085 | 0.074 | 0.7948 | 4.499 | 8.583 | 2.547 | *0.99282* |
|  | Czech Gypsies | 46 | [54] | 33 | 13 | 0.206 | 0.181 | 0.7584 | 4.833 | 6.592 | 1.133 | 0.8967 |
|  | Czechs | 47 | [1] | 102 | 20 | 0.227 | 0.157 | 0.9017 | 4.073 | 6.349 | 1.534 | 0.94455 |
|  | Slovenians | 48 | [1] | 100 | 17 | 0.279 | 0.186 | 0.9138 | 3.916 | 6.495 | 1.800 | *0.96448* |
|  | Swiss | 49 | [49,55] | 84 | 17 | 0.179 | 0.180 | 0.6033 | 3.861 | 8.062 | 3.009 | *0.99809* |
| PAC | Moluccans | 50 | [1] | 46 | 12 | 0.189 | 0.220 | 0.4104 | 4.123 | 7.362 | 2.314 | *0.98909* |
|  | East Timorese | 51 | [1] | 86 | 9 | 0.260 | 0.334 | 0.3080 | 3.845 | 7.583 | 2.681 | *0.9949* |
|  | Papua New Guinea Lowlanders | 52 | [1] | 48 | 10 | 0.250 | 0.267 | 0.5226 | 4.089 | 6.627 | 1.819 | *0.96781* |
|  | Papua New Guinea Highlanders | 53 | [1] | 88 | 7 | 0.292 | 0.412 | 0.2129 | 3.655 | 5.605 | 1.457 | 0.93782 |
|  | Maori | 54 | [56] | 199 | 19 | 0.274 | 0.194 | 0.8823 | 3.657 | 5.521 | 1.316 | 0.91738 |
|  | Trobriand Islanders | 55 | [57,58] | 79 | 3 | 0.963 | 0.697 | *0.9664* | 2.484 | 0.327 | -2.242 | ***0.0001*** |
|  | Filipinos | 56 | [1] | 94 | 14 | 0.281 | 0.225 | 0.8130 | 4.130 | 7.110 | 1.996 | *0.97589* |

**HLA-DQA1**

| **Region** | **Population** | **Map #** | **Reference(s)** | **N** | **k** | **F obs** | **F exp** | **p EW** | **theta S** | **theta Π** | **Tajima's D** | **p Tajima** |
| --- | --- | --- | --- | --- | --- | --- | --- | --- | --- | --- | --- | --- |
| NAF | Moroccans | 1 | [1] | 98 | 8 | 0.199 | 0.379 | *0.0275* | 7.689 | 17.749 | 3.864 | ***0.99992*** |
|  | Tunisians | 2 | [59,60] | 97 | 7 | 0.233 | 0.420 | *0.0488* | 7.360 | 17.059 | 3.880 | ***0.99987*** |
|  | Egyptian Copts | 3 | [60] | 40 | 8 | 0.201 | 0.320 | 0.0756 | 8.480 | 18.497 | 3.800 | ***0.99997*** |
| SAF | Zulu | 4 | [1] | 89 | 7 | 0.192 | 0.413 | *0.0069* | 7.818 | 16.969 | 3.487 | ***0.99971*** |
|  | Cameroonese | 5 | [61] | 126 | 11 | 0.196 | 0.302 | 0.1408 | 7.535 | 16.548 | 3.463 | ***0.99945*** |
|  | Banzabi | 6 | [62] | 167 | 7 | 0.313 | 0.450 | 0.2098 | 7.046 | 13.233 | 2.481 | *0.99091* |
|  | Kenyans | 7 | [1] | 119 | 7 | 0.218 | 0.431 | *0.0202* | 7.607 | 16.473 | 3.391 | ***0.99943*** |
|  | Merina | 8 | [27,28] | 163 | 8 | 0.190 | 0.406 | *0.0101* | 6.601 | 14.501 | 3.365 | *0.99872* |
|  | Aka Pygmies | 9 | [29] | 48 | 11 | 0.166 | 0.244 | 0.1307 | 8.761 | 18.066 | 3.364 | ***0.99978*** |
|  | Shona | 10 | [1] | 229 | 8 | 0.211 | 0.425 | *0.0218* | 6.863 | 14.809 | 3.202 | *0.99854* |
| NAM | Yupik | 11 | [1] | 58 | 6 | 0.288 | 0.438 | 0.1391 | 7.697 | 15.860 | 3.268 | ***0.99941*** |
|  | Mexican mestisos | 12 | [33] | 163 | 9 | 0.184 | 0.371 | *0.0185* | 7.073 | 17.675 | 4.243 | ***0.99998*** |
|  | Lacandon | 13 | [1] | 162 | 4 | 0.662 | 0.630 | 0.5815 | 6.450 | 7.739 | 0.561 | 0.776968 |
|  | Seri | 14 | [33] | 84 | 5 | 0.351 | 0.524 | 0.1567 | 7.722 | 12.667 | 1.914 | *0.97347* |
|  | Seri | 15 | [1] | 33 | 4 | 0.361 | 0.541 | 0.1311 | 6.303 | 12.602 | 3.200 | ***0.99978*** |
|  | Mexicans | 16 | [1] | 204 | 7 | 0.206 | 0.460 | *0.0052* | 6.831 | 17.404 | 4.306 | ***0.99994*** |
|  | Mixteca Alta | 17 | [33,34] | 103 | 5 | 0.337 | 0.532 | 0.1175 | 6.946 | 15.139 | 3.439 | ***0.99968*** |
|  | Zapotec | 18 | [33,34] | 82 | 6 | 0.293 | 0.463 | 0.1200 | 7.755 | 14.793 | 2.719 | *0.99594* |
|  | Mixe | 19 | [33,34] | 53 | 5 | 0.401 | 0.493 | 0.3412 | 7.449 | 11.536 | 1.700 | *0.96284* |
|  | Canoncito | 20 | [1] | 40 | 4 | 0.423 | 0.551 | 0.2584 | 7.874 | 7.676 | **-0.081** | 0.541885 |
|  | Sioux | 21 | [1] | 96 | 7 | 0.363 | 0.418 | 0.4332 | 7.716 | 14.054 | 2.430 | *0.99119* |
|  | Zuni | 22 | [1] | 50 | 4 | 0.417 | 0.571 | 0.2147 | 7.919 | 10.507 | 1.023 | 0.880789 |
| SAM | Guarani | 23 | [35,36,37] | 95 | 8 | 0.351 | 0.375 | 0.5247 | 7.400 | 11.454 | 1.618 | *0.953* |
|  | Kaingang | 24 | [35,36,37] | 106 | 5 | 0.373 | 0.533 | 0.2009 | 6.575 | 11.388 | 2.119 | *0.98201* |
|  | Ticuna | 25 | [1] | 49 | 3 | 0.357 | 0.669 | *0.0158* | 5.236 | 12.624 | 4.255 | ***1*** |
|  | Yanomamo | 26 | [1] | 55 | 5 | 0.399 | 0.494 | 0.3346 | 8.534 | 13.314 | 1.749 | *0.96519* |
|  | Yukpa | 27 | [33] | 73 | 3 | 0.625 | 0.690 | 0.4179 | 4.858 | 8.163 | 1.961 | *0.97563* |
| NEA | Kazakh | 28 | [41] | 39 | 8 | 0.195 | 0.318 | 0.0580 | 8.727 | 18.140 | 3.487 | ***0.99991*** |
|  | Halkh | 29 | [39] | 41 | 8 | 0.168 | 0.320 | *0.0104* | 8.237 | 17.441 | 3.578 | ***0.9999*** |
|  | Hoton | 30 | [39] | 84 | 8 | 0.170 | 0.368 | *0.0038* | 7.546 | 16.947 | 3.716 | ***0.99981*** |
|  | Thaïs | 31 | [26] | 142 | 10 | 0.126 | 0.332 | ***0.0000*** | 6.748 | 16.573 | 4.140 | ***0.99996*** |
|  | Uygur | 32 | [39,44] | 66 | 8 | 0.197 | 0.354 | *0.0415* | 8.247 | 17.856 | 3.570 | ***0.99988*** |
|  | Han | 33 | [39,46] | 57 | 8 | 0.182 | 0.344 | *0.0186* | 7.911 | 17.045 | 3.572 | ***0.99984*** |
| SWA | Svans | 34 | [5] | 80 | 9 | 0.184 | 0.329 | *0.0386* | 7.966 | 17.551 | 3.620 | ***0.99979*** |
|  | Punjabi | 35 | 12WS | 51 | 9 | 0.167 | 0.301 | *0.0236* | 8.274 | 18.017 | 3.693 | ***0.99991*** |
|  |  |  |  |  |  |  |  |  |  |  |  |  |
| **Region** | **Population** | **Map #** | **Reference(s)** | **N** | **k** | **F obs** | **F exp** | **p EW** | **theta S** | **theta Π** | **Tajima's D** | **p Tajima** |
| AUS | Australian aborigines (Kimberley) | 36 | [1] | 41 | 5 | 0.234 | 0.476 | *0.0051* | 8.036 | 17.622 | 3.812 | ***0.99998*** |
|  | Australian aborigines (Cape York) | 37 | [1] | 99 | 7 | 0.225 | 0.421 | *0.0295* | 7.164 | 16.185 | 3.693 | ***0.99985*** |
| EUR | Azoreans | 38 | [16] | 129 | 13 | 0.113 | 0.258 | *0.0010* | 7.348 | 18.338 | 4.315 | ***1*** |
|  | Belgians | 39 | [49] | 40 | 7 | 0.164 | 0.363 | *0.0012* | 9.063 | 17.339 | 2.949 | *0.99886* |
|  | Croatians #1 | 40 | [54,63,64] | 106 | 7 | 0.223 | 0.422 | *0.0314* | 7.587 | 17.675 | 3.899 | ***0.99997*** |
|  | Croatians #2 | 41 | [54,65] | 104 | 7 | 0.238 | 0.421 | 0.0528 | 7.611 | 16.058 | 3.260 | ***0.99935*** |
|  | Spanish #1 | 42 | [49,51] | 125 | 11 | 0.138 | 0.300 | *0.0043* | 7.545 | 18.383 | 4.162 | ***0.99999*** |
|  | Spanish #2 | 43 | [49] | 100 | 8 | 0.172 | 0.379 | *0.0043* | 7.662 | 18.257 | 4.076 | ***0.99998*** |
|  | French | 44 | [49,66] | 224 | 8 | 0.166 | 0.422 | *0.0009* | 6.736 | 18.058 | 4.646 | ***0.99999*** |
|  | Greeks | 45 | [17,52] | 96 | 7 | 0.249 | 0.419 | 0.0806 | 7.202 | 17.018 | 4.008 | ***0.99995*** |
|  | Pomaki | 46 | [17] | 100 | 7 | 0.247 | 0.418 | 0.0815 | 7.492 | 16.967 | 3.721 | ***0.99986*** |
|  | Sardinians | 47 | [49,67] | 80 | 9 | 0.226 | 0.331 | 0.1640 | 7.966 | 17.380 | 3.555 | ***0.99979*** |
|  | Italians #1 | 48 | [49] | 99 | 11 | 0.216 | 0.286 | 0.2774 | 7.675 | 17.227 | 3.671 | ***0.99978*** |
|  | Italians #2 | 49 | [49] | 93 | 10 | 0.198 | 0.308 | 0.1211 | 7.931 | 16.703 | 3.288 | ***0.99932*** |
|  | Polish | 50 | [54,68,69] | 99 | 8 | 0.164 | 0.379 | *0.0024* | 7.846 | 17.716 | 3.718 | ***0.99979*** |
|  | Portuguese | 51 | [49] | 111 | 12 | 0.143 | 0.269 | *0.0205* | 7.193 | 18.029 | 4.382 | ***0.99999*** |
|  | Czech Gypsies | 52 | [54] | 33 | 8 | 0.167 | 0.305 | *0.0128* | 9.245 | 17.002 | 2.772 | *0.99829* |
|  | Czechs | 53 | [1] | 105 | 10 | 0.163 | 0.315 | *0.0182* | 7.599 | 18.126 | 4.066 | ***0.99999*** |
|  | Slovenians | 54 | [1] | 100 | 10 | 0.184 | 0.314 | 0.0612 | 7.662 | 17.206 | 3.671 | ***0.99984*** |
| PAC | Moluccans | 55 | [1] | 46 | 7 | 0.348 | 0.371 | 0.5277 | 8.049 | 11.130 | 1.209 | 0.911869 |
|  | East Timorese | 56 | [1] | 86 | 6 | 0.304 | 0.464 | 0.1481 | 6.816 | 10.636 | 1.654 | *0.95693* |
|  | Papua New Guinea Lowlanders | 57 | [1] | 48 | 7 | 0.352 | 0.376 | 0.5186 | 7.982 | 12.293 | 1.699 | *0.96276* |
|  | Papua New Guinea Highlanders | 58 | [1] | 92 | 6 | 0.274 | 0.464 | 0.0814 | 7.255 | 11.993 | 1.928 | *0.97368* |

**HLA-DQB1**

| **Region** | **Population** | **Map #** | **Reference(s)** | **N** | **k** | **F obs** | **F exp** | **p EW** | **theta S** | **theta Π** | **Tajima's D** | **p Tajima** |
| --- | --- | --- | --- | --- | --- | --- | --- | --- | --- | --- | --- | --- |
| NAF | Algerians #1 | 1 | [1] | 99 | 21 | 0.166 | 0.148 | 0.7407 | 9.040 | 18.706 | 3.196 | *0.99894* |
|  | Algerians #2 | 2 | [60,70] | 100 | 16 | 0.179 | 0.198 | 0.4881 | 8.513 | 18.071 | 3.338 | *0.99931* |
|  | Algerians #3 | 3 | [71] | 106 | 12 | 0.137 | 0.268 | *0.0117* | 6.570 | 14.102 | 3.317 | *0.99924* |
|  | Mozabites | 4 | [60] | 107 | 12 | 0.168 | 0.268 | 0.0976 | 8.416 | 19.421 | 3.865 | ***0.99994*** |
|  | Egyptians #1 | 5 | [60,72] | 101 | 22 | 0.101 | 0.141 | 0.1514 | 4.420 | 9.079 | 2.928 | *0.99742* |
|  | Egyptians #2 | 6 | [60,72] | 94 | 22 | 0.094 | 0.137 | 0.0944 | 6.195 | 12.354 | 2.886 | *0.99743* |
|  | Bedouins | 7 | [60,72,73] | 98 | 19 | 0.099 | 0.165 | *0.0344* | 8.714 | 18.375 | 3.307 | *0.99938* |
|  | Moroccans | 8 | [1] | 98 | 14 | 0.215 | 0.227 | 0.5446 | 8.543 | 18.247 | 3.383 | ***0.9995*** |
|  | Tunisians #1 | 9 | [74] | 96 | 11 | 0.219 | 0.285 | 0.3026 | 8.573 | 17.731 | 3.187 | *0.99911* |
|  | Jerba berbers | 10 | [74] | 55 | 10 | 0.182 | 0.278 | 0.1158 | 9.292 | 19.274 | 3.375 | ***0.99975*** |
|  | Matmata berbers | 11 | [74] | 81 | 12 | 0.172 | 0.251 | 0.1556 | 8.655 | 19.401 | 3.756 | ***0.99989*** |
|  | Tunisians #2 | 12 | [75] | 104 | 17 | 0.120 | 0.188 | 0.0830 | 8.464 | 17.928 | 3.315 | *0.99929* |
|  | Egyptian Copts | 13 | [60] | 40 | 10 | 0.153 | 0.256 | *0.0361* | 7.874 | 13.929 | 2.460 | *0.99386* |
| SAF | Zulu | 14 | [1] | 87 | 9 | 0.163 | 0.336 | *0.0078* | 5.930 | 13.550 | 3.736 | *0.99988* |
|  | Cameroonese | 15 | [61] | 126 | 18 | 0.150 | 0.185 | 0.3378 | 8.518 | 17.670 | 3.142 | *0.99857* |
|  | Oromo | 16 | [27,76] | 83 | 10 | 0.168 | 0.304 | *0.0320* | 5.628 | 12.821 | 3.709 | ***0.99989*** |
|  | Amhara | 17 | [27,76] | 98 | 11 | 0.163 | 0.289 | *0.0412* | 5.467 | 12.778 | 3.819 | ***0.99987*** |
|  | Banzabi | 18 | [62] | 167 | 11 | 0.248 | 0.314 | 0.3386 | 7.672 | 15.834 | 3.029 | *0.99789* |
|  | Kenyans | 19 | [1] | 113 | 15 | 0.146 | 0.219 | 0.1371 | 8.840 | 21.099 | 4.099 | ***0.99999*** |
|  | Merina | 20 | [27,28] | 163 | 12 | 0.224 | 0.291 | 0.3133 | 6.130 | 12.014 | 2.680 | *0.9943* |
|  | Congolese | 21 | [27,29,30] | 88 | 12 | 0.192 | 0.257 | 0.2562 | 8.712 | 18.785 | 3.478 | ***0.99973*** |
|  | Rwandans | 22 | [1] | 280 | 15 | 0.158 | 0.262 | 0.0908 | 7.242 | 17.879 | 4.036 | ***0.99991*** |
|  | Shona | 23 | [1] | 229 | 15 | 0.163 | 0.251 | 0.1363 | 7.459 | 18.450 | 4.105 | ***0.99996*** |
| NAM | Yupik | 24 | [1] | 251 | 12 | 0.423 | 0.311 | 0.8459 | 7.506 | 9.054 | 0.572 | 0.78155 |
|  | Mexican mestisos | 25 | [33] | 163 | 16 | 0.171 | 0.222 | 0.3023 | 7.858 | 15.633 | 2.826 | *0.99603* |
|  | Lacandon | 26 | [1] | 162 | 5 | 0.643 | 0.558 | 0.6920 | 7.394 | 2.923 | **-1.719** | 0.01031 |
|  | Seri | 27 | [33] | 84 | 5 | 0.348 | 0.524 | 0.1525 | 8.248 | 11.249 | 1.093 | 0.8932 |
|  | Seri | 28 | [1] | 33 | 4 | 0.361 | 0.542 | 0.1291 | 7.144 | 10.439 | 1.494 | 0.94596 |
|  | Mexicans | 29 | [1] | 204 | 13 | 0.179 | 0.279 | 0.1315 | 7.590 | 15.277 | 2.845 | *0.99604* |
|  | Mixe | 30 | [33,34] | 55 | 5 | 0.327 | 0.496 | 0.1282 | 7.206 | 10.201 | 1.279 | 0.92013 |
|  | Zapotec | 31 | [33,34] | 78 | 11 | 0.229 | 0.273 | 0.3999 | 8.535 | 10.700 | 0.769 | 0.8302 |
|  | Mixteca Alta | 32 | [33,34] | 103 | 10 | 0.260 | 0.316 | 0.3810 | 4.235 | 6.112 | 1.221 | 0.90867 |
|  | Canoncito | 33 | [1] | 40 | 6 | 0.421 | 0.413 | 0.6062 | 5.249 | 10.631 | 3.155 | *0.99929* |
|  | Sioux | 34 | [1] | 95 | 9 | 0.294 | 0.342 | 0.4371 | 8.589 | 9.764 | 0.409 | 0.73068 |
|  | Zuni | 35 | [1] | 50 | 6 | 0.384 | 0.425 | 0.4751 | 9.078 | 10.799 | 0.600 | 0.78746 |
| SAM | Guarani-Kaiowa | 36 | [1] | 144 | 4 | 0.381 | 0.628 | 0.0902 | 3.527 | 7.550 | 2.992 | *0.99744* |
|  | Guarani-Nandewa | 37 | [1] | 53 | 6 | 0.465 | 0.430 | 0.6651 | 8.976 | 9.004 | 0.010 | 0.58118 |
|  |  |  |  |  |  |  |  |  |  |  |  |  |
| **Region** | **Population** | **Map #** | **Reference(s)** | **N** | **k** | **F obs** | **F exp** | **p EW** | **theta S** | **theta Π** | **Tajima's D** | **p Tajima** |
| SAM | Guarani | 38 | [35,36,37] | 79 | 8 | 0.375 | 0.364 | 0.6270 | 8.703 | 7.928 | **-0.270** | 0.46504 |
|  | Kaingang | 39 | [35,36,37] | 88 | 5 | 0.371 | 0.525 | 0.2089 | 6.615 | 11.259 | 2.062 | *0.98031* |
|  | Ticuna | 40 | [1] | 49 | 5 | 0.303 | 0.488 | 0.0877 | 6.593 | 9.918 | 1.557 | 0.94947 |
|  | Yanomamo | 41 | [1] | 55 | 3 | 0.449 | 0.672 | 0.1092 | 4.551 | 5.168 | 0.397 | 0.71884 |
|  | Yukpa | 42 | [33] | 70 | 4 | 0.629 | 0.589 | 0.6115 | 4.170 | 4.946 | 0.528 | 0.7593 |
| NEA | Japanese (Wajin) | 43 | [38,39] | 76 | 11 | 0.111 | 0.272 | ***0.0002*** | 5.538 | 11.502 | 3.142 | *0.99891* |
|  | Japanese | 44 | [39,40] | 110 | 15 | 0.100 | 0.217 | *0.0009* | 5.194 | 11.170 | 3.239 | *0.99868* |
|  | Kazakh | 45 | [41] | 39 | 14 | 0.135 | 0.176 | 0.2213 | 8.118 | 14.561 | 2.551 | *0.99572* |
|  | Halkh | 46 | [39] | 41 | 16 | 0.141 | 0.153 | 0.4973 | 10.647 | 18.075 | 2.274 | *0.99109* |
|  | Hoton | 47 | [39] | 84 | 11 | 0.192 | 0.278 | 0.1820 | 9.477 | 19.932 | 3.350 | ***0.99959*** |
| SEA | Uygur | 48 | [39,44] | 66 | 13 | 0.156 | 0.223 | 0.1702 | 9.164 | 17.772 | 2.902 | *0.99819* |
|  | Han | 49 | [39,46] | 57 | 14 | 0.108 | 0.199 | *0.0096* | 7.534 | 14.257 | 2.749 | *0.99695* |
|  | Malay | 50 | [1] | 55 | 12 | 0.181 | 0.231 | 0.2812 | 6.637 | 10.546 | 1.800 | *0.96863* |
|  | Thaïs | 51 | [26] | 142 | 14 | 0.117 | 0.246 | *0.0046* | 6.266 | 12.846 | 2.965 | *0.99735* |
|  | Kinh | 52 | [1] | 100 | 14 | 0.196 | 0.228 | 0.4266 | 9.195 | 15.076 | 1.913 | *0.97408* |
|  | Muong | 53 | [1] | 82 | 19 | 0.269 | 0.155 | 0.9635 | 9.517 | 16.275 | 2.161 | *0.98501* |
| SWA | Bahrainis | 54 | [77] | 72 | 7 | 0.205 | 0.401 | 0.0169 | 9.741 | 21.841 | 3.826 | ***0.99991*** |
|  | Svans | 55 | [5] | 80 | 10 | 0.147 | 0.299 | *0.0073* | 8.674 | 18.452 | 3.414 | *0.99958* |
|  | Punjabi | 56 | 12WS | 42 | 12 | 0.117 | 0.215 | *0.0062* | 4.998 | 7.660 | 1.622 | *0.95428* |
|  | Lebanese | 57 | [78] | 191 | 7 | 0.242 | 0.455 | *0.0403* | 8.281 | 18.680 | 3.568 | ***0.99953*** |
|  | Turks | 58 | [1] | 245 | 19 | 0.132 | 0.203 | 0.1219 | 3.692 | 8.617 | 3.407 | *0.99902* |
| AUS | Australian aborigines (Kimberley) | 59 | [1] | 41 | 6 | 0.318 | 0.414 | 0.2793 | 3.817 | 5.914 | 1.620 | *0.95418* |
|  | Australian aborigines (Cape York) | 60 | [1] | 99 | 12 | 0.180 | 0.263 | 0.1607 | 4.605 | 7.900 | 2.000 | *0.97584* |
| EUR | Azoreans | 61 | [16] | 129 | 16 | 0.114 | 0.210 | *0.0210* | 8.159 | 18.087 | 3.541 | *0.99941* |
|  | Belgians | 62 | [49] | 40 | 14 | 0.116 | 0.178 | 0.0608 | 9.691 | 15.887 | 2.076 | *0.985* |
|  | Bulgarians | 63 | [17,50] | 120 | 13 | 0.151 | 0.258 | 0.0584 | 5.614 | 11.571 | 2.995 | *0.99774* |
|  | Croatians #1 | 64 | [54,63,64] | 104 | 17 | 0.141 | 0.187 | 0.2437 | 8.626 | 16.826 | 2.821 | *0.99684* |
|  | Croatians #2 | 65 | [54,65] | 102 | 14 | 0.161 | 0.229 | 0.1848 | 6.109 | 11.133 | 2.370 | *0.98961* |
|  | Croatians #3 | 66 | [54,79] | 139 | 15 | 0.133 | 0.228 | 0.0503 | 4.191 | 8.684 | 2.889 | *0.99625* |
|  | Spanish | 67 | [49] | 100 | 15 | 0.124 | 0.212 | *0.0364* | 8.513 | 17.942 | 3.293 | *0.9993* |
|  | Spanish Basques | 68 | [49,80] | 158 | 15 | 0.253 | 0.234 | 0.6939 | 7.897 | 17.758 | 3.576 | ***0.99953*** |
|  | French #1 | 69 | [49,66] | 224 | 13 | 0.141 | 0.285 | 0.0168 | 7.484 | 17.578 | 3.764 | ***0.99961*** |
|  | French #2 | 70 | [49] | 234 | 16 | 0.137 | 0.237 | 0.0549 | 5.800 | 12.274 | 3.031 | *0.99696* |
|  | Greeks | 71 | [17,52] | 96 | 13 | 0.177 | 0.244 | 0.2187 | 6.687 | 11.676 | 2.179 | *0.98476* |
|  | Pomaki | 72 | [17] | 100 | 14 | 0.184 | 0.229 | 0.3464 | 7.832 | 17.165 | 3.519 | ***0.99976*** |
|  | Sardinians | 73 | [49,67] | 80 | 13 | 0.168 | 0.233 | 0.2041 | 8.674 | 18.213 | 3.330 | *0.99938* |
|  | Italians #1 | 74 | [49] | 98 | 13 | 0.152 | 0.244 | 0.0832 | 4.271 | 9.148 | 3.163 | *0.9986* |
| **Region** | **Population** | **Map #** | **Reference(s)** | **N** | **k** | **F obs** | **F exp** | **p EW** | **theta S** | **theta Π** | **Tajima's D** | **p Tajima** |
| EUR | Italians #2 | 75 | [49] | 93 | 12 | 0.158 | 0.261 | 0.0706 | 4.138 | 9.038 | 3.280 | *0.99898* |
|  | Polish | 76 | [54,68,69] | 99 | 14 | 0.130 | 0.226 | *0.0329* | 8.528 | 18.023 | 3.313 | *0.99931* |
|  | Portuguese #1 | 77 | [49,53] | 220 | 17 | 0.149 | 0.222 | 0.1563 | 7.654 | 17.969 | 3.772 | ***0.99981*** |
|  | Portuguese #2 | 78 | [49] | 111 | 18 | 0.120 | 0.180 | 0.1189 | 6.524 | 13.338 | 3.010 | *0.9979* |
|  | Czechs #1 | 79 | [54] | 35 | 12 | 0.151 | 0.204 | 0.1832 | 5.396 | 9.848 | 2.584 | *0.9957* |
|  | Czech Gypsies | 80 | [54] | 33 | 10 | 0.151 | 0.243 | *0.0426* | 4.833 | 7.904 | 1.978 | *0.97961* |
|  | Czechs #2 | 81 | [1] | 106 | 15 | 0.127 | 0.214 | *0.0471* | 8.430 | 17.612 | 3.222 | *0.99901* |
|  | Slovenians | 82 | [1] | 100 | 15 | 0.121 | 0.212 | *0.0270* | 8.173 | 17.872 | 3.517 | ***0.99966*** |
| PAC | Moluccans | 83 | [1] | 46 | 9 | 0.235 | 0.296 | 0.3163 | 7.460 | 11.152 | 1.554 | *0.95101* |
|  | East Timorese | 84 | [1] | 86 | 10 | 0.184 | 0.306 | 0.0756 | 4.369 | 6.382 | 1.293 | 0.91626 |
|  | Papua New Guinea Lowlanders #1 | 85 | [1] | 48 | 10 | 0.215 | 0.268 | 0.3227 | 7.788 | 11.593 | 1.534 | 0.94922 |
|  | Papua New Guinea Lowlanders #2 | 86 | [1] | 91 | 9 | 0.419 | 0.340 | 0.7921 | 5.711 | 8.985 | 1.655 | *0.95478* |
|  | Papua New Guinea Highlanders | 87 | [1] | 91 | 10 | 0.181 | 0.308 | 0.0648 | 4.499 | 6.542 | 1.274 | 0.91294 |
|  | Maori | 88 | [56] | 187 | 18 | 0.138 | 0.203 | 0.1599 | 8.927 | 17.907 | 2.879 | *0.99614* |
|  | Filipinos | 89 | [1] | 94 | 14 | 0.157 | 0.224 | 0.1821 | 6.884 | 11.945 | 2.157 | *0.98412* |

**HLA-DRB1**

| **Region** | **Population** | **Map #** | **Reference(s)** | **N** | **k** | **F obs** | **F exp** | **p EW** | **theta S** | **theta Π** | **Tajima's D** | **p Tajima** |
| --- | --- | --- | --- | --- | --- | --- | --- | --- | --- | --- | --- | --- |
| NAF | Algerians | 1 | [1] | 99 | 33 | 0.064 | 0.085 | 0.1290 | 8.869 | 18.279 | 3.166 | *0.99888* |
|  | Moroccans #1 | 2 | [1] | 91 | 27 | 0.071 | 0.106 | *0.0469* | 9.172 | 18.185 | 2.959 | *0.99814* |
|  | Metalsa | 3 | [1] | 99 | 28 | 0.094 | 0.104 | 0.4518 | 9.551 | 19.342 | 3.076 | *0.99851* |
|  | Chaouya | 4 | [1] | 99 | 29 | 0.078 | 0.100 | 0.2077 | 9.551 | 20.216 | 3.350 | *0.99952* |
|  | Moroccans #2 | 5 | [1] | 98 | 21 | 0.109 | 0.147 | 0.1959 | 8.714 | 18.978 | 3.513 | ***0.99969*** |
|  | Jerba berbers | 6 | [74] | 55 | 19 | 0.070 | 0.137 | ***0.0003*** | 9.672 | 17.992 | 2.710 | *0.99698* |
|  | Matmata berbers | 7 | [74] | 81 | 26 | 0.088 | 0.108 | 0.2882 | 9.008 | 18.658 | 3.250 | *0.99927* |
|  | Tunisians | 8 | [75] | 104 | 36 | 0.068 | 0.077 | 0.3631 | 8.979 | 17.375 | 2.785 | *0.99645* |
|  | Egyptian Copts | 9 | [60] | 40 | 20 | 0.080 | 0.115 | *0.0461* | 10.297 | 18.032 | 2.449 | *0.99433* |
| SAF | Zulu | 10 | [1] | 88 | 24 | 0.116 | 0.121 | 0.5492 | 9.226 | 15.573 | 2.078 | *0.98097* |
|  | Cameroonese | 11 | [61] | 126 | 18 | 0.131 | 0.185 | 0.1736 | 9.173 | 18.182 | 2.888 | *0.99719* |
|  | Cabo Verdeans (Northeast) | 12 | [2] | 62 | 28 | 0.059 | 0.088 | *0.0199* | 10.568 | 18.790 | 2.439 | *0.99257* |
|  | Cabo Verdeans (Southwest) | 13 | [2] | 62 | 25 | 0.066 | 0.102 | *0.0219* | 10.383 | 19.641 | 2.793 | *0.99734* |
|  | Guineans | 14 | [2] | 65 | 23 | 0.093 | 0.115 | 0.2600 | 10.491 | 18.171 | 2.287 | *0.9896* |
|  | Merina | 15 | [27,28] | 163 | 26 | 0.125 | 0.133 | 0.5218 | 8.330 | 17.383 | 3.120 | *0.99828* |
|  | Dogon | 16 | [1] | 138 | 21 | 0.099 | 0.160 | 0.0550 | 9.523 | 16.367 | 2.107 | *0.98125* |
|  | Aka Pygmies | 17 | [29] | 93 | 18 | 0.139 | 0.173 | 0.3143 | 9.655 | 20.373 | 3.349 | *0.99946* |
|  | Shona | 18 | [1] | 229 | 28 | 0.081 | 0.133 | *0.0389* | 7.758 | 16.191 | 3.039 | *0.99719* |
| NAM | Yupik | 19 | [1] | 252 | 21 | 0.148 | 0.185 | 0.3351 | 7.501 | 14.042 | 2.418 | *0.98956* |
|  | Lacandon | 20 | [1] | 162 | 11 | 0.356 | 0.313 | 0.7239 | 6.607 | 8.114 | 0.641 | 0.79757 |
|  | Seri #1 | 21 | [33] | 83 | 6 | 0.347 | 0.458 | 0.2890 | 7.386 | 11.164 | 1.524 | 0.94558 |
|  | Seri #2 | 22 | [1] | 33 | 6 | 0.342 | 0.396 | 0.4143 | 8.615 | 10.663 | 0.782 | 0.83199 |
|  | Mexicans | 23 | [1] | 204 | 36 | 0.063 | 0.096 | 0.0531 | 8.198 | 16.397 | 2.828 | *0.9958* |
|  | Mixteca Alta | 24 | [33,34] | 99 | 14 | 0.191 | 0.228 | 0.3950 | 7.505 | 13.851 | 2.490 | *0.99175* |
|  | Zapotec | 25 | [33,34] | 81 | 17 | 0.137 | 0.176 | 0.2740 | 9.184 | 13.775 | 1.519 | 0.94536 |
|  | Mixe | 26 | [33,34] | 48 | 9 | 0.294 | 0.297 | 0.5940 | 7.398 | 11.883 | 1.895 | *0.97547* |
|  | Tarahumara | 27 | [25] | 44 | 14 | 0.244 | 0.183 | 0.8742 | 10.299 | 13.096 | 0.877 | 0.85227 |
|  | Canoncito | 28 | [1] | 40 | 8 | 0.321 | 0.319 | 0.6053 | 8.682 | 10.675 | 0.740 | 0.82191 |
|  | Sioux | 29 | [1] | 96 | 24 | 0.097 | 0.124 | 0.2407 | 8.916 | 14.267 | 1.796 | *0.96675* |
|  | Zuni | 30 | [1] | 50 | 9 | 0.200 | 0.300 | 0.1297 | 8.112 | 12.609 | 1.739 | *0.96602* |
| SAM | Guarani-Kaiowa | 31 | [1] | 144 | 10 | 0.205 | 0.334 | 0.1026 | 6.893 | 15.302 | 3.473 | *0.99941* |
|  | Guarani-Nandewa | 32 | [1] | 53 | 18 | 0.192 | 0.145 | 0.8743 | 10.313 | 14.523 | 1.296 | 0.92426 |
|  | Guarani | 33 | [35,36,37] | 32 | 4 | 0.292 | 0.539 | *0.0196* | 6.768 | 14.012 | 3.462 | ***0.99991*** |
|  | Kaingang | 34 | [35,36,37] | 28 | 4 | 0.388 | 0.531 | 0.2069 | 6.095 | 11.338 | 2.798 | *0.99835* |
|  | Ticuna | 35 | [1] | 49 | 9 | 0.221 | 0.298 | 0.2315 | 8.338 | 15.862 | 2.842 | *0.99796* |
|  | Yanomamo | 36 | [1] | 55 | 15 | 0.170 | 0.182 | 0.5279 | 9.103 | 13.417 | 1.487 | 0.9461 |
|  | Venezuelans mestisos | 37 | 12WS | 26 | 30 | 0.048 | 0.051 | 0.4153 | 11.286 | 17.261 | 1.820 | *0.97534* |
| NEA | Koreans #1 | 38 | [1] | 199 | 27 | 0.063 | 0.134 | ***0.0008*** | 8.381 | 19.758 | 3.851 | ***0.99978*** |
| **Region** | **Population** | **Map #** | **Reference(s)** | **N** | **k** | **F obs** | **F exp** | **p EW** | **theta S** | **theta Π** | **Tajima's D** | **p Tajima** |
| NEA | Tuva | 39 | [1] | 189 | 43 | 0.055 | 0.076 | 0.1003 | 8.908 | 19.825 | 3.504 | *0.99939* |
|  | Manchu | 40 | [39] | 172 | 30 | 0.064 | 0.113 | *0.0077* | 8.572 | 20.953 | 4.142 | ***0.99995*** |
|  | Koreans #2 | 41 | [39] | 199 | 30 | 0.055 | 0.118 | *0.0001* | 8.229 | 18.857 | 3.659 | ***0.99953*** |
|  | Japanese | 42 | [39,40] | 110 | 25 | 0.066 | 0.124 | *0.0024* | 8.042 | 17.601 | 3.493 | ***0.99959*** |
|  | Mongolian | 43 | [1] | 203 | 36 | 0.056 | 0.096 | *0.0091* | 8.052 | 18.855 | 3.790 | ***0.99975*** |
|  | Halkh | 44 | [39] | 40 | 32 | 0.047 | 0.059 | 0.0505 | 11.104 | 19.233 | 2.398 | *0.99353* |
|  | Hoton | 45 | [39] | 85 | 29 | 0.067 | 0.095 | 0.0763 | 9.807 | 19.377 | 2.967 | *0.99815* |
| SEA | Han | 46 | [4] | 617 | 120 | 0.063 | 0.030 | *0.9989* | 8.577 | 20.197 | 3.634 | *0.99937* |
|  | Yao | 47 | [45] | 63 | 21 | 0.101 | 0.128 | 0.2380 | 11.276 | 21.493 | 2.848 | *0.99769* |
|  | Malay | 48 | [1] | 54 | 20 | 0.152 | 0.128 | 0.8042 | 10.276 | 19.312 | 2.786 | *0.99747* |
|  | Hakka | 49 | [1] | 55 | 21 | 0.073 | 0.121 | *0.0066* | 10.620 | 20.454 | 2.935 | *0.99828* |
|  | Bunun | 50 | [1] | 101 | 11 | 0.158 | 0.289 | *0.0285* | 8.329 | 18.025 | 3.452 | *0.9995* |
|  | Toroko | 51 | [1] | 55 | 10 | 0.159 | 0.278 | *0.0336* | 9.482 | 15.420 | 1.970 | *0.97844* |
|  | Ami | 52 | [1] | 98 | 9 | 0.234 | 0.343 | 0.1750 | 8.030 | 13.789 | 2.125 | *0.98312* |
|  | Paiwan #1 | 53 | [1] | 51 | 12 | 0.177 | 0.226 | 0.2882 | 9.428 | 14.933 | 1.849 | *0.97239* |
|  | Yami (Tao) | 54 | [1] | 50 | 8 | 0.241 | 0.337 | 0.2031 | 8.499 | 15.997 | 2.778 | *0.99711* |
|  | Puyuma | 55 | [1] | 50 | 13 | 0.130 | 0.207 | 0.0548 | 9.657 | 16.388 | 2.214 | *0.98818* |
|  | Pazeh | 56 | [1] | 55 | 16 | 0.107 | 0.168 | *0.0462* | 10.051 | 19.527 | 2.978 | *0.99855* |
|  | Minnan | 57 | [1] | 102 | 26 | 0.086 | 0.115 | 0.1694 | 10.012 | 20.676 | 3.199 | *0.99915* |
|  | Siraya | 58 | [1] | 51 | 16 | 0.100 | 0.164 | *0.0247* | 10.390 | 18.632 | 2.528 | *0.99536* |
|  | Tsou | 59 | [1] | 51 | 10 | 0.180 | 0.272 | 0.1155 | 9.043 | 15.245 | 2.165 | *0.98679* |
|  | Saisiyat | 60 | [1] | 51 | 11 | 0.192 | 0.249 | 0.2821 | 9.813 | 17.573 | 2.511 | *0.9945* |
|  | Atayal | 61 | [1] | 106 | 11 | 0.145 | 0.291 | *0.0106* | 8.261 | 17.089 | 3.156 | *0.99853* |
|  | Rukai | 62 | [1] | 50 | 9 | 0.217 | 0.300 | 0.2156 | 9.851 | 15.224 | 1.736 | *0.96608* |
|  | Thao | 63 | [1] | 30 | 13 | 0.137 | 0.177 | 0.2307 | 11.366 | 17.587 | 1.851 | *0.97617* |
|  | Thaïs | 64 | [26] | 142 | 26 | 0.078 | 0.127 | *0.0300* | 8.680 | 19.990 | 3.786 | ***0.99987*** |
|  | Kinh | 65 | [1] | 102 | 25 | 0.135 | 0.121 | 0.7390 | 9.333 | 20.862 | 3.692 | ***0.99985*** |
|  | Muong | 66 | [1] | 83 | 19 | 0.117 | 0.156 | 0.2114 | 10.200 | 19.761 | 2.863 | *0.99744* |
|  | Paiwan #2 | 67 | [39] | 65 | 20 | 0.109 | 0.137 | 0.2664 | 8.454 | 16.331 | 2.864 | *0.99763* |
| SWA | Bahrainis | 68 | [77] | 72 | 22 | 0.094 | 0.126 | 0.1583 | 11.004 | 19.957 | 2.526 | *0.99404* |
|  | Svans | 69 | [5] | 80 | 37 | 0.075 | 0.067 | 0.7677 | 8.851 | 16.111 | 2.488 | *0.99286* |
|  | Indians (Golla) | 70 | [1] | 109 | 25 | 0.088 | 0.124 | 0.1267 | 9.061 | 18.938 | 3.236 | *0.99903* |
|  | Ashkenazi jews | 71 | [21,22,23,24] | 40 | 20 | 0.067 | 0.115 | *0.0012* | 9.893 | 16.166 | 2.062 | *0.98418* |
|  | Moroccan jews | 72 | [21,22,23,24] | 40 | 17 | 0.093 | 0.141 | *0.0386* | 9.893 | 18.651 | 2.879 | *0.99868* |
|  | Libyen jews | 73 | [21,22,23,24] | 40 | 18 | 0.106 | 0.131 | 0.2592 | 9.287 | 17.811 | 2.972 | *0.99897* |
|  | Lebanese #1 | 74 | [22,23] | 258 | 43 | 0.075 | 0.084 | 0.4358 | 7.622 | 16.083 | 3.078 | *0.99738* |
|  | Lebanese #2 | 75 | [78] | 191 | 21 | 0.149 | 0.173 | 0.4202 | 9.208 | 17.715 | 2.647 | *0.99385* |
|  | Lebanese (Arabs) | 76 | [78] | 95 | 21 | 0.185 | 0.146 | 0.8434 | 10.478 | 17.155 | 1.930 | *0.9755* |
|  | Turks | 77 | [1] | 245 | 35 | 0.054 | 0.105 | *0.0012* | 7.828 | 17.269 | 3.362 | *0.99875* |
| AUS | Australian aborigines (Kimberley) | 78 | [1] | 41 | 12 | 0.158 | 0.212 | 0.2076 | 8.237 | 14.640 | 2.489 | *0.99439* |
| **Region** | **Population** | **Map #** | **Reference(s)** | **N** | **k** | **F obs** | **F exp** | **p EW** | **theta S** | **theta Π** | **Tajima's D** | **p Tajima** |
| AUS | Australian aborigines  (Cape York) | 79 | [1] | 99 | 22 | 0.161 | 0.140 | 0.7601 | 9.210 | 15.792 | 2.139 | *0.98428* |
|  | Australian aborigines (Yuendumu) | 80 | [48] | 190 | 14 | 0.188 | 0.259 | 0.2452 | 6.906 | 14.077 | 2.905 | *0.99658* |
| OTH | Brasilians (european or african descent) | 81 | [1] | 99 | 37 | 0.048 | 0.074 | *0.0164* | 9.040 | 17.481 | 2.791 | *0.99672* |
| EUR | Azoreans | 82 | [16] | 231 | 40 | 0.071 | 0.088 | 0.2564 | 8.046 | 19.779 | 4.088 | ***0.99988*** |
|  | Croatians #1 | 83 | [54,65] | 104 | 27 | 0.079 | 0.111 | 0.1111 | 8.626 | 14.410 | 1.990 | *0.97657* |
|  | Croatians #2 | 84 | [54,79] | 139 | 30 | 0.061 | 0.107 | *0.0059* | 8.544 | 17.253 | 2.962 | *0.99724* |
|  | Spanish #1 | 85 | [49] | 100 | 31 | 0.079 | 0.092 | 0.3289 | 9.024 | 18.350 | 3.086 | *0.99841* |
|  | Spanish #2 | 86 | [49,51] | 72 | 31 | 0.063 | 0.082 | 0.1328 | 9.561 | 17.348 | 2.505 | *0.99379* |
|  | French | 87 | [49] | 234 | 36 | 0.069 | 0.100 | 0.0978 | 8.025 | 17.827 | 3.420 | *0.99918* |
|  | Greeks | 88 | [17,52] | 192 | 38 | 0.057 | 0.089 | *0.0320* | 8.274 | 16.100 | 2.686 | *0.99447* |
|  | Pomaki | 89 | [17] | 100 | 18 | 0.103 | 0.175 | *0.0312* | 8.854 | 17.215 | 2.816 | *0.99674* |
|  | Irish | 90 | [1] | 1000 | 33 | 0.111 | 0.151 | 0.2255 | 7.215 | 20.121 | 4.636 | ***0.99998*** |
|  | Sardinians | 91 | [49,67] | 80 | 21 | 0.099 | 0.138 | 0.1421 | 9.205 | 17.266 | 2.664 | *0.99569* |
|  | Italians | 92 | [49] | 99 | 27 | 0.079 | 0.109 | 0.1226 | 8.869 | 17.468 | 2.894 | *0.99761* |
|  | Polish | 93 | [54,68,69] | 98 | 38 | 0.072 | 0.070 | 0.6214 | 9.056 | 18.162 | 3.008 | *0.99814* |
| EUR | Portuguese | 94 | [18] | 145 | 36 | 0.071 | 0.087 | 0.2582 | 9.933 | 20.732 | 3.187 | *0.9985* |
|  | Czechs #1 | 95 | [54] | 36 | 20 | 0.079 | 0.110 | 0.0664 | 10.935 | 19.358 | 2.548 | *0.9958* |
|  | Czech Gypsies | 96 | [54] | 33 | 19 | 0.090 | 0.113 | 0.1957 | 10.296 | 18.054 | 2.507 | *0.99563* |
|  | Czechs #2 | 97 | [1] | 103 | 30 | 0.078 | 0.097 | 0.2509 | 9.318 | 18.728 | 3.016 | *0.99802* |
|  | Romanians | 98 | [49,81] | 99 | 24 | 0.074 | 0.126 | *0.0161* | 9.040 | 17.669 | 2.853 | *0.99717* |
|  | Slovenians | 99 | [1] | 100 | 25 | 0.078 | 0.120 | 0.0558 | 8.854 | 17.351 | 2.862 | *0.99682* |
| PAC | Moluccans | 100 | [1] | 40 | 11 | 0.252 | 0.233 | 0.6980 | 9.287 | 15.176 | 2.053 | *0.98428* |
|  | East Timorese | 101 | [1] | 83 | 18 | 0.264 | 0.166 | 0.9422 | 8.617 | 14.850 | 2.183 | *0.98605* |
|  | Papua New Guinea Lowlanders | 102 | [1] | 80 | 5 | 0.408 | 0.515 | 0.3152 | 5.310 | 9.034 | 2.028 | *0.97823* |
|  | Papua New Guinea Highlanders | 103 | [1] | 87 | 16 | 0.143 | 0.192 | 0.2217 | 9.070 | 15.070 | 1.997 | *0.97787* |
|  | Maori | 104 | [56] | 200 | 31 | 0.104 | 0.115 | 0.4668 | 7.918 | 18.204 | 3.667 | ***0.99969*** |
|  | Ivatan | 105 | [1] | 50 | 12 | 0.238 | 0.225 | 0.6696 | 9.851 | 18.005 | 2.634 | *0.99626* |
|  | Filipinos | 106 | [1] | 94 | 20 | 0.235 | 0.153 | 0.9303 | 9.121 | 16.803 | 2.529 | *0.99345* |

**References**

1. Mack S, Tsai Y, Sanchez-Mazas A, Erlich H (2006) 13th International Histocompatibility Workshop Anthropology / Human Genetic Diversity Joint Report - Chapter 3: Anthropology / human genetic diversity population reports. In: Hansen J, editor. Immunobiology of the Human MHC: Proceedings of the 13th International Histocompatibility Workshop and Conference. Seattle, WA: IHWG Press. pp. 580-652.

2. Spinola H, Bruges-Armas J, Middleton D, Brehm A (2005) HLA polymorphisms in Cabo Verde and Guine-Bissau inferred from sequence-based typing. Hum Immunol 66: 1082-1092.

3. Cao K, Moormann AM, Lyke KE, Masaberg C, Sumba OP, et al. (2004) Differentiation between African populations is evidenced by the diversity of alleles and haplotypes of HLA class I loci. Tissue Antigens 63: 293-325.

4. Yang G, Deng YJ, Hu SN, Wu DY, Li SB, et al. (2006) HLA-A, -B, and -DRB1 polymorphism defined by sequence-based typing of the Han population in Northern China. Tissue Antigens 67: 146-152.

5. Sanchez-Velasco P, Leyva-Cobian F (2001) The HLA class I and class II allele frequencies studied at the DNA level in the Svanetian population (Upper Caucasus) and their relationships to Western European populations. Tissue Antigens 58: 223-233.

6. Shankarkumar U, Sridharan B (2004) HLA DRB1* and DQB1* allelic diversity among Nadars: a primitive south Indian Dravidian caste group. Hum Immunol 65: 847-854.

7. Shankarkumar U, Sridharan B, Pitchappan RM (2003) HLA diversity among Nadars, a primitive Dravidian caste of South India. Tissue Antigens 62: 542-547.

8. Shankarkumar U (2004) HLA-A, -B and -Cw Allele Frequencies in a Pawra Population from Khandesh, India. Hum Immunol 65: 932-933.

9. Shankarkumar U, Ghosh K, Gupte S, Mukerjee M, Mohanty D (1999) Distribution of HLA antigens in Bhils and Pawars of Dhadgaon, Maharashtra, India. J Human Ecology 10: 173-178.

10. Shankarkumar U (2004) HLA-A, -B and -Cw Allele Frequencies in a Maratha Population from Mumbai, India. Hum Immunol 65: 954-956.

11. Shankarkumar U, Ghosh K, Mohanty D (2001) HLA antigen distribution in Maratha community from Mumbai. Inter J Human Genetics 1: 173-177.

12. Shankarkumar U (2002) HLA A19 and B14 frequencies among Parsis from Mumbai. MHC. pp. 71-77.

13. Shankarkumar U (2004) HLA-A, -B and -Cw Allele Frequencies in a Parsi Population from Western India. Hum Immunol 65: 992-993.

14. Mohyuddin A, Williams F, Mansoor A, Mehdi SQ, Middleton D (2003) Distribution of HLA-A alleles in eight ethnic groups from Pakistan. Tissue Antigens 61: 286-291.

15. Qasim Medhi S, Mohyuddin A, Qamar R, Khaliq S (1997) INPA (India and Pakistan) regional report: HLA Class I polymorphism in different Pakistani ethnic groups. In: Charron D, editor. Proc 12th Int Histocompatibility Workshop and Conference: Paris:EDK. pp. 321-322.

16. Spinola H, Brehm A, Bettencourt B, Middleton D, Bruges-Armas J (2005) HLA class I and II polymorphisms in Azores show different settlements in Oriental and Central islands. Tissue Antigens 66: 217-230.

17. Stavropoulos-Giokas C, Papasteriades C, Polymenidis A, Naumova E, Sarahuan G, et al. (1997) HLA in MEDI Region populations : 12th International Histocompatibility Workshop MEDI Region report. In: Charron D, editor. Proc 12th Int Histocompatibility Workshop and Conference: Paris:EDK. pp. 323-327.

18. Spinola H, Middleton D, Brehm A (2005) HLA genes in Portugal inferred from sequence-based typing: in the crossroad between Europe and Africa. Tissue Antigens 66: 26-36.

19. Shankarkumar U (2004) HLA-A, -B and -Cw Allele Frequencies in a Bhil Population from Western India. Hum Immunol 65: 960-961.

20. Farjadian S, Naruse T, Kawata H, Ghaderi A, Bahram S, et al. (2004) Molecular analysis of HLA allele frequencies and haplotypes in Baloch of Iran compared with related populations of Pakistan. Tissue Antigens 64: 581-587.

21. Roitberg-Tambur A, Witt CS, Friedmann A, Safirman C, Sherman L, et al. (1995) Comparative analysis of HLA polymorphism at the serologic and molecular level in Moroccan and Ashkenazi Jews. Tissue Antigens 46: 104-110.

22. Bias W, Gazit E (1997) 12th International Histocompatibility Workshop: Anthropology SWAS regional report. In: Charron D, editor. Proc 12th Int Histocompatibility Workshop and Conference: Paris:EDK. pp. 353-363.

23. Bias W, Nuwayri-Salti N, Wood W, Cissell B, Berger S, et al. (1997) A comparison of HLA-DRB1 and -DQB1 alleles in three Lebanese villages reproductively isolated by geography and religion. In: Charron D, editor. Proc 12th Int Histocompatibility Workshop and Conference: Paris:EDK. pp. 189-191.

24. Amar A, Kwon OJ, Motro U, Witt CS, Bonne-Tamir B, et al. (1999) Molecular analysis of HLA class II polymorphisms among different ethnic groups in Israel. Hum Immunol 60: 723-730.

25. Garcia-Ortiz JE, Sandoval-Ramirez L, Rangel-Villalobos H, Maldonado-Torres H, Cox S, et al. (2006) High-resolution molecular characterization of the HLA class I and class II in the Tarahumara Amerindian population. Tissue Antigens 68: 135-146.

26. Chandanayingyong D (2004) HLA-A, -B, -Cw, -DQA1, -DQB1 and -DRB1 Allele in a Population from Bangkok, Thailand. Hum Immunol 65: 1181-1183.

27. Hammond M, du Toit E, Sanchez-Mazas A, Adrien M, Coluzzi M, et al. (1997) HLA in sub-Saharan Africa: 12th International Histocompatibility Workshop SSAF report. In: Charron D, editor. Proc 12th Int Histocompatibility Workshop and Conference: Paris:EDK. pp. 345-353.

28. Migot F, Perichon B, Danze PM, Raharimalala L, Lepers JP, et al. (1995) HLA class II haplotype studies bring molecular evidence for population affinity between Madagascans and Javanese. Tissue Antigens 46: 131-135.

29. Renquin J, Sanchez-Mazas A, Halle L, Rivalland S, Jaeger G, et al. (2001) HLA class II polymorphism in Aka Pygmies and Bantu Congolese and a reassessment of HLA-DRB1 African diversity. Tissue Antigens 58: 211-222.

30. Halle L, Mbayo K, Lurhuma Z, Salmon D, Martageix C, et al. (1994) HLA-A, B, C, DR and DQ polymorphism in Zaireans. Tissue Antigens 44: 196-199.

31. Sanchez-Mazas A, Steiner Q-G, Grundschober C, Tiercy J-M (2000) The molecular determination of HLA-Cw alleles in the Mandenka (West Africa) reveals a close genetic relationship between Africans and Europeans. Tissue Antigens 56: 303-312.

32. Tiercy J-M, Sanchez-Mazas A, Excoffier L, Shi X, Jeannet M, et al. (1992) HLA-DR Polymorphism in a Senegalese Mandenka Population: DNA Oligotyping and Population Genetics of DRB1 Specificities. Am J Hum Genet 51: 592-608.

33. Petzl-Erler M, Gorodezky C, Layrisse Z, Klitz W, Fainboim L, et al. (1997) Anthropology report for Region Latin-America: Amerindian and admixed populations. In: Charron D, editor. Proc 12th Int Histocompatibility Workshop and Conference: Paris:EDK. pp. 337-344.

34. Hollenbach J, Thomson G, Cao K, Fernández-Viña M, Erlich H, et al. (2001) HLA Diversity, Differenciation, and Haplotype Evolution in Mesoamerican Natives. Hum Immunol 62: 378-390.

35. Petzl-Erler ML, Luz R, Sotomaior VS (1993) The HLA polymorphism of two distinctive South-American Indian tribes: the Kaingang and the Guarani. Tissue Antigens 41: 227-237.

36. Petzl-Erler ML, McDevitt HO (1994) Molecular analysis of the HLA-DRB genes in two tribes of Brazilian Indians. Hum Immunol 41: 180-184.

37. Sotomaior VS, Faucz FR, Schafhauser C, Janzen-Duck M, Boldt AB, et al. (1998) HLA-DQA1 and HLA-DQB1 alleles and haplotypes in two Brazilian Indian tribes: evidence of conservative evolution of HLA-DQ. Hum Biol 70: 789-797.

38. Hashimoto M, Kinoshita T, Yamasaki M, Tanaka H, Imanishi T, et al. (1994) Gene frequencies and haplotypic associations within the HLA region in 916 unrelated Japanese individuals. Tissue Antigens 44: 166-173.

39. Tanaka H, Kashiwase K, Ishikawa Y, Tokunaga K, Sideltseva E, et al. (1997) Distribution of HLA-A, B, and DRB1 alleles and haplotypes in North-East Asia. In: Charron D, editor. Proc 12th Int Histocompatibility Workshop and Conference: Paris:EDK. pp. 183-184.

40. Saito S, Ota S, Yamada E, Inoko H, Ota M (2000) Allele frequencies and haplotypic associations defined by allelic DNA typing at HLA class I and class II loci in the Japanese population. Tissue Antigens 56: 522-529.

41. Alexeev L, Khaitov R, Boldyreva M, Trofimov D, Guskova I, et al. (1997) HLA in some ethnic groups of the former Soviet Union (FSU). In: Charron D, editor. Proc 12th Int Histocompatibility Workshop and Conference: Paris:EDK. pp. 364-373.

42. Sartakova ML, Konenkov VI, Prokof'ev VF, Gel'fgat EL, Lamazhaa AM, et al. (1998) Frequency of the HLA-DP genes and the antigens of the HLA-A, -B, -Cw, and -DR loci in Tuvinians. Genetika 34: 1127-1133.

43. Martinez-Laso J, Sartakova M, Allende L, Konenkov V, Moscoso J, et al. (2001) HLA molecular markers in Tuvinians: a population with both Oriental and Caucasoid characteristics. Ann Hum Genet 65: 245-261.

44. Mizuki N, Ohno S, Ando H, Sato T, Imanishi T, et al. (1998) Major histocompatibility complex class II alleles in an Uygur population in the Silk Route of Northwest China. Tissue Antigens 51: 287-292.

45. Liu Y, Liu Z, Fu Y, Jia Z, Chen S, et al. (2006) Polymorphism of HLA class II genes in Miao and Yao nationalities of Southwest China. Tissue Antigens 67: 157-159.

46. Mizuki M, Ohno S, Ando H, Sato T, Imanishi T, et al. (1997) Major histocompatibility complex class II alleles in Kazak and Han populations in the Silk Route of northwestern China. Tissue Antigens 50: 527-534.

47. Hu WH, Lu J, Dong YL, Cheng BW, Tang WR, et al. (2005) Polymorphism of the DPB1 locus in Hani ethnic group of south-western China. Int J Immunogenet 32: 421-423.

48. Lester S, Cassidy S, Humphreys I, Bennett G, Hurley CK, et al. (1995) Evolution in HLA-DRB1 and major histocompatibility complex class II haplotypes of Australian aborigines. Definition of a new DRB1 allele and distribution of DRB1 gene frequencies. Hum Immunol 42: 154-160.

49. Piazza A, Lonjou C (1997) HLA in Europe and in the Mediterranean countries. In: Charron D, editor. Proc 12th Int Histocompatibility Workshop and Conference: Paris:EDK. pp. 374-384.

50. Ivanova R, Naoumova E, Lepage V, Djoulah S, Yordanov Y, et al. (1996) HLA-DRB1, DQA1, DQB1 DNA polymorphism in the Bulgarian population. Tissue Antigens 47: 122-126.

51. Martinez-Laso J, De Juan D, Martinez-Quiles N, Gomez-Casado E, Cuadrado E, et al. (1995) The contribution of the HLA-A, -B, -C and -DR, -DQ DNA typing to the study of the origins of Spaniards and Basques. Tissue Antigens 45: 237-245.

52. Papassavas E, Spyropoulou-Vlachou M, Papassavas A, Schipper R, Doxiadis I, et al. (2000) MHC Class I and Class II Phenotype, Gene, and Haplotype Frequencies in Greeks Using Molecular Typing Data. Hum Immunol 61: 615-623.

53. Arnaiz-Villena A, Martinez-Laso J, Gomez-Casado E, Diaz-Campos N, Santos P, et al. (1997) Relatedness among Basques, Portuguese, Spaniards, and Algerians studied by HLA allelic frequencies and haplotypes. Immunogenetics 47: 37-43.

54. Tiilikainen A, Fischer G, Grubic Z, Gyòdi E, Ivaskova E, et al. (1997) Anthropological features of the East European region. In: Charron D, editor. Proc 12th Int Histocompatibility Workshop and Conference: Paris:EDK. pp. 307-313.

55. Grundschober C, Sanchez-Mazas A, Excoffier L, Langaney A, Jeannet M, et al. (1994) HLA-DPB1 DNA polymorphism in the Swiss population: linkage disequilibrium with other HLA loci and population genetic affinities. Eur J Immunogenet 21: 143-157.

56. Tracey MC, Carter JM (2006) Class II HLA allele polymorphism: DRB1, DQB1 and DPB1 alleles and haplotypes in the New Zealand Maori population. Tissue Antigens 68: 297-302.

57. Nagy N, Zimdahl H, Krüger C, Anders P, Kayser M, et al. (1997) Are the Trobrianders emigrants of South-East Asia. In: Charron D, editor. Proc 12th Int Histocompatibility Workshop and Conference: Paris:EDK. pp. 185-188.

58. Zimdahl H, Schiefenhovel W, Kayser M, Roewer L, Nagy M (1999) Towards understanding the origin and dispersal of Austronesians in the Solomon Sea: HLA class II polymorphism in eight distinct populations of Asia-Oceania. Eur J Immunogenet 26: 405-416.

59. Hmida S, Gauthier A, Dridi A, Quillivic F, Genetet B, et al. (1995) HLA class II gene polymorphism in Tunisians. Tissue Antigens 45: 63-68.

60. Hors J, El Chenawi F, Djoulah S, Hafez M, Abbas F, et al. (1997) HLA in North African populations: 12th International Histocompatibility Workshop NAFR report. In: Charron D, editor. Proc 12th Int Histocompatibility Workshop and Conference: Paris:EDK. pp. 328-334.

61. Pimtanothai N, Hurley CK, Leke R, Klitz W, Johnson AH (2001) HLA-DR and -DQ polymorphism in Cameroon. Tissue Antigens 58: 1-8.

62. Migot-Nabias F, Fajardy I, Danze PM, Everaere S, Mayombo J, et al. (1999) HLA class II polymorphism in a Gabonese Banzabi population. Tissue Antigens 53: 580-585.

63. Grubic Z, Zunec R, Cecuk-Jelicic E, Kerhin-Brkljacic V, Kastelan D, et al. (1998) HLA class II gene and haplotype diversity in the population of the island of Hvar, Croatia. Coll Antropol 22: 157-168.

64. Grubic Z, Zunec R, Kerhin V, Cecuk E, Kastelan D, et al. (1997) Molecular analysis of HLA class II polymorphism on the island of Hvar-Croatia. In: Charron D, editor. Proc 12th Int Histocompatibility Workshop and Conference: Paris:EDK. pp. 203-204.

65. Grubic Z, Zunec R, Cecuk-Jelicic E, Kastelan D, Kerhin-Brkljacic V, et al. (1999) High resolution molecular typing of HLA class II region in the population of the island of Krk, Croatia. Coll Antropol 23: 577-588.

66. Sanchez-Mazas A, Djoulah S, Busson M, Le Monnier de Gouville I, Poirier J, et al. (2000) A linkage disequilibrium map of the MHC region based on the analysis of 14 loci haplotypes in 50 French families. Eur J Hum Genet 8: 33-41.

67. Contu L, Arras M, Carcassi C, La Nasa G, Mulargia M (1992) HLA structure of the Sardinian population: a haplotype study of 551 families. Tissue Antigens 40: 165-174.

68. Jungerman M, Sanchez-Mazas A, Fichna P, Ivanova R, Charron D, et al. (1997) HLA class II DRB1, DQA1 and DQB1 polymorphisms in the Polish population from Wielkopolska. Tissue Antigens 49: 624-628.

69. Jungerman M, Sanchez-Mazas A, Fichna P, Ivanova R, Charron D, et al. (1997) HLA class II anthropological study in the Polish population from Wielkopolska. In: Charron D, editor. Proc 12th Int Histocompatibility Workshop and Conference: Paris:EDK. pp. 194-196.

70. Reviron D, Andre M, Cantaloube JF, Biagini P, Chicheportiche C, et al. (1993) HLA-DRB1 and HLA-DQB1 polymorphism in Algerians from Algiers. Rev Fr Transfus Hemobiol 36: 509-516.

71. Arnaiz-Villena A, Benmamar D, Alvarez M, Diaz-Campos N, Varela P, et al. (1995) HLA allele and haplotype frequencies in Algerians. Relatedness to Spaniards and Basques. Hum Immunol 43: 259-268.

72. Hafez M, el-Shennawy FA (1986) HLA-antigens in the Egyptian population. Forensic Sci Int 31: 241-246.

73. El Chenawi F, Djoulah S, Abbas F, Mahmoud L, Lapperière J, et al. (1996) HLA class II DRB1 and DQB1 polymorphisms in the Egyptian population of Siwa. Hum Immunol 47: 142.

74. Abdennaji Guenounou B, Loueslati BY, Buhler S, Hmida S, Ennafaa H, et al. (2006) HLA class II genetic diversity in southern Tunisia and the Mediterranean area. Int J Immunogenet 33: 93-103.

75. Hajjej A, Kaabi H, Sellami MH, Dridi A, Jeridi A, et al. (2006) The contribution of HLA class I and II alleles and haplotypes to the investigation of the evolutionary history of Tunisians. Tissue Antigens 68: 153-162.

76. Fort M, de Stefano GF, Cambon-Thomsen A, Giraldo-Alvarez P, Dugoujon JM, et al. (1998) HLA class II allele and haplotype frequencies in Ethiopian Amhara and Oromo populations. Tissue Antigens 51: 327-336.

77. Almawi WY, Abou-Jaoude MM, Tamim H, Al-Harbi EM, Finan RR, et al. (2004) Distribution of HLA class II (DRB1/DQB1) alleles and haplotypes among Bahraini and Lebanese Arabs. Transplant Proc 36: 1844-1846.

78. Samaha H, Rahal EA, Abou-Jaoude M, Younes M, Dacchache J, et al. (2003) HLA class II allele frequencies in the Lebanese population. Mol Immunol 39: 1079-1081.

79. Grubic Z, Zunec R, Naipal A, Kastelan A, Giphart MJ (1995) Molecular analysis of HLA class II polymorphism in Croatians. Tissue Antigens 46: 293-298.

80. Comas D, Mateu E, Calafell F, Pérez-Lezaun A, Bosch E, et al. (1998) HLA class I and class II DNA typing and the origin of Basques. Tissue Antigens 51: 30-40.

81. Reed E, Ho E, Lupu F, McManus P, Vasilescu R, et al. (1992) Polymorphism of HLA in the Romanian population. Tissue Antigens 39: 8-13.
