# Supplementary material for: HLA DNA Sequence Variation among Human Populations: Molecular Signatures of Demographic and Selective Events
Source: PLoS One. 2011 Feb 1;6(2):e14643. doi: 10.1371/journal.pone.0014643 (PMC3051395; doi:10.1371/journal.pone.0014643)
Supplement: File S2 — Nucleotide diversity and heterozygosity within populations. (0.09 MB DOC) [file pone.0014643.s002.doc]

# Supporting Information S2 – Nucleotide diversity and heterozygosity within populations

| **Class I** | **HLA-A** | | | **HLA-B** | | | **HLA-Cw** | | |  |  |  |
| --- | --- | --- | --- | --- | --- | --- | --- | --- | --- | --- | --- | --- |
|  | n | π_n_ | h | n | π_n_ | h | n | π_n_ | h |  |  |  |
| All | 97 | 0.034 (0.005) | 0.817 (0.146) | 90 | 0.042 (0.005) | 0.908 (0.065) | 68 | 0.026 (0.002) | 0.862 (0.071) |  |  |  |
| All ^#^ | 54 | 0.037 (0.002) | 0.897 (0.033) | 50 | 0.044 (0.003) | 0.936 (0.020) | 34 | 0.025 (0.002) | 0.901 (0.024) |  |  |  |
| SAF | 12 | 0.038 (0.002) | 0.926 (0.016) | 9 | 0.046 (0.003) | 0.946 (0.014) | 8 | 0.025 (0.001) | 0.898 (0.024) |  |  |  |
| NAF | 2 | 0.041 (0.001) | 0.928 (0.002) | 2 | 0.046 (0.002) | 0.961 (0.009) | 0 | --- | --- |  |  |  |
| EUR | 8 | 0.035 (0.002) | 0.873 (0.038) | 8 | 0.044 (0.002) | 0.940 (0.017) | 4 | 0.025 (0.002) | 0.905 (0.015) |  |  |  |
| SWA | 20 | 0.036 (0.003) | 0.902 (0.023) | 21 | 0.043 (0.002) | 0.927 (0.022) | 15 | 0.025 (0.001) | 0.903 (0.028) |  |  |  |
| NEA | 3 | 0.037 (0.001) | 0.893 (0.020) | 2 | 0.048 (0.000) | 0.953 (0.009) | 2 | 0.026 (0.000) | 0.859 (0.073) |  |  |  |
| SEA | 24 | 0.031 (0.006) | 0.700 (0.182) | 23 | 0.041 (0.006) | 0.865 (0.085) | 20 | 0.026 (0.002) | 0.818 (0.092) |  |  |  |
| CSEA | 9 | 0.036 (0.001) | 0.862 (0.028) | 8 | 0.045 (0.003) | 0.934 (0.016) | 5 | 0.027 (0.001) | 0.894 (0.023) |  |  |  |
| TW | 13 | 0.028 (0.006) | 0.57 (0.15) | 13 | 0.037 (0.005) | 0.814 (0.081) | 13 | 0.025 (0.003) | 0.781 (0.094) |  |  |  |
| PAC | 8 | 0.033 (0.006) | 0.744 (0.169) | 5 | 0.038 (0.003) | 0.902 (0.042) | 4 | 0.027 (0.0003) | 0.866 (0.027) |  |  |  |
| AUS | 4 | 0.032 (0.006) | 0.691 (0.124) | 4 | 0.040 (0.002) | 0.826 (0.049) | 4 | 0.027 (0.001) | 0.800 (0.044) |  |  |  |
| NAM | 5 | 0.029 (0.006) | 0.741 (0.125) | 5 | 0.039 (0.005) | 0.877 (0.098) | 3 | 0.026 (0.001) | 0.821 (0.082) |  |  |  |
| SAM | 4 | 0.027 (0.006) | 0.741 (0.135) | 4 | 0.036 (0.006) | 0.901 (0.079) | 3 | 0.027 (0.001) | 0.797 (0.025) |  |  |  |
|  |  |  |  |  |  |  |  |  |  |  |  |  |

| **Class II** | **HLA-DRB1** | | | **HLA-DQA1** | | | **HLA-DQB1** | | | **HLA-DPB1** | | |
| --- | --- | --- | --- | --- | --- | --- | --- | --- | --- | --- | --- | --- |
|  | n | π_n_ | h | n | π_n_ | h | n | π_n_ | h | n | π_n_ | h |
| All | 106 | 0.071 (0.012) | 0.873 (0.082) | 58 | 0.069 (0.013) | 0.750 (0.111) | 89 | 0.061 (0.015) | 0.796 (0.112) | 56 | 0.024 (0.008) | 0.724 (0.167) |
| All ^#^ | 60 | 0.077 (0.006) | 0.919 (0.028) | 35 | 0.076 (0.005) | 0.814 (0.040) | 61 | 0.069 (0.009) | 0.848 (0.040) | 34 | 0.028 (0.005) | 0.802 (0.076) |
| SAF | 9 | 0.072 (0.007) | 0.904 (0.030) | 7 | 0.070 (0.007) | 0.791 (0.049) | 10 | 0.072 (0.007) | 0.826 (0.034) | 7 | 0.030 (0.008) | 0.800 (0.134) |
| NAF | 9 | 0.080 (0.002) | 0.926 (0.015) | 3 | 0.078 (0.003) | 0.795 (0.021) | 13 | 0.073 (0.004) | 0.851 (0.042) | 0 | --- | --- |
| EUR | 18 | 0.076 (0.005) | 0.927 (0.014) | 17 | 0.077 (0.003) | 0.818 (0.041) | 22 | 0.067 (0.005) | 0.857 (0.031) | 14 | 0.028 (0.004) | 0.813 (0.055) |
| SWA | 10 | 0.072 (0.006) | 0.908 (0.041) | 2 | 0.078 (0.001) | 0.831 (0.014) | 5 | 0.068 (0.011) | 0.836 (0.055) | 3 | 0.027 (0.003) | 0.816 (0.027) |
| NEA | 8 | 0.082 (0.004) | 0.945 (0.009) | 3 | 0.082 (0.002) | 0.831 (0.013) | 5 | 0.068 (0.010) | 0.872 (0.036) | 4 | 0.025 (0.002) | 0.794 (.048) |
| SEA | 22 | 0.074 (0.010) | 0.866 (0.051) | 3 | 0.080 (0.001) | 0.837 (0.035) | 6 | 0.066 (0.005) | 0.835 (0.059) | 6 | 0.028 (0.003) | 0.777 (0.079) |
| CSEA | 6 | 0.084 (0.004) | 0.897 (0.032) | 3 | 0.080 (0.001) | 0.837 (0.035) | 6 | 0.066 (0.005) | 0.835 (0.059) | 6 | 0.028 (0.003) | 0.777 (0.079) |
| TW | 14 | 0.068 (0.007) | 0.84 (0.046) | 0 | --- | --- | 0 | --- | --- | 0 | --- | --- |
| PAC | 7 | 0.065 (0.013) | 0.770 (0.097) | 4 | 0.053 (0.004) | 0.686 (0.036) | 7 | 0.054 (0.009) | 0.787 (0.094) | 7 | 0.022 (0.010) | 0.646 (0.271) |
| AUS | 3 | 0.062 (0.003) | 0.836 (0.020) | 2 | 0.073 (0.005) | 0.777 (0.002) | 2 | 0.048 (0.010) | 0.757 (0.094) | 3 | 0.015 (0.003) | 0.641 (0.115) |
| NAM | 12 | 0.051 (0.011) | 0.777 (0.103) | 12 | 0.059 (0.015) | 0.647 (0.123) | 12 | 0.041 (0.013) | 0.668 (0.129) | 8 | 0.011 (0.006) | 0.527 (0.181) |
| SAM | 7 | 0.061 (0.008) | 0.793 (0.107) | 5 | 0.051 (0.009) | 0.583 (0.116) | 7 | 0.031 (0.009) | 0.579 (0.106) | 4 | 0.026 (0.004) | 0.650 (0.114) |
|  |  |  |  |  |  |  |  |  |  |  |  |  |

**Table S2:** Mean nucleotide diversity (π_n_) and heterozygosity (h) within populations grouped accordingly to their geographic location (SD within brackets), at each HLA locus under study. n: number of populations, ^#^: All populations minus Taiwan (TW), PAC, AUS, NAM and SAM. North Africa (NAF), sub-Saharan Africa (SAF), Europe (EUR), Southwest Asia (SWA), Northeast Asia (NEA), Southeast Asia (SEA), Continental Southeast Asia (CSEA), Taiwan aborigines (TW), Pacific (PAC), Australia (AUS), North America (NAM), and South America (SAM).
