# Supplementary material for: HLA DNA Sequence Variation among Human Populations: Molecular Signatures of Demographic and Selective Events
Source: PLoS One. 2011 Feb 1;6(2):e14643. doi: 10.1371/journal.pone.0014643 (PMC3051395; doi:10.1371/journal.pone.0014643)
Supplement: File S3 — Genetic diversity among populations and correlation coefficient between genetic and geographic distances in different geographic groups. (0.23 MB DOC) [file pone.0014643.s003.doc]

# Supporting Information S3 – Genetic diversity among populations and correlation coefficient between genetic and geographic distances in different geographic groups

|  | **Subsaharan Africa (SAF)** | | | **North Africa (NAF)** | | | **Europe (Europe)** | | |
| --- | --- | --- | --- | --- | --- | --- | --- | --- | --- |
| **Locus** | **n** | **Φ_ST_ (%)** | **r** | **n** | **Φ_ST_ (%)** | **r** | **n** | **Φ_ST_ (%)** | **r** |
| **A** | 12 | 1.92 *** | 0.282 ** | 2 | *-0.4 ns* | --- | 8 | 1.4 *** | *0.158 ns* |
| **B** | 9 | 1.45 *** | 0.527 ** | 2 | *0.44 ns* | --- | 8 | 3.27 *** | *0.259 ns* |
| **Cw** | 8 | 1.17 *** | 0.677 ** | 0 | --- | --- | 4 | 2.16 *** | --- |
| **DRB1** | 9 | 5.88 *** | 0.369 * | 9 | 0.54 *** | *-0.175 ns* | 18 | 1.94 *** | 0.259 ** |
| **DQA1** | 7 | 3.88 *** | *0.13 ns* | 3 | 0.91 * | --- | 17 | 1.24 *** | *0.101 ns* |
| **DQB1** | 10 | 2.98 *** | 0.333 ** | 13 | 1.77 *** | *0.087 ns* | 22 | 2.07 *** | *0.157 ns* |
| **DPB1** | 7 | 7.54 *** | *0.037 ns* | 0 | --- | --- | 14 | 1.61 *** | 0.298 ** |
|  |  |  |  |  |  |  |  |  |  |
|  | **Southwest Asia (SWA)** | | | **Northeast Asia (NEA)** | | | **Southeast Asia (SEA)** | | |
| **Locus** | **n** | **Φ_ST_ (%)** | **r** | **n** | **Φ_ST_ (%)** | **r** | **n** | **Φ_ST_ (%)** | **r** |
| **A** | 20 | 3.51 *** | *-0.062 ns* | 3 | 0.63 *** | --- | 24 | 9.05 *** | *0.097 ns* |
| **B** | 21 | 2.65 *** | *-0.02 ns* | 2 | 0.75 *** | --- | 23 | 5.97 *** | *0.091 ns* |
| **Cw** | 15 | 2.34 *** | *0.099 ns* | 2 | 2.54 *** | --- | 20 | 5.05 *** | *0.004 ns* |
| **DRB1** | 10 | 2.02 *** | 0.384 * | 8 | 1.22 *** | 0.389 ** | 22 | 6.29 *** | *-0.026 ns* |
| **DQA1** | 2 | *0.8 ns* | --- | 3 | *0.52 ns* | --- | 3 | 1.94 *** | --- |
| **DQB1** | 5 | 2.21 *** | *0.085 ns* | 5 | 6.07 *** | 0.683 * | 6 | 6.74 *** | *0.06 ns* |
| **DPB1** | 3 | 1.18 * | --- | 4 | 7.07 *** | --- | 6 | 5.66 *** | *0.072 ns* |
|  |  |  |  |  |  |  |  |  |  |
|  | **Continental Southeast Asia (CSEA)** | | | **Taiwan (TW)** | | | **Pacific (PAC)** | | |
| **Locus** | **n** | **Φ_ST_ (%)** | **r** | **n** | **Φ_ST_ (%)** | **r** | **n** | **Φ_ST_ (%)** | **r** |
| **A** | 9 | 1.96 *** | 0.224 *ns* | 13 | 5.69 *** | 0.05 *ns* | 8 | 8.31 *** | *-0.094 ns* |
| **B** | 8 | 1.07 *** | 0.168 *ns* | 13 | 8.21 *** | 0.416 * | 5 | 9.3 *** | 0.701 * |
| **Cw** | 5 | 2.28 *** | 0.593 * | 13 | 7.23 *** | 0.25 * | 4 | 3.75 *** | --- |
| **DRB1** | 6 | 2.82 *** | 0.005 *ns* | 14 | 8.97 *** | 0.219 *ns* | 7 | 10.65 *** | *0.131 ns* |
| **DQA1** | 3 | 1.94 *** | --- | 0 | --- | --- | 4 | 1.4 * | --- |
| **DQB1** | 6 | 6.74 *** | *0.06 ns* | 0 | --- | --- | 7 | 9 *** | *0.011 ns* |
| **DPB1** | 6 | 5.66 *** | *0.072 ns* | 0 | --- | --- | 7 | 21.01 *** | *-0.124 ns* |
|  |  |  |  |  |  |  |  |  |  |
|  | **Australia (AUS)** | | | **North America (NAM)** | | | **South America (SAM)** | | |
| **Locus** | **n** | **Φ_ST_ (%)** | **r** | **n** | **Φ_ST_ (%)** | **r** | **n** | **Φ_ST_ (%)** | **r** |
| **A** | 4 | 3.71 *** | --- | 5 (4) | 11.04 *** | --- | 4 | 22.63 *** | --- |
| **B** | 4 | 2.2 *** | --- | 5 (4) | 6.53 *** | --- | 4 | 5.95 *** | --- |
| **Cw** | 4 | 1.7 *** | --- | 3 (2) | 10.05 *** | --- | 3 | 4.32 *** | --- |
| **DRB1** | 3 | 6.11 *** | --- | 12 | 11.62 *** | *0.073 ns* | 7 | 6.28 *** | *0.348 ns* |
| **DQA1** | 2 | 2.52 * | --- | 12 | 10.78 *** | *0.109 ns* | 5 | 17.06 *** | 0.364 * |
| **DQB1** | 2 | 8.77 *** | --- | 12 | 9.03 *** | *0.227 ns* | 7 | 16.06 *** | *0.167 ns* |
| **DPB1** | 3 | 9.41 *** | --- | 8 | 6.16 *** | 0.371 ** | 4 | 4.47 *** | --- |

**Table S3**: Molecular genetic diversity among populations (Φ_ST_ in %) and correlation coefficient between genetic and geographic distances (r) in different geographic groups. In a few cases, because a precise geographic location was not available for some populations, the number of populations studied (n) is different for r calculations, as indicated within brackets. Correlation coefficients were only computed for groups including at least 5 populations (n ≥ 5).

n: number of populations, ---: not computed because of limited group size

ns: not significant (P>0.05), *: 0.01<P<0.05, **: 0.001<P<0.01, ***: P<0.001

**Figure S3. Genetic diversity among populations**

The fixation index in % (Φ_ST_ for the molecular approach and F_ST_ for the allelic approach) is illustrated in all populations, all populations minus TW, PAC, AUS, NAM and SAM (i.e. All#), and each geographic region. The number of populations (n) as well as the p-value is indicated for each population subset (in black when both indices are similarly significant, or in their respective color when p-values differ).

ns: not significant, * : 0.01 < P < 0.05, ** : 0.001 < P < 0.01, *** : P < 0.001.

North Africa (NAF), sub-Saharan Africa (SAF), Europe (EUR), Southwest Asia (SWA), Northeast Asia (NEA), Southeast Asia (SEA), Pacific (PAC), Australia (AUS), North America (NAM), South America (SAM), and Taiwan (TW).
