# Supplementary material for: HLA DNA Sequence Variation among Human Populations: Molecular Signatures of Demographic and Selective Events
Source: PLoS One. 2011 Feb 1;6(2):e14643. doi: 10.1371/journal.pone.0014643 (PMC3051395; doi:10.1371/journal.pone.0014643)
Supplement: File S4 — Comparisons of Reynolds genetic distance matrixes. (0.86 MB DOC) [file pone.0014643.s004.doc]

# Supporting Information S4 – Comparisons of Reynolds genetic distance matrixes

**HLA-A**

| **Region** | **Population** | **Molecular > allelic^1^** | **Molecular < allelic^2^** | **Mean of distance ratio^3^** | **SD of distance ratio^3^** |
| --- | --- | --- | --- | --- | --- |
| NAF | Metalsa | 18.6% | 79.4% | 0.779 | 0.335 |
|  | Chaouya | 36.1% | 61.9% | 0.908 | 0.496 |
| SAF | Zulu | 52.6% | 46.4% | 1.046 | 0.293 |
|  | Cabo Verdeans (Northeast) | 51.5% | 47.4% | 1.000 | 0.376 |
|  | Cabo Verdeans (Southwest) | 30.9% | 67.0% | 1.067 | 0.862 |
|  | Guineans | 22.7% | 76.3% | 0.840 | 0.261 |
|  | Kenyans | 35.1% | 63.9% | 0.918 | 0.314 |
|  | Kenyans (Luo) | 39.2% | 59.8% | 0.945 | 0.364 |
|  | Kenyans (Nandi) | 32.0% | 67.0% | 0.877 | 0.425 |
|  | Dogons | 17.5% | 81.4% | 0.831 | 0.208 |
|  | Ugandans | 35.1% | 62.9% | 0.898 | 0.483 |
|  | Mandenka | 9.3% | 89.7% | 0.767 | 0.198 |
|  | Zambians | 94.8% | 4.1% | 1.395 | 0.312 |
|  | Shona | 44.3% | 54.6% | 1.018 | 0.268 |
| NAM | Yupik | 56.7% | 42.3% | 1.012 | 0.223 |
|  | Seri | 50.5% | 48.5% | 1.007 | 0.229 |
|  | Mexicans | 41.2% | 57.7% | 0.962 | 0.406 |
|  | Pima | 74.2% | 24.7% | 1.240 | 0.304 |
|  | Amerindians | 36.1% | 62.9% | 0.862 | 0.353 |
| SAM | Brasilians | 24.7% | 72.2% | 0.743 | 0.388 |
|  | Guarani-Kaiowa | 54.6% | 44.3% | 1.029 | 0.223 |
|  | Guarani-Nandewa | 44.3% | 54.6% | 0.949 | 0.213 |
|  | Bari | 93.8% | 5.2% | 1.513 | 0.285 |
| NEA | Koreans | 37.1% | 61.9% | 0.877 | 0.404 |
|  | Buriat | 44.3% | 54.6% | 1.043 | 0.524 |
|  | Tuva | 33.0% | 66.0% | 0.857 | 0.364 |
| SEA | Chinese (Han) #1 | 27.8% | 69.1% | 0.780 | 0.441 |
|  | Chinese | 24.7% | 74.2% | 0.676 | 0.425 |
|  | Chinese (Han) #2 | 45.4% | 53.6% | 0.994 | 0.489 |
|  | Okinawans | 24.7% | 74.2% | 0.817 | 0.415 |
|  | Ryukuans | 41.2% | 57.7% | 0.966 | 0.413 |
|  | Malay | 43.3% | 54.6% | 0.955 | 0.483 |
|  | Chinese (Han) #3 | 28.9% | 68.0% | 0.812 | 0.512 |
| **Region** | **Population** | **Molecular > allelic^1^** | **Molecular < allelic^2^** | **Mean of distance ratio^3^** | **SD of distance ratio^3^** |
| SEA | Singapore (Chinese descent) | 29.9% | 69.1% | 0.826 | 0.474 |
|  | Hakka | 39.2% | 59.8% | 0.985 | 0.704 |
|  | Bunun | 29.9% | 69.1% | 0.886 | 0.251 |
|  | Toroko | 26.8% | 72.2% | 0.843 | 0.286 |
|  | Ami | 16.5% | 82.5% | 0.815 | 0.237 |
|  | Paiwan | 88.7% | 10.3% | 1.194 | 0.152 |
|  | Yami (Tao) | 71.1% | 27.8% | 1.150 | 0.318 |
|  | Puyuma | 54.6% | 44.3% | 0.983 | 0.257 |
|  | Pazeh | 36.1% | 62.9% | 0.925 | 0.346 |
|  | Minnan | 24.7% | 72.2% | 0.801 | 0.668 |
|  | Siraya | 34.0% | 64.9% | 0.893 | 0.313 |
|  | Tsou | 81.4% | 17.5% | 1.118 | 0.234 |
|  | Saisiyat | 57.7% | 41.2% | 0.993 | 0.341 |
|  | Atayal | 58.8% | 40.2% | 0.992 | 0.274 |
|  | Rukai | 69.1% | 29.9% | 1.044 | 0.226 |
|  | Thao | 55.7% | 43.3% | 0.994 | 0.335 |
|  | Thaïs | 16.5% | 82.5% | 0.607 | 0.396 |
| SWA | Tamil | 27.8% | 71.1% | 0.782 | 0.334 |
|  | Kurdish | 27.8% | 71.1% | 0.794 | 0.381 |
|  | Svans | 48.5% | 50.5% | 0.975 | 0.445 |
|  | Georgians | 34.0% | 64.9% | 0.936 | 0.840 |
|  | Indians (Golla) | 32.0% | 67.0% | 0.794 | 0.362 |
|  | Nadars | 41.2% | 57.7% | 0.996 | 0.390 |
|  | Pawra | 7.2% | 91.8% | 0.534 | 0.306 |
|  | Marathas | 21.6% | 77.3% | 0.713 | 0.305 |
|  | Indians (New Delhi) | 38.1% | 60.8% | 0.899 | 0.393 |
|  | Parsi | 68.0% | 30.9% | 1.172 | 0.307 |
|  | Druzes | 23.7% | 75.3% | 0.779 | 0.396 |
|  | Israeli jews | 46.4% | 52.6% | 0.958 | 0.400 |
|  | Omani | 38.1% | 60.8% | 0.851 | 0.363 |
|  | Baloch | 54.6% | 44.3% | 1.139 | 0.527 |
|  | Pathans | 60.8% | 38.1% | 1.241 | 0.567 |
|  | Hazara | 24.7% | 74.2% | 0.817 | 0.392 |
|  | Parsi | 83.5% | 15.5% | 1.501 | 0.487 |
|  | Sindhi | 36.1% | 62.9% | 0.782 | 0.465 |
|  | Kalash | 59.8% | 39.2% | 1.103 | 0.399 |
|  | Burusho | 40.2% | 58.8% | 0.952 | 0.435 |
| AUS | Australian aborigines (Kimberley) | 29.9% | 69.1% | 0.938 | 0.161 |
|  | Australian aborigines (Cape York) | 22.7% | 76.3% | 0.777 | 0.309 |
|  |  |  |  |  |  |
| **Region** | **Population** | **Molecular > allelic^1^** | **Molecular < allelic^2^** | **Mean of distance ratio^3^** | **SD of distance ratio^3^** |
| AUS | Australian aborigines (Groote Eylandt) | 17.5% | 81.4% | 0.783 | 0.267 |
|  | Australian aborigines (Yuendumu) | 8.2% | 90.7% | 0.704 | 0.192 |
| OTH | Brasilians (european and african descent) | 41.2% | 57.7% | 0.982 | 0.529 |
|  | Cubans (european or african descent) | 19.6% | 75.3% | 0.781 | 0.363 |
|  | Cubans (european descent) | 24.7% | 72.2% | 0.783 | 0.424 |
|  | North Americans (european descent) | 40.2% | 59.8% | 0.915 | 0.458 |
|  | North Americans (african descent) | 26.8% | 72.2% | 0.894 | 0.805 |
|  | North Americans (asiatic descent) | 27.8% | 71.1% | 0.762 | 0.430 |
|  | North Americans (hispanic descent) | 43.3% | 55.7% | 0.908 | 0.412 |
| EUR | Azoreans | 25.8% | 73.2% | 0.814 | 0.448 |
|  | Croatians | 23.7% | 74.2% | 0.877 | 0.862 |
|  | Finnish | 19.6% | 80.4% | 0.757 | 0.279 |
|  | Greeks | 23.7% | 74.2% | 0.750 | 0.421 |
|  | Irish | 36.1% | 62.9% | 0.913 | 0.352 |
|  | Portuguese | 20.6% | 78.4% | 0.749 | 0.428 |
|  | Czechs | 10.3% | 88.7% | 0.634 | 0.307 |
|  | Greek Cypriots | 23.7% | 75.3% | 0.760 | 0.479 |
| PAC | Indonesians | 41.2% | 56.7% | 0.935 | 0.520 |
|  | Moluccans | 24.7% | 74.2% | 0.787 | 0.331 |
|  | East Timorese | 76.3% | 22.7% | 1.204 | 0.425 |
|  | Papua New Guinea Lowlanders | 23.7% | 75.3% | 0.850 | 0.250 |
|  | Papua New Guinea Highlanders | 64.9% | 34.0% | 1.014 | 0.221 |
|  | Samoans | 37.1% | 61.9% | 0.918 | 0.341 |
|  | Ivatan | 57.7% | 41.2% | 1.074 | 0.407 |
|  | Filipinos | 41.2% | 57.7% | 0.967 | 0.389 |

^1^: percentage of distances that are greater with the molecular approach compared to the allelic approach for the listed population.

^2^: percentage of distances that are smaller with the molecular approach compared to the allelic approach for the listed population.

^3^: Mean and standard deviation of the ratio of molecular to allelic distances for the listed population. This is a measure of the extent of the differences between the molecular (toward higher values when the mean ratio is > 1) and allelic approach (toward higher values when the mean ratio is < 1).

**HLA-B**

| **Region** | **Population** | **Molecular > allelic^1^** | **Molecular < allelic^2^** | **Mean of distance ratio^3^** | **SD of distance ratio^3^** |
| --- | --- | --- | --- | --- | --- |
| NAF | Metalsa | 84.4% | 14.4% | 1.772 | 0.730 |
|  | Chaouya | 75.6% | 23.3% | 1.385 | 0.632 |
| SAF | Zulu | 64.4% | 34.4% | 1.200 | 0.387 |
|  | Kenyans | 58.9% | 40.0% | 1.164 | 0.456 |
|  | Kenyans (Luo) | 63.3% | 35.6% | 1.152 | 0.385 |
|  | Kenyans (Nandi) | 65.6% | 33.3% | 1.203 | 0.462 |
|  | Dogons | 30.0% | 68.9% | 0.870 | 0.394 |
|  | Ugandans | 43.3% | 55.6% | 1.060 | 0.504 |
|  | Mandenka | 27.8% | 71.1% | 0.864 | 0.382 |
|  | Zambians | 45.6% | 53.3% | 1.023 | 0.472 |
|  | Shona | 26.7% | 72.2% | 0.865 | 0.309 |
| NAM | Yupik | 75.6% | 23.3% | 1.370 | 0.399 |
|  | Seri | 8.9% | 90.0% | 0.654 | 0.230 |
|  | Mexicans | 73.3% | 25.6% | 1.536 | 0.746 |
|  | Pima | 64.4% | 34.4% | 1.143 | 0.320 |
|  | Amerindians | 32.2% | 66.7% | 0.919 | 0.362 |
| SAM | Brasilians | 50.0% | 47.8% | 1.101 | 0.574 |
|  | Guarani-Kaiowa | 83.3% | 15.6% | 1.357 | 0.367 |
|  | Guarani-Nandewa | 50.0% | 48.9% | 1.076 | 0.385 |
|  | Bari | 10.0% | 88.9% | 0.782 | 0.164 |
| NEA | Koreans | 43.3% | 55.6% | 0.999 | 0.358 |
|  | Tuva | 41.1% | 57.8% | 0.979 | 0.423 |
| SEA | Chinese (Han) #1 | 34.4% | 64.4% | 0.970 | 0.359 |
|  | Chinese | 26.7% | 72.2% | 0.903 | 0.349 |
|  | Chinese (Han) #2 | 63.3% | 35.6% | 1.141 | 0.411 |
|  | Okinawans | 36.7% | 62.2% | 0.964 | 0.326 |
|  | Malay | 61.1% | 37.8% | 1.208 | 0.511 |
|  | Chinese (Han) #3 | 26.7% | 72.2% | 0.929 | 0.428 |
|  | Singapore (Chinese descent) | 36.7% | 62.2% | 0.958 | 0.359 |
|  | Hakka | 35.6% | 62.2% | 0.964 | 0.374 |
|  | Bunun | 50.0% | 47.8% | 1.011 | 0.266 |
|  | Toroko | 88.9% | 10.0% | 1.590 | 0.421 |
|  | Ami | 87.8% | 11.1% | 1.513 | 0.379 |
|  | Paiwan | 94.4% | 4.4% | 1.507 | 0.278 |
|  | Yami (Tao) | 10.0% | 88.9% | 0.792 | 0.180 |
| **Region** | **Population** | **Molecular > allelic^1^** | **Molecular < allelic^2^** | **Mean of distance ratio^3^** | **SD of distance ratio^3^** |
| SEA | Puyuma | 62.2% | 36.7% | 1.046 | 0.316 |
|  | Pazeh | 71.1% | 27.8% | 1.157 | 0.358 |
|  | Minnan | 21.1% | 76.7% | 0.906 | 0.342 |
|  | Siraya | 71.1% | 27.8% | 1.227 | 0.373 |
|  | Tsou | 65.6% | 33.3% | 1.128 | 0.298 |
|  | Saisiyat | 30.0% | 68.9% | 0.915 | 0.167 |
|  | Atayal | 87.8% | 11.1% | 1.526 | 0.429 |
|  | Rukai | 71.1% | 27.8% | 1.183 | 0.312 |
|  | Thao | 31.1% | 66.7% | 0.920 | 0.366 |
|  | Thaïs | 53.3% | 45.6% | 1.105 | 0.328 |
| SWA | Tamil | 33.3% | 65.6% | 0.920 | 0.405 |
|  | Svans | 60.0% | 38.9% | 1.253 | 0.627 |
|  | Kurdish | 60.0% | 38.9% | 1.171 | 0.588 |
|  | Georgians | 65.6% | 33.3% | 1.339 | 0.679 |
|  | Indians (Golla) | 52.2% | 46.7% | 1.072 | 0.368 |
|  | Nadars | 50.0% | 48.9% | 1.089 | 0.413 |
|  | Pawra | 55.6% | 43.3% | 1.094 | 0.319 |
|  | Marathas | 10.0% | 88.9% | 0.714 | 0.277 |
|  | Indians (New Delhi) | 38.9% | 60.0% | 0.959 | 0.442 |
|  | Parsi | 20.0% | 78.9% | 0.818 | 0.270 |
|  | Bhils | 20.0% | 78.9% | 0.770 | 0.314 |
|  | Baloch | 63.3% | 35.6% | 1.123 | 0.442 |
|  | Ashkenazi jews | 27.8% | 71.1% | 0.849 | 0.410 |
|  | Morrocan jews | 83.3% | 15.6% | 1.466 | 0.451 |
|  | Libyan jews | 41.1% | 57.8% | 1.011 | 0.450 |
|  | Druzes | 45.6% | 53.3% | 1.017 | 0.427 |
|  | Israeli jews | 41.1% | 57.8% | 1.019 | 0.492 |
|  | Omani | 55.6% | 44.4% | 1.022 | 0.371 |
|  | Hunza-Burushaski | 30.0% | 68.9% | 0.941 | 0.769 |
|  | Sindhi | 23.3% | 76.7% | 0.829 | 0.318 |
|  | Pathans | 30.0% | 68.9% | 0.821 | 0.804 |
| AUS | Australian aborigines (Kimberley) | 20.0% | 78.9% | 0.853 | 0.185 |
|  | Australian aborigines (Cape York) | 8.9% | 90.0% | 0.776 | 0.225 |
|  | Australian aborigines (Groote Eylandt) | 38.9% | 60.0% | 0.926 | 0.234 |
|  | Australian aborigines (Yuendumu) | 22.2% | 76.7% | 0.874 | 0.203 |
| OTH | Brasilians (european or african descent) | 71.1% | 27.8% | 1.405 | 0.787 |
|  | Cubans (european or african descent) | 46.7% | 52.2% | 1.109 | 0.519 |
|  | Cubans (european descent) | 53.3% | 44.4% | 1.173 | 0.714 |
| **Region** | **Population** | **Molecular > allelic^1^** | **Molecular < allelic^2^** | **Mean of distance ratio^3^** | **SD of distance ratio^3^** |
| OTH | North Americans (european descent) | 44.4% | 54.4% | 1.003 | 0.358 |
|  | North Americans (african descent) | 45.6% | 53.3% | 1.027 | 0.370 |
|  | North Americans (asiatic descent) | 32.2% | 66.7% | 0.948 | 0.364 |
|  | North Americans (hispanic descent) | 47.8% | 51.1% | 1.089 | 0.511 |
| EUR | Azoreans | 38.9% | 60.0% | 1.014 | 0.487 |
|  | Greek Cypriots | 51.1% | 47.8% | 1.039 | 0.453 |
|  | Croatians | 28.9% | 70.0% | 0.928 | 0.427 |
|  | Finnish | 53.3% | 45.6% | 1.173 | 0.484 |
|  | Greeks | 41.1% | 57.8% | 0.974 | 0.424 |
|  | Irish | 53.3% | 45.6% | 1.116 | 0.346 |
|  | Portuguese | 52.2% | 46.7% | 1.052 | 0.459 |
|  | Czechs | 35.6% | 63.3% | 0.964 | 0.474 |
| PAC | Indonesians | 31.1% | 67.8% | 0.887 | 0.432 |
|  | Papua New Guinea Highlanders | 71.1% | 27.8% | 1.173 | 0.232 |
|  | Samoans | 88.9% | 10.0% | 1.863 | 0.567 |
|  | Ivatan | 70.0% | 28.9% | 1.150 | 0.298 |
|  | Filipinos | 30.0% | 68.9% | 0.946 | 0.383 |

^1^: percentage of distances that are greater with the molecular approach compared to the allelic approach for the listed population.

^2^: percentage of distances that are smaller with the molecular approach compared to the allelic approach for the listed population.

^3^: Mean and standard deviation of the ratio of molecular to allelic distances for the listed population. This is a measure of the extent of the differences between the molecular (toward higher values when the mean ratio is > 1) and allelic approach (toward higher values when the mean ratio is < 1).

**HLA-Cw**

| **Region** | **Population** | **Molecular > allelic^1^** | **Molecular < allelic^2^** | **Mean of distance ratio^3^** | **SD of distance ratio^3^** |
| --- | --- | --- | --- | --- | --- |
| SAF | Zulu | 48.5% | 50.0% | 1.041 | 0.305 |
|  | Kenyans | 47.1% | 51.5% | 0.980 | 0.373 |
|  | Kenyans (Luo) | 51.5% | 47.1% | 1.023 | 0.386 |
|  | Kenyans (Nandi) | 42.6% | 55.9% | 0.938 | 0.355 |
|  | Dogons | 16.2% | 82.4% | 0.777 | 0.257 |
|  | Ugandans | 36.8% | 61.8% | 0.867 | 0.368 |
|  | Zambians | 23.5% | 75.0% | 0.845 | 0.280 |
|  | Shona | 35.3% | 63.2% | 0.913 | 0.369 |
| NAM | Yupik | 60.3% | 38.2% | 1.156 | 0.360 |
|  | Tarahumara | 51.5% | 47.1% | 0.948 | 0.326 |
|  | Amerindians | 39.7% | 58.8% | 0.949 | 0.351 |
| SAM | Guarani-Kaiowa | 16.2% | 82.4% | 0.783 | 0.194 |
|  | Guarani-Nandewa | 64.7% | 33.8% | 1.105 | 0.384 |
|  | Bari | 52.9% | 45.6% | 1.051 | 0.304 |
| NEA | Koreans | 70.6% | 27.9% | 1.281 | 0.393 |
|  | Tuva | 58.8% | 39.7% | 1.209 | 0.544 |
| SEA | Chinese | 44.1% | 51.5% | 1.001 | 0.323 |
|  | Okinawans | 63.2% | 35.3% | 1.126 | 0.447 |
|  | Malay | 22.1% | 76.5% | 0.786 | 0.293 |
|  | Hakka | 54.4% | 41.2% | 1.054 | 0.374 |
|  | Bunun | 55.9% | 41.2% | 1.009 | 0.361 |
|  | Toroko | 47.1% | 51.5% | 0.983 | 0.261 |
|  | Ami | 42.6% | 55.9% | 0.973 | 0.216 |
|  | Paiwan | 86.8% | 11.8% | 1.399 | 0.357 |
|  | Yami (Tao) | 29.4% | 69.1% | 0.903 | 0.170 |
|  | Puyuma | 41.2% | 57.4% | 0.955 | 0.203 |
|  | Pazeh | 47.1% | 51.5% | 0.973 | 0.339 |
|  | Minnan | 54.4% | 41.2% | 1.070 | 0.366 |
|  | Siraya | 73.5% | 25.0% | 1.450 | 0.595 |
|  | Tsou | 58.8% | 39.7% | 1.038 | 0.327 |
|  | Saisiyat | 11.8% | 86.8% | 0.765 | 0.202 |
|  | Atayal | 23.5% | 75.0% | 0.872 | 0.264 |
|  | Rukai | 86.8% | 11.8% | 1.456 | 0.381 |
|  | Thao | 44.1% | 52.9% | 0.936 | 0.361 |
|  | Thaïs #1 | 58.8% | 39.7% | 1.055 | 0.377 |
|  | Thaïs #2 | 50.0% | 48.5% | 0.962 | 0.366 |
| **Region** | **Population** | **Molecular > allelic^1^** | **Molecular < allelic^2^** | **Mean of distance ratio^3^** | **SD of distance ratio^3^** |
| SWA | Tamil | 45.6% | 52.9% | 0.949 | 0.475 |
|  | Kurdish | 52.9% | 45.6% | 1.037 | 0.355 |
|  | Georgians | 61.8% | 36.8% | 1.108 | 0.423 |
|  | Nadars | 35.3% | 63.2% | 0.937 | 0.325 |
|  | Pawra | 0.0% | 98.5% | 0.614 | 0.166 |
|  | Marathas | 48.5% | 50.0% | 1.001 | 0.412 |
|  | Indians (New Dehli) | 38.2% | 60.3% | 0.870 | 0.316 |
|  | Parsi | 35.3% | 63.2% | 0.831 | 0.387 |
|  | Bhils | 8.8% | 89.7% | 0.681 | 0.237 |
|  | Baloch | 41.2% | 57.4% | 0.980 | 0.238 |
|  | Druzes | 70.6% | 27.9% | 1.205 | 0.366 |
|  | Israeli jews | 75.0% | 23.5% | 1.235 | 0.388 |
|  | Pathans | 14.7% | 83.8% | 0.742 | 0.360 |
|  | Hunza-Burushaski | 7.4% | 91.2% | 0.604 | 0.278 |
|  | Sindhi | 22.1% | 76.5% | 0.748 | 0.284 |
| AUS | Australian aborigines (Kimberley) | 38.2% | 60.3% | 0.937 | 0.165 |
|  | Australian aborigines (Cape York) | 80.9% | 17.6% | 1.290 | 0.323 |
|  | Australian aborigines (Groote Eylandt) | 60.3% | 38.2% | 1.034 | 0.198 |
|  | Australian aborigines (Yuendumu) | 64.7% | 33.8% | 1.092 | 0.194 |
| OTH | Brasilians (european or african descent) | 32.4% | 66.2% | 0.889 | 0.369 |
|  | North Americans (european descent) | 38.2% | 60.3% | 0.949 | 0.417 |
|  | North Americans (african descent) | 41.2% | 57.4% | 0.926 | 0.378 |
|  | North Americans (asiatic descent) | 52.9% | 45.6% | 1.053 | 0.382 |
|  | North Americans (hispanic descent) | 47.1% | 51.5% | 1.012 | 0.459 |
| EUR | Azoreans | 39.7% | 58.8% | 0.928 | 0.432 |
|  | Finnish | 39.7% | 58.8% | 0.958 | 0.457 |
|  | Irish | 57.4% | 41.2% | 1.151 | 0.429 |
|  | Czechs | 61.8% | 36.8% | 1.157 | 0.524 |
| PAC | Indonesians | 50.0% | 48.5% | 1.041 | 0.511 |
|  | Samoans | 41.2% | 57.4% | 0.971 | 0.275 |
|  | Ivatan | 44.1% | 54.4% | 0.960 | 0.271 |
|  | Filipinos | 26.5% | 72.1% | 0.809 | 0.362 |

^1^: percentage of distances that are greater with the molecular approach compared to the allelic approach for the listed population.

^2^: percentage of distances that are smaller with the molecular approach compared to the allelic approach for the listed population.

^3^: Mean and standard deviation of the ratio of molecular to allelic distances for the listed population. This is a measure of the extent of the differences between the molecular (toward higher values when the mean ratio is > 1) and allelic approach (toward higher values when the mean ratio is < 1).

**HLA-DPB1**

| **Region** | **Population** | **Molecular > allelic^1^** | **Molecular < allelic^2^** | **Mean of distance ratio^3^** | **SD of distance ratio^3^** |
| --- | --- | --- | --- | --- | --- |
| SAF | Zulu | 55.4% | 42.9% | 1.119 | 0.467 |
|  | Kenyans | 41.1% | 57.1% | 0.958 | 0.483 |
|  | Merina | 62.5% | 35.7% | 1.204 | 0.452 |
|  | Aka Pygmies | 32.1% | 66.1% | 0.862 | 0.674 |
|  | Congolese | 42.9% | 55.4% | 0.914 | 0.483 |
|  | Mandenka | 58.9% | 39.3% | 1.150 | 0.381 |
|  | Shona | 42.9% | 55.4% | 0.974 | 0.332 |
| NAM | Mixteca Alta | 33.9% | 64.3% | 0.944 | 0.509 |
|  | Zapotec | 28.6% | 69.6% | 0.850 | 0.672 |
|  | Mixe | 33.9% | 64.3% | 0.859 | 0.359 |
|  | Tarahumara | 55.4% | 42.9% | 1.310 | 0.773 |
|  | Pima | 57.1% | 39.3% | 1.178 | 0.716 |
|  | Canoncito | 73.2% | 23.2% | 1.486 | 0.862 |
|  | Sioux | 44.6% | 51.8% | 1.034 | 0.731 |
|  | Zuni | 42.9% | 55.4% | 1.048 | 0.556 |
| SAM | Guarani | 16.1% | 82.1% | 0.684 | 0.611 |
|  | Kaingang | 16.1% | 82.1% | 0.828 | 0.781 |
|  | Ticuna | 26.8% | 71.4% | 1.058 | 1.066 |
|  | Yanomamo | 14.3% | 83.9% | 0.815 | 0.905 |
| NEA | Japanese (Wajin) | 32.1% | 66.1% | 1.098 | 1.138 |
|  | Japanese | 39.3% | 58.9% | 1.159 | 1.148 |
|  | Kazakh | 44.6% | 53.6% | 1.003 | 0.414 |
|  | Tuvinians | 39.3% | 58.9% | 0.901 | 0.404 |
| SEA | Uygur | 30.4% | 67.9% | 0.867 | 0.588 |
|  | Yao | 19.6% | 76.8% | 0.879 | 0.211 |
|  | Han | 41.1% | 57.1% | 0.991 | 0.398 |
|  | Miao | 37.5% | 58.9% | 0.956 | 0.201 |
|  | Hani | 42.9% | 53.6% | 1.004 | 0.382 |
|  | Malay | 37.5% | 60.7% | 0.928 | 0.382 |
| SWA | Svans | 35.7% | 62.5% | 0.893 | 0.371 |
|  | Punjabi | 19.6% | 76.8% | 0.708 | 0.406 |
|  | Lebanese | 55.4% | 41.1% | 1.119 | 0.652 |
| AUS | Australian aborigines (Kimberley) | 67.9% | 30.4% | 1.096 | 0.223 |
|  | Australian aborigines (Cape York) | 83.9% | 14.3% | 1.282 | 0.322 |
|  | Australian aborigines (Yuendumu) | 60.7% | 37.5% | 1.115 | 0.374 |
| EUR | Belgians | 33.9% | 57.1% | 1.077 | 1.162 |
|  | Bulgarians | 41.1% | 48.2% | 1.221 | 1.331 |
| **Region** | **Population** | **Molecular > allelic^1^** | **Molecular < allelic^2^** | **Mean of distance ratio^3^** | **SD of distance ratio^3^** |
| EUR | Spanish | 41.1% | 57.1% | 0.975 | 0.501 |
|  | Finnish | 21.4% | 69.6% | 0.736 | 0.345 |
|  | French | 25.0% | 69.6% | 1.015 | 1.152 |
|  | Greeks | 48.2% | 44.6% | 1.886 | 6.664 |
|  | Pomaki | 55.4% | 42.9% | 1.030 | 0.606 |
|  | Italians | 32.1% | 64.3% | 1.908 | 6.660 |
|  | Portuguese #1 | 30.4% | 64.3% | 0.981 | 0.492 |
|  | Portuguese #2 | 32.1% | 66.1% | 0.808 | 0.374 |
|  | Czech Gypsies | 35.7% | 60.7% | 0.864 | 0.531 |
|  | Czechs | 41.1% | 55.4% | 1.201 | 1.264 |
|  | Slovenians | 21.4% | 75.0% | 0.826 | 0.399 |
|  | Swiss | 32.1% | 64.3% | 0.954 | 0.826 |
| PAC | Moluccans | 80.4% | 17.9% | 1.347 | 0.410 |
|  | East Timorese | 46.4% | 50.0% | 1.038 | 0.384 |
|  | Papua New Guinea Lowlanders | 16.1% | 82.1% | 0.825 | 0.338 |
|  | Papua New Guinea Highlanders | 30.4% | 67.9% | 0.756 | 0.422 |
|  | Maori | 58.9% | 39.3% | 1.209 | 0.500 |
|  | Trobriand Islanders | 19.6% | 78.6% | 0.914 | 0.147 |
|  | Filipinos | 83.9% | 14.3% | 1.362 | 0.355 |

^1^: percentage of distances that are greater with the molecular approach compared to the allelic approach for the listed population.

^2^: percentage of distances that are smaller with the molecular approach compared to the allelic approach for the listed population.

^3^: Mean and standard deviation of the ratio of molecular to allelic distances for the listed population. This is a measure of the extent of the differences between the molecular (toward higher values when the mean ratio is > 1) and allelic approach (toward higher values when the mean ratio is < 1).

**HLA-DQA1**

| **Region** | **Population** | **Molecular > allelic^1^** | **Molecular < allelic^2^** | **Mean of distance ratio^3^** | **SD of distance ratio^3^** |
| --- | --- | --- | --- | --- | --- |
| NAF | Moroccans | 51.7% | 46.6% | 1.318 | 0.882 |
|  | Tunisians | 58.6% | 39.7% | 1.220 | 0.751 |
|  | Egyptian Copts | 20.7% | 77.6% | 0.653 | 0.491 |
| SAF | Zulu | 41.4% | 56.9% | 0.957 | 0.556 |
|  | Cameroonese | 24.1% | 74.1% | 0.640 | 0.430 |
|  | Banzabi | 63.8% | 34.5% | 1.183 | 0.527 |
|  | Kenyans | 53.4% | 44.8% | 1.075 | 0.585 |
|  | Merina | 36.2% | 62.1% | 0.862 | 0.395 |
|  | Aka Pygmies | 10.3% | 87.9% | 0.557 | 0.386 |
|  | Shona | 69.0% | 29.3% | 1.406 | 0.636 |
| NAM | Yupik | 25.9% | 72.4% | 0.681 | 0.426 |
|  | Mexican mestisos | 58.6% | 37.9% | 1.166 | 0.562 |
|  | Lacandon | 81.0% | 17.2% | 1.136 | 0.244 |
|  | Seri | 63.8% | 32.8% | 1.180 | 0.451 |
|  | Seri | 70.7% | 24.1% | 1.275 | 0.445 |
|  | Mexicans | 65.5% | 31.0% | 1.235 | 0.558 |
|  | Mixteca Alta | 63.8% | 34.5% | 1.144 | 0.395 |
|  | Zapotec | 75.9% | 22.4% | 1.511 | 1.369 |
|  | Mixe | 84.5% | 12.1% | 1.613 | 0.627 |
|  | Canoncito | 96.6% | 1.7% | 1.757 | 0.361 |
|  | Sioux | 79.3% | 19.0% | 1.299 | 0.391 |
|  | Zuni | 91.4% | 6.9% | 1.749 | 0.605 |
| SAM | Guarani | 87.9% | 8.6% | 1.684 | 0.504 |
|  | Kaingang | 65.5% | 32.8% | 1.181 | 0.361 |
|  | Ticuna | 77.6% | 19.0% | 1.533 | 1.370 |
|  | Yanomamo | 74.1% | 24.1% | 1.369 | 0.531 |
|  | Yukpa | 81.0% | 17.2% | 1.165 | 0.271 |
| NEA | Kazakh | 53.4% | 44.8% | 1.170 | 0.869 |
|  | Halkh | 32.8% | 65.5% | 0.693 | 0.593 |
|  | Hoton | 55.2% | 43.1% | 1.040 | 0.634 |
|  | Thaïs | 32.8% | 65.5% | 0.767 | 0.482 |
|  | Uygur | 62.1% | 36.2% | 1.398 | 0.810 |
|  | Han | 46.6% | 51.7% | 0.971 | 0.508 |
| SWA | Svans | 39.7% | 58.6% | 0.911 | 0.593 |
|  | Punjabi | 39.7% | 55.2% | 0.905 | 0.684 |
| AUS | Australian aborigines (Kimberley) | 36.2% | 62.1% | 0.941 | 0.715 |
|  | Australian aborigines (Cape York) | 60.3% | 37.9% | 1.270 | 0.695 |
| **Region** | **Population** | **Molecular > allelic^1^** | **Molecular < allelic^2^** | **Mean of distance ratio^3^** | **SD of distance ratio^3^** |
| EUR | Azoreans | 22.4% | 75.9% | 0.708 | 0.540 |
|  | Belgians | 56.9% | 34.5% | 1.393 | 0.866 |
|  | Croatians #1 | 44.8% | 51.7% | 0.936 | 0.612 |
|  | Croatians #2 | 58.6% | 39.7% | 1.220 | 0.669 |
|  | Spanish #1 | 56.9% | 37.9% | 1.136 | 0.626 |
|  | Spanish #2 | 58.6% | 37.9% | 1.166 | 0.812 |
|  | French | 51.7% | 43.1% | 1.125 | 0.710 |
|  | Greeks | 39.7% | 56.9% | 0.959 | 0.732 |
|  | Pomaki | 29.3% | 69.0% | 0.823 | 0.556 |
|  | Sardinians | 34.5% | 63.8% | 0.862 | 0.603 |
|  | Italians #1 | 36.2% | 62.1% | 0.988 | 0.946 |
|  | Italians #2 | 50.0% | 48.3% | 1.178 | 1.060 |
|  | Polish | 44.8% | 48.3% | 1.131 | 0.750 |
|  | Portuguese | 46.6% | 48.3% | 1.037 | 0.782 |
|  | Czech Gypsies | 67.2% | 29.3% | 1.337 | 0.706 |
|  | Czechs | 50.0% | 46.6% | 1.002 | 0.608 |
|  | Slovenians | 43.1% | 55.2% | 0.907 | 0.597 |
| PAC | Moluccans | 55.2% | 43.1% | 1.016 | 0.450 |
|  | East Timorese | 81.0% | 17.2% | 1.458 | 0.535 |
|  | Papua New Guinea Lowlanders | 44.8% | 53.4% | 0.960 | 0.496 |
|  | Papua New Guinea Highlanders | 86.2% | 12.1% | 1.837 | 0.607 |

^1^: percentage of distances that are greater with the molecular approach compared to the allelic approach for the listed population.

^2^: percentage of distances that are smaller with the molecular approach compared to the allelic approach for the listed population.

^3^: Mean and standard deviation of the ratio of molecular to allelic distances for the listed population. This is a measure of the extent of the differences between the molecular (toward higher values when the mean ratio is > 1) and allelic approach (toward higher values when the mean ratio is < 1).

**HLA-DQB1**

| **Region** | **Population** | **Molecular > allelic^1^** | **Molecular < allelic^2^** | **Mean of distance ratio^3^** | **SD of distance ratio^3^** |
| --- | --- | --- | --- | --- | --- |
| NAF | Algerians #1 | 69.7% | 28.1% | 1.177 | 0.498 |
|  | Algerians #2 | 69.7% | 28.1% | 1.204 | 0.494 |
|  | Algerians #3 | 41.6% | 57.3% | 0.870 | 0.490 |
|  | Mozabites | 21.3% | 76.4% | 0.735 | 0.348 |
|  | Egyptians #1 | 28.1% | 70.8% | 0.813 | 0.377 |
|  | Egyptians #2 | 37.1% | 60.7% | 0.861 | 0.424 |
|  | Bedouins | 38.2% | 59.6% | 0.830 | 0.476 |
|  | Moroccans | 86.5% | 12.4% | 1.323 | 0.381 |
|  | Tunisians #1 | 67.4% | 31.5% | 1.167 | 0.466 |
|  | Jerba berbers | 44.9% | 52.8% | 0.942 | 0.410 |
|  | Matmata berbers | 64.0% | 34.8% | 1.107 | 0.509 |
|  | Tunisians #2 | 53.9% | 44.9% | 1.025 | 0.639 |
|  | Egyptian Copts | 42.7% | 56.2% | 0.940 | 0.636 |
| SAF | Zulu | 21.3% | 77.5% | 0.797 | 0.212 |
|  | Cameroonese | 15.7% | 83.1% | 0.712 | 0.275 |
|  | Oromo | 71.9% | 27.0% | 1.132 | 0.344 |
|  | Amhara | 64.0% | 34.8% | 1.041 | 0.337 |
|  | Banzabi | 16.9% | 82.0% | 0.779 | 0.218 |
|  | Kenyans | 28.1% | 70.8% | 0.782 | 0.341 |
|  | Merina | 30.3% | 68.5% | 0.837 | 0.313 |
|  | Congolese | 19.1% | 79.8% | 0.694 | 0.290 |
|  | Rwandans | 43.8% | 55.1% | 0.949 | 0.343 |
|  | Shona | 43.8% | 55.1% | 0.952 | 0.291 |
| NAM | Yupik | 56.2% | 42.7% | 0.992 | 0.282 |
|  | Mexican mestisos | 36.0% | 61.8% | 0.907 | 0.297 |
|  | Lacandon | 22.5% | 76.4% | 0.921 | 0.267 |
|  | Seri | 22.5% | 75.3% | 0.882 | 0.200 |
|  | Seri | 12.4% | 85.4% | 0.812 | 0.249 |
|  | Mexicans | 37.1% | 60.7% | 0.905 | 0.294 |
|  | Mixe | 66.3% | 32.6% | 1.093 | 0.318 |
|  | Zapotec | 70.8% | 28.1% | 1.176 | 0.367 |
|  | Mixteca Alta | 59.6% | 39.3% | 1.048 | 0.330 |
|  | Canoncito | 74.2% | 24.7% | 1.131 | 0.291 |
|  | Sioux | 50.6% | 48.3% | 1.035 | 0.300 |
|  | Zuni | 56.2% | 42.7% | 1.015 | 0.338 |
| SAM | Guarani-Kaiowa | 83.1% | 15.7% | 1.309 | 0.363 |
|  | Guarani-Nandewa | 39.3% | 59.6% | 0.907 | 0.229 |
|  | Guarani | 84.3% | 14.6% | 1.253 | 0.281 |
| **Region** | **Population** | **Molecular > allelic^1^** | **Molecular < allelic^2^** | **Mean of distance ratio^3^** | **SD of distance ratio^3^** |
| SAM | Kaingang | 80.9% | 18.0% | 1.198 | 0.214 |
|  | Ticuna | 61.8% | 37.1% | 1.047 | 0.309 |
|  | Yanomamo | 88.8% | 10.1% | 1.225 | 0.227 |
|  | Yukpa | 3.4% | 95.5% | 0.695 | 0.245 |
| NEA | Japanese (Wajin) | 51.7% | 47.2% | 1.078 | 0.408 |
|  | Japanese | 70.8% | 28.1% | 1.345 | 0.559 |
|  | Kazakh | 58.4% | 39.3% | 1.107 | 0.728 |
|  | Halkh | 49.4% | 48.3% | 1.022 | 0.584 |
|  | Hoton | 52.8% | 46.1% | 1.066 | 0.460 |
| SEA | Uygur | 65.2% | 33.7% | 1.088 | 0.477 |
|  | Han | 25.8% | 73.0% | 0.846 | 0.371 |
|  | Malay | 48.3% | 50.6% | 0.990 | 0.370 |
|  | Thaïs | 27.0% | 71.9% | 0.879 | 0.391 |
|  | Kinh | 44.9% | 53.9% | 0.969 | 0.367 |
|  | Muong | 66.3% | 32.6% | 1.058 | 0.202 |
| SWA | Bahrainis | 18.0% | 82.0% | 0.748 | 0.269 |
|  | Svans | 40.4% | 58.4% | 0.908 | 0.480 |
|  | Punjabi | 49.4% | 49.4% | 1.039 | 0.457 |
|  | Lebanese | 12.4% | 86.5% | 0.747 | 0.254 |
|  | Turks | 44.9% | 53.9% | 1.036 | 0.520 |
| AUS | Australian aborigines (Kimberley) | 87.6% | 11.2% | 1.267 | 0.194 |
|  | Australian aborigines (Cape York) | 51.7% | 47.2% | 1.012 | 0.194 |
| EUR | Azoreans | 34.8% | 64.0% | 0.767 | 0.583 |
|  | Belgians | 71.9% | 27.0% | 1.476 | 1.024 |
|  | Bulgarians | 50.6% | 48.3% | 1.016 | 0.425 |
|  | Croatians #1 | 49.4% | 50.6% | 0.986 | 0.485 |
|  | Croatians #2 | 46.1% | 51.7% | 1.002 | 0.434 |
|  | Croatians #3 | 52.8% | 46.1% | 1.160 | 0.685 |
|  | Spanish | 37.1% | 61.8% | 0.765 | 0.539 |
|  | Spanish Basques | 73.0% | 25.8% | 1.128 | 0.271 |
|  | French #1 | 49.4% | 47.2% | 1.023 | 0.647 |
|  | French #2 | 38.2% | 60.7% | 0.833 | 0.447 |
|  | Greeks | 42.7% | 56.2% | 0.925 | 0.427 |
|  | Pomaki | 34.8% | 64.0% | 0.794 | 0.422 |
|  | Sardinians | 70.8% | 28.1% | 1.158 | 0.337 |
|  | Italians #1 | 29.2% | 69.7% | 0.737 | 0.471 |
|  | Italians #2 | 34.8% | 62.9% | 0.816 | 0.464 |
|  | Polish | 29.2% | 69.7% | 0.737 | 0.449 |
|  | Portuguese #1 | 52.8% | 44.9% | 0.964 | 0.428 |
| **Region** | **Population** | **Molecular > allelic^1^** | **Molecular < allelic^2^** | **Mean of distance ratio^3^** | **SD of distance ratio^3^** |
|  | Portuguese #2 | 42.7% | 56.2% | 0.890 | 0.537 |
|  | Czechs #1 | 44.9% | 46.1% | 1.001 | 0.916 |
|  | Czech Gypsies | 10.1% | 88.8% | 0.518 | 0.358 |
|  | Czechs #2 | 48.3% | 48.3% | 0.937 | 0.514 |
|  | Slovenians | 57.3% | 41.6% | 1.077 | 0.483 |
| PAC | Moluccans | 77.5% | 21.3% | 1.149 | 0.295 |
|  | East Timorese | 92.1% | 6.7% | 1.494 | 0.390 |
|  | Papua New Guinea Lowlanders #1 | 51.7% | 47.2% | 0.997 | 0.227 |
|  | Papua New Guinea Lowlanders #2 | 25.8% | 73.0% | 0.889 | 0.162 |
|  | Papua New Guinea Highlanders | 29.2% | 69.7% | 0.860 | 0.237 |
|  | Maori | 61.8% | 37.1% | 1.117 | 0.397 |
|  | Filipinos | 91.0% | 7.9% | 1.550 | 0.465 |

^1^: percentage of distances that are greater with the molecular approach compared to the allelic approach for the listed population.

^2^: percentage of distances that are smaller with the molecular approach compared to the allelic approach for the listed population.

^3^: Mean and standard deviation of the ratio of molecular to allelic distances for the listed population. This is a measure of the extent of the differences between the molecular (toward higher values when the mean ratio is > 1) and allelic approach (toward higher values when the mean ratio is < 1).

**HLA-DRB1**

| **Region** | **Population** | **Molecular > allelic^1^** | **Molecular < allelic^2^** | **Mean of distance ratio^3^** | **SD of distance ratio^3^** |
| --- | --- | --- | --- | --- | --- |
| NAF | Algerians | 27.4% | 71.7% | 0.816 | 0.380 |
|  | Moroccans #1 | 18.9% | 79.2% | 0.784 | 0.358 |
|  | Metalsa | 21.7% | 76.4% | 0.840 | 0.302 |
|  | Chaouya | 33.0% | 66.0% | 0.886 | 0.350 |
|  | Moroccans #2 | 28.3% | 70.8% | 0.888 | 0.292 |
|  | Jerba berbers | 66.0% | 33.0% | 1.185 | 0.392 |
|  | Matmata berbers | 38.7% | 60.4% | 0.911 | 0.359 |
|  | Tunisians | 34.0% | 66.0% | 0.871 | 0.374 |
|  | Egyptian Copts | 13.2% | 85.8% | 0.580 | 0.392 |
| SAF | Zulu | 58.5% | 40.6% | 1.117 | 0.304 |
|  | Cameroonese | 25.5% | 73.6% | 0.837 | 0.276 |
|  | Cabo Verdeans (Northeast) | 57.5% | 41.5% | 1.088 | 0.397 |
|  | Cabo Verdeans (Southwest) | 51.9% | 47.2% | 1.030 | 0.305 |
|  | Guineans | 45.3% | 53.8% | 0.995 | 0.262 |
|  | Merina | 84.9% | 14.2% | 1.442 | 0.411 |
|  | Dogon | 67.0% | 32.1% | 1.189 | 0.387 |
|  | Aka Pygmies | 64.2% | 34.9% | 1.130 | 0.289 |
|  | Shona | 26.4% | 72.6% | 0.849 | 0.301 |
| NAM | Yupik | 52.8% | 46.2% | 0.971 | 0.276 |
|  | Lacandon | 95.3% | 3.8% | 1.506 | 0.301 |
|  | Seri #1 | 7.5% | 90.6% | 0.769 | 0.170 |
|  | Seri #2 | 6.6% | 91.5% | 0.746 | 0.179 |
|  | Mexicans | 38.7% | 60.4% | 0.928 | 0.318 |
|  | Mixteca Alta | 18.9% | 80.2% | 0.799 | 0.229 |
|  | Zapotec | 58.5% | 40.6% | 1.056 | 0.325 |
|  | Mixe | 14.2% | 84.9% | 0.858 | 0.242 |
|  | Tarahumara | 13.2% | 85.8% | 0.787 | 0.243 |
|  | Canoncito | 12.3% | 86.8% | 0.804 | 0.257 |
|  | Sioux | 82.1% | 17.0% | 1.493 | 0.468 |
|  | Zuni | 11.3% | 87.7% | 0.762 | 0.211 |
| SAM | Guarani-Kaiowa | 10.4% | 88.7% | 0.786 | 0.204 |
|  | Guarani-Nandewa | 12.3% | 86.8% | 0.742 | 0.243 |
|  | Guarani | 1.9% | 97.2% | 0.667 | 0.189 |
|  | Kaingang | 2.8% | 96.2% | 0.695 | 0.147 |
|  | Ticuna | 17.0% | 82.1% | 0.790 | 0.249 |
|  | Yanomamo | 26.4% | 72.6% | 0.843 | 0.283 |
|  | Venezuelans mestisos | 33.0% | 66.0% | 0.867 | 0.409 |
| NEA | Koreans #1 | 34.0% | 66.0% | 0.946 | 0.490 |
| **Region** | **Population** | **Molecular > allelic^1^** | **Molecular < allelic^2^** | **Mean of distance ratio^3^** | **SD of distance ratio^3^** |
| NEA | Tuva | 26.4% | 72.6% | 0.840 | 0.293 |
|  | Manchu | 53.8% | 45.3% | 1.084 | 0.329 |
|  | Koreans #2 | 28.3% | 70.8% | 0.883 | 0.485 |
|  | Japanese | 47.2% | 51.9% | 0.991 | 0.351 |
|  | Mongolian | 43.4% | 55.7% | 0.999 | 0.382 |
|  | Halkh | 19.8% | 79.2% | 0.785 | 0.363 |
|  | Hoton | 42.5% | 56.6% | 0.999 | 0.333 |
| SEA | Han | 59.4% | 39.6% | 1.082 | 0.291 |
|  | Yao | 27.4% | 71.7% | 0.858 | 0.260 |
|  | Malay | 76.4% | 22.6% | 1.209 | 0.332 |
|  | Hakka | 34.0% | 64.2% | 0.935 | 0.326 |
|  | Bunun | 36.8% | 62.3% | 0.906 | 0.235 |
|  | Toroko | 56.6% | 42.5% | 1.038 | 0.252 |
|  | Ami | 56.6% | 42.5% | 1.091 | 0.345 |
|  | Paiwan #1 | 83.0% | 16.0% | 1.379 | 0.352 |
|  | Yami (Tao) | 17.9% | 81.1% | 0.846 | 0.223 |
|  | Puyuma | 41.5% | 57.5% | 1.017 | 0.386 |
|  | Pazeh | 21.7% | 77.4% | 0.864 | 0.282 |
|  | Minnan | 27.4% | 70.8% | 0.901 | 0.315 |
|  | Siraya | 36.8% | 62.3% | 0.941 | 0.389 |
|  | Tsou | 68.9% | 30.2% | 1.101 | 0.279 |
|  | Saisiyat | 61.3% | 37.7% | 1.079 | 0.261 |
|  | Atayal | 34.9% | 64.2% | 0.935 | 0.245 |
|  | Rukai | 89.6% | 9.4% | 1.391 | 0.304 |
|  | Thao | 68.9% | 30.2% | 1.181 | 0.396 |
|  | Thaïs | 36.8% | 62.3% | 0.956 | 0.356 |
|  | Kinh | 50.9% | 48.1% | 0.995 | 0.239 |
|  | Muong | 23.6% | 75.5% | 0.857 | 0.301 |
|  | Paiwan #2 | 78.3% | 20.8% | 1.286 | 0.397 |
| SWA | Bahrainis | 11.3% | 87.7% | 0.704 | 0.256 |
|  | Svans | 56.6% | 42.5% | 1.179 | 0.545 |
|  | Indians (Golla) | 26.4% | 72.6% | 0.895 | 0.256 |
|  | Ashkenazi jews | 38.7% | 60.4% | 0.921 | 0.358 |
|  | Moroccan jews | 29.2% | 69.8% | 0.857 | 0.361 |
|  | Libyen jews | 51.9% | 47.2% | 1.040 | 0.291 |
|  | Lebanese #1 | 38.7% | 60.4% | 0.939 | 0.273 |
|  | Lebanese #2 | 13.2% | 85.8% | 0.672 | 0.261 |
|  | Lebanese (Arabs) | 6.6% | 92.5% | 0.630 | 0.237 |
|  | Turks | 40.6% | 58.5% | 0.943 | 0.416 |
| AUS | Australian aborigines (Kimberley) | 12.3% | 86.8% | 0.739 | 0.226 |
| **Region** | **Population** | **Molecular > allelic^1^** | **Molecular < allelic^2^** | **Mean of distance ratio^3^** | **SD of distance ratio^3^** |
| AUS | Australian aborigines (Cape York) | 16.0% | 83.0% | 0.811 | 0.223 |
|  | Australian aborigines (Yuendumu) | 80.2% | 18.9% | 1.219 | 0.284 |
| OTH | Brasilians (european or african descent) | 29.2% | 69.8% | 0.871 | 0.404 |
| EUR | Azoreans | 47.2% | 51.9% | 1.257 | 2.725 |
|  | Croatians #1 | 50.0% | 49.1% | 1.017 | 0.325 |
|  | Croatians #2 | 44.3% | 53.8% | 0.947 | 0.352 |
|  | Spanish #1 | 35.8% | 63.2% | 1.025 | 1.121 |
|  | Spanish #2 | 40.6% | 58.5% | 1.530 | 5.071 |
|  | French | 28.3% | 70.8% | 0.801 | 0.374 |
|  | Greeks | 43.4% | 55.7% | 0.958 | 0.312 |
|  | Pomaki | 27.4% | 71.7% | 0.851 | 0.284 |
|  | Irish | 39.6% | 59.4% | 0.943 | 0.324 |
|  | Sardinians | 15.1% | 84.0% | 0.723 | 0.291 |
|  | Italians | 32.1% | 67.0% | 0.884 | 0.346 |
|  | Polish | 50.0% | 49.1% | 1.050 | 0.451 |
|  | Portuguese | 30.2% | 68.9% | 0.895 | 0.406 |
|  | Czechs #1 | 41.5% | 56.6% | 1.440 | 2.948 |
|  | Czech Gypsies | 16.0% | 83.0% | 0.695 | 0.307 |
|  | Czechs #2 | 29.2% | 68.9% | 1.357 | 5.082 |
|  | Romanians | 21.7% | 77.4% | 0.785 | 0.355 |
|  | Slovenians | 26.4% | 71.7% | 0.818 | 0.354 |
| PAC | Moluccans | 57.5% | 41.5% | 1.083 | 0.304 |
|  | East Timorese | 34.0% | 65.1% | 0.916 | 0.248 |
|  | Papua New Guinea Lowlanders | 15.1% | 84.0% | 0.845 | 0.196 |
|  | Papua New Guinea Highlanders | 70.8% | 28.3% | 1.129 | 0.317 |
|  | Maori | 17.9% | 81.1% | 0.785 | 0.264 |
|  | Ivatan | 17.0% | 82.1% | 0.818 | 0.231 |
|  | Filipinos | 11.3% | 87.7% | 0.733 | 0.301 |

^1^: percentage of distances that are greater with the molecular approach compared to the allelic approach for the listed population.

^2^: percentage of distances that are smaller with the molecular approach compared to the allelic approach for the listed population.

^3^: Mean and standard deviation of the ratio of molecular to allelic distances for the listed population. This is a measure of the extent of the differences between the molecular (toward higher values when the mean ratio is > 1) and allelic approach (toward higher values when the mean ratio is < 1).
